# Investigating the Causal Relationship of C-Reactive Protein with 32 Complex Somatic and Psychiatric Outcomes: A Large-Scale Cross-Consortium Mendelian Randomization Study

**DOI:** 10.1371/journal.pmed.1001976

**Published:** 2016-06-21

**Authors:** Bram. P. Prins, Ali Abbasi, Anson Wong, Ahmad Vaez, Ilja Nolte, Nora Franceschini, Philip E. Stuart, Javier Guterriez Achury, Vanisha Mistry, Jonathan P. Bradfield, Ana M. Valdes, Jose Bras, Aleksey Shatunov, PAGE Consortium, International Stroke Genetics Consortium, Systemic Sclerosis consortium, Treat OA consortium, DIAGRAM Consortium, CARDIoGRAMplusC4D Consortium, ALS consortium, International Parkinson’s Disease Genomics Consortium, Autism Spectrum Disorder Working Group of the Psychiatric Genomics Consortium, CKDGen consortium, GERAD1 Consortium, International Consortium for Blood Pressure, Schizophrenia Working Group of the Psychiatric Genomics Consortium, Inflammation Working Group of the CHARGE Consortium, Chen Lu, Buhm Han, Soumya Raychaudhuri, Steve Bevan, Maureen D. Mayes, Lam C. Tsoi, Evangelos Evangelou, Rajan P. Nair, Struan F. A. Grant, Constantin Polychronakos, Timothy R. D. Radstake, David A. van Heel, Melanie L. Dunstan, Nicholas W. Wood, Ammar Al-Chalabi, Abbas Dehghan, Hakon Hakonarson, Hugh S. Markus, James T. Elder, Jo Knight, Dan E. Arking, Timothy D. Spector, Bobby P. C. Koeleman, Cornelia M. van Duijn, Javier Martin, Andrew P. Morris, Rinse K. Weersma, Cisca Wijmenga, Patricia B. Munroe, John R. B. Perry, Jennie G. Pouget, Yalda Jamshidi, Harold Snieder, Behrooz Z. Alizadeh

**Affiliations:** 1 Department of Epidemiology, University of Groningen, University Medical Center Groningen, Groningen, The Netherlands; 2 Department of Human Genetics, Wellcome Trust Sanger Institute, Hinxton, United Kingdom; 3 MRC Epidemiology Unit, University of Cambridge School of Clinical Medicine, Institute of Metabolic Science, Addenbrooke’s Hospital, Cambridge, United Kingdom; 4 Department of Internal Medicine, University of Groningen, University Medical Center Groningen, Groningen, the Netherlands; 5 Institute of Medical Sciences, University of Toronto, Toronto, Ontario, Canada; 6 Faculty of Medicine, University of Ottawa, Ottawa, Ontario, Canada; 7 School of Medicine, Isfahan University of Medical Sciences, Isfahan, Iran; 8 Department of Epidemiology, University of North Carolina, Chapel Hill, North Carolina, United States of America; 9 Department of Dermatology, Veterans Affairs Hospital, University of Michigan, Ann Arbor, Michigan, United States of America; 10 Department of Genetics, University of Groningen, University Medical Centre Groningen, Groningen, the Netherlands; 11 Metabolic Research Laboratories, Wellcome Trust–MRC Institute of Metabolic Science, Addenbrooke’s Hospital, University of Cambridge, Cambridge, United Kingdom; 12 Blizard Institute, Barts and The London School of Medicine and Dentistry, Queen Mary University of London, London, United Kingdom; 13 Center for Applied Genomics, Division of Human Genetics, Children’s Hospital of Philadelphia, Philadelphia, United States of America; 14 Department of Academic Rheumatology, University of Nottingham, Nottingham, United Kingdom; 15 Department of Molecular Neuroscience, Institute of Neurology, London, United Kingdom; 16 Department of Basic and Clinical Neuroscience, Institute of Psychiatry, Psychology and Neuroscience, King’s College London, London, United Kingdom; 17 Neurology Unit, Department of Clinical Neurosciences, University of Cambridge, Cambridge, United Kingdom; 18 Division of Rheumatology and Clinical Immunogenetics, University of Texas Health Science Center at Houston, Houston, Texas, United States of America; 19 Instituto de Parasitologia y Biomedicina Lopez-Neyra, Consejo Superior de Investigaciones Científicas, Granada, Spain; 20 Department of Hygiene and Epidemiology, University of Ioannina Medical School, Ioannina, Greece; 21 Wellcome Trust Centre for Human Genetics, University of Oxford, Oxford, United Kingdom; 22 Institut für Integrative und Experimentelle Genomik, Universität zu Lübeck, Lübeck, Germany; 23 Department of Molecular Neuroscience, UCL Institute of Neurology, London, United Kingdom; 24 McKusick-Nathans Institute of Genetic Medicine and Department of Medicine, Division of Cardiology, Johns Hopkins University School of Medicine, Baltimore, Maryland, United States of America; 25 NHLBI’s Framingham Heart Study, Center for Population Studies and Harvard Medical School, Framingham, Massachusetts, United States of America; 26 Institute of Psychological Medicine and Clinical Neurosciences, School of Medicine, Cardiff University, Cardiff, United Kingdom; 27 NIHR Barts Cardiovascular Biomedical Research Unit, William Harvey Research Institute, Queen Mary University of London, London, United Kingdom; 28 MRC Centre for Neuropsychiatric Genetics and Genomics, Institute of Psychological Medicine and Clinical Neurosciences, School of Medicine, Cardiff University, United Kingdom; 29 Department of Biostatistics, Boston University School of Public Health, Boston, Massachusetts, United States of America; 30 Program in Medical and Population Genetics, Broad Institute of Harvard and MIT, Cambridge, Massachusetts, United States of America; 31 Division of Genetics, Brigham and Women’s Hospital, Harvard Medical School, Boston, Massachusetts, United States of America; 32 Division of Rheumatology, Brigham and Women’s Hospital, Harvard Medical School, Boston, Massachusetts, United States of America; 33 Partners HealthCare Center for Personalized Genetic Medicine, Boston, Massachusetts, United States of America; 34 Faculty of Medical and Human Sciences, University of Manchester, Manchester, United Kingdom; 35 Department of Biostatistics, University of Michigan, Ann Arbor, Michigan, United States of America; 36 Department of Epidemiology and Biostatistics, School of Public Health, Imperial College London, London, United Kingdom; 37 Department of Pediatrics, Perelman School of Medicine, University of Pennsylvania, Philadelphia, United States of America; 38 Endocrine Genetics Research Institute, McGill University Health Center, Montreal, Quebec, Canada; 39 Department of Rheumatology & Clinical Immunology and Laboratory of Translational Immunology, University Medical Center Utrecht, Utrecht, the Netherlands; 40 Complex Disease Genetics, Cold Spring Harbor Laboratory, Cold Spring Harbor, New York, United States of America; 41 Department of Epidemiology, Erasmus University Rotterdam, University Medical Centre Rotterdam, Rotterdam, the Netherlands; 42 Campbell Family Mental Health Research Institute, Centre for Addiction and Mental Health, Toronto, Ontario, Canada; 43 Department of Psychiatry, University of Toronto, Toronto, Ontario, Canada; 44 Biostatistics Division, Dalla Lana School of Public Health, University of Toronto, Toronto, Ontario, Canada; 45 Department of Twin Research and Genetic Epidemiology, King’s College London, London, United Kingdom; 46 Complex Genetic Section, Department of Medical Genetics, University Medical Center Utrecht, Utrecht, the Netherlands; 47 Department of Biostatistics, University of Liverpool, Liverpool, United Kingdom; 48 Department of Gastroenterology and Hepatology, University of Groningen, University Medical Center Groningen, Groningen, the Netherlands; 49 Clinical Pharmacology, William Harvey Research Institute, Barts and the London School of Medicine, Queen Mary University of London, London, United Kingdom; 50 Cardiogenetics Lab, Cardiovascular and Cell Sciences Institute, St George’s Hospital Medical School, London, United Kingdom; Western Sydney University, AUSTRALIA

## Abstract

**Background:**

C-reactive protein (CRP) is associated with immune, cardiometabolic, and psychiatric traits and diseases. Yet it is inconclusive whether these associations are causal.

**Methods and Findings:**

We performed Mendelian randomization (MR) analyses using two genetic risk scores (GRSs) as instrumental variables (IVs). The first GRS consisted of four single nucleotide polymorphisms (SNPs) in the CRP gene (GRS_*CRP*_), and the second consisted of 18 SNPs that were significantly associated with CRP levels in the largest genome-wide association study (GWAS) to date (GRS_*GWAS*_). To optimize power, we used summary statistics from GWAS consortia and tested the association of these two GRSs with 32 complex somatic and psychiatric outcomes, with up to 123,865 participants per outcome from populations of European ancestry. We performed heterogeneity tests to disentangle the pleiotropic effect of IVs. A Bonferroni-corrected significance level of less than 0.0016 was considered statistically significant. An observed *p-*value equal to or less than 0.05 was considered nominally significant evidence for a potential causal association, yet to be confirmed.

The strengths (*F*-statistics) of the IVs were 31.92–3,761.29 and 82.32–9,403.21 for GRS_*CRP*_ and GRS_*GWAS*_, respectively. CRP GRS_*GWAS*_ showed a statistically significant protective relationship of a 10% genetically elevated CRP level with the risk of schizophrenia (odds ratio [OR] 0.86 [95% CI 0.79–0.94]; *p <* 0.001). We validated this finding with individual-level genotype data from the schizophrenia GWAS (OR 0.96 [95% CI 0.94–0.98]; *p <* 1.72 × 10^−6^). Further, we found that a standardized CRP polygenic risk score (CRP_*PRS*_) at *p-*value thresholds of 1 × 10^−4^, 0.001, 0.01, 0.05, and 0.1 using individual-level data also showed a protective effect (OR < 1.00) against schizophrenia; the first CRP_*PRS*_ (built of SNPs with *p* < 1 × 10^−4^) showed a statistically significant (*p <* 2.45 × 10^−4^) protective effect with an OR of 0.97 (95% CI 0.95–0.99). The CRP GRS_*GWAS*_ showed that a 10% increase in genetically determined CRP level was significantly associated with coronary artery disease (OR 0.88 [95% CI 0.84–0.94]; *p* < 2.4 × 10^−5^) and was nominally associated with the risk of inflammatory bowel disease (OR 0.85 [95% CI 0.74–0.98]; *p <* 0.03), Crohn disease (OR 0.81 [95% CI 0.70–0.94]; *p <* 0.005), psoriatic arthritis (OR 1.36 [95% CI 1.00–1.84]; *p <* 0.049), knee osteoarthritis (OR 1.17 [95% CI 1.01–1.36]; *p <* 0.04), and bipolar disorder (OR 1.21 [95% CI 1.05–1.40]; *p <* 0.007) and with an increase of 0.72 (95% CI 0.11–1.34; *p <* 0.02) mm Hg in systolic blood pressure, 0.45 (95% CI 0.06–0.84; *p <* 0.02) mm Hg in diastolic blood pressure, 0.01 ml/min/1.73 m^2^ (95% CI 0.003–0.02; *p <* 0.005) in estimated glomerular filtration rate from serum creatinine, 0.01 g/dl (95% CI 0.0004–0.02; *p <* 0.04) in serum albumin level, and 0.03 g/dl (95% CI 0.008–0.05; *p <* 0.009) in serum protein level. However, after adjustment for heterogeneity, neither GRS showed a significant effect of CRP level (at *p* < 0.0016) on any of these outcomes, including coronary artery disease, nor on the other 20 complex outcomes studied. Our study has two potential limitations: the limited variance explained by our genetic instruments modeling CRP levels in blood and the unobserved bias introduced by the use of summary statistics in our MR analyses.

**Conclusions:**

Genetically elevated CRP levels showed a significant potentially protective causal relationship with risk of schizophrenia. We observed nominal evidence at an observed *p <* 0.05 using either GRS_*CRP*_ or GRS_*GWAS*_—with persistence after correction for heterogeneity—for a causal relationship of elevated CRP levels with psoriatic osteoarthritis, rheumatoid arthritis, knee osteoarthritis, systolic blood pressure, diastolic blood pressure, serum albumin, and bipolar disorder. These associations remain yet to be confirmed. We cannot verify any causal effect of CRP level on any of the other common somatic and neuropsychiatric outcomes investigated in the present study. This implies that interventions that lower CRP level are unlikely to result in decreased risk for the majority of common complex outcomes.

## Abstract 2

Using genetic instruments, Behrooz Z. Alizadeh and colleagues examine the hypothesis that increased CRP levels play a causal role in common somatic and psychiatric conditions.

## Editors' Summary

**Background:**

Inflammation is an important part of the human immune response, the network of cells and molecules that protects the body from attack by pathogens (infectious organisms) and from harmful substances and foreign particles (for example, splinters). When human cells are attacked by pathogens or injured by trauma or chemicals, molecules called inflammatory mediators induce fluid leakage from the blood vessels into the damaged tissue and attract “phagocytes” (a type of immune cell) to the site of infection or injury to “eat” the germs and dead or damaged cells. The end result is inflammation, which is characterized by swelling, redness, heat, and pain. The inflammatory response, although unpleasant, limits the damage caused by foreign invaders or chemicals by preventing further contact with body tissues. Sometimes, however, inflammation can be harmful. Persistent dysregulation of the inflammatory response is implicated in numerous somatic disorders (diseases that affect the body, such as cardiovascular disease) and neuropsychiatric disorders (mental disorders attributable to diseases of the nervous system, such as schizophrenia).

**Why Was This Study Done?:**

Observational studies suggest that increased blood levels of C-reactive protein (CRP, an inflammatory protein) are associated with certain somatic and neuropsychiatric disorders. But observational studies cannot prove that changes in CRP levels actually cause any of these disorders. It could be that the individuals who develop a specific disease and who have a high CRP level also share another unknown characteristic that is actually responsible for disease development (confounding). Alternatively, it could be that the disease itself increases CRP levels (reverse causation). It is important to know whether CRP is causally involved in the development of specific diseases because it might then be possible to prevent or treat these diseases using drugs that control CRP levels. Here, the researchers undertake a Mendelian randomization study to determine whether CRP has a causal relationship with 32 common complex somatic and neuropsychiatric outcomes. Because gene variants are inherited randomly, they are not prone to confounding and are free from reverse causation. So, if CRP levels actually cause a specific somatic or neuropsychiatric disease, genetic variants that affect CRP levels should be associated with an altered risk for that disease.

**What Did the Researchers Do and Find?:**

The researchers used data collected by several consortia involved in large genome-wide association studies (studies that ask whether specific genetic changes across the whole human genome, or blueprint, are associated with specific diseases) to look for associations between 32 somatic and neuropsychiatric outcomes and two genetic risk scores (GRSs) for CRP level. GRS_*CRP*_ consisted of four single nucleotide polymorphisms (SNPs; a type of genetic variant) in the gene encoding CRP; GRS_*GWAS*_ consisted of 18 SNPs that were associated with CRP level in a genome-wide association study. The researchers report that a genetically increased CRP level was significantly associated with a reduced risk of schizophrenia (a significant association is one unlikely to have arisen by chance). In addition, they found a nominally significant association (an association that needs to be confirmed) between genetically increased CRP levels and an increased risk of knee osteoarthritis, raised diastolic and systolic blood pressure, and bipolar disorder. Notably, there was no evidence for an effect of genetically increased CRP levels on any of the other 27 outcomes studied.

**What Do These Findings Mean?:**

These findings suggest that genetically raised levels of CRP are causally associated with protection against schizophrenia, an unexpected finding given other recent studies that suggest that raised CRP levels and brain inflammation predispose individuals to schizophrenia. The findings also provide preliminary evidence that genetically raised levels of CRP may be causally associated with an increased risk of raised blood pressure, knee arthritis, and bipolar disorder. The lack of any association between genetically raised levels of CRP and the other outcomes studied suggests, however, that many previously identified disease-associated rises in CRP levels might be a response to the disease process rather than a cause of these diseases. Like all Mendelian randomization studies, the reliability of these findings depends on the validity of several assumptions made by the researchers and on the ability of the GRSs used in the study to explain variations in CRP level. Importantly, however, these findings suggest that interventions designed to lower CRP level are unlikely to decrease the risk of people developing the majority of common complex somatic and neuropsychiatric outcomes.

**Additional Information:**

This list of resources contains links that can be accessed when viewing the PDF on a device or via the online version of the article at http://dx.doi.org/10.1371/journal.pmed.1001976.

- Wikipedia has pages on inflammation, C-reactive protein, and Mendelian randomization (note: Wikipedia is a free online encyclopedia that anyone can edit; available in several languages)
- The MedlinePlus encyclopedia has a page on C-reactive protein (in English and Spanish)
- The American Heart Association provides a short article on inflammation and heart disease
- A UK National Health Service “Behind the Headlines” article explains a recent study that found an association between immune activity in the brain and schizophrenia

## Introduction

Emerging evidence suggests that persistent dysregulation of the inflammatory response is linked to a plethora of complex somatic and neuropsychiatric disorders [1–18]. Epidemiological studies have shown that C-reactive protein (CRP), a well-studied biomarker of inflammation, is associated with and exhibits reliable predictive value for cardiovascular disease [19,20], type 2 diabetes [21], immunity-related disorders such as inflammatory bowel disease (IBD) [22], rheumatoid arthritis [23], and all-cause mortality [20,24]. Nevertheless, the evidence for a causal involvement of CRP in these outcomes from traditional experimental or observational studies remains controversial [25,26], fueling the debate surrounding whether CRP contributes to the chain of causality in disease mechanisms [27]. The use of genetically informed instrumental variables (IVs), termed Mendelian randomization (MR), is a complementary approach to epidemiological observations and allows investigation of whether the effect of an exposure (i.e., CRP level) on observed outcome phenotypes is likely to be causal [28].

Recent large-scale MR studies, focusing mainly on cardiovascular disease and metabolic traits, failed to show a causal association between CRP level and these outcomes (S1 Table). This has led to the notion that elevated CRP levels do not causally contribute to these traits and disorders. However, these studies used a single CRP-associated single nucleoid polymorphism (SNP) or a very limited set of CRP-associated SNPs (S1 Table). Common SNPs serving as proxies for CRP level represent only a small effect on CRP level per se and thus require a large enough sample size to detect causal effects on the outcome. Moreover, most studies have generally included a limited range of common complex diseases, often not more than two or three outcomes, or they have been performed in a single or small population, yielding inadequate study power (S1 Table). In other words, the evidence for a causal relationship between CRP and a broad range of common traits or diseases remains inconclusive. This is mostly due to the lack of well-powered MR studies that use optimally informative genetic IVs for CRP. Here, we sought to comprehensively examine the hypothesis that genetically determined CRP level directly contributes to common somatic and psychiatric outcomes. To optimize IV power, we applied a MR approach using summary statistics from large-scale genome-wide association study (GWAS) consortia of 32 somatic and psychiatric phenotypes for the four *CRP* variants representing 98% of the common variation in the *CRP* gene and for the largest known set of independent SNPs known to be associated with CRP. We further aimed to confirm the identified association between CRP and schizophrenia using a CRP polygenic risk score (CRP_*PRS*_) from individual-level genotype data from the largest consortium of schizophrenia to date. We performed an in silico pathway analysis (see Discussion) to provide insights into the possible mechanism underlying the observed association of CRP level with schizophrenia.

## Methods

### Study Design and Rationale

The present MR study consists of two key components. First, we used established gene variants associated with CRP level and combined them to build two genetic risk scores (GRSs) for CRP. The first GRS consisted of only four SNPs in the *CRP* gene (GRS_*CRP*_) selected from the largest recent MR study of CRP [29], and the second consisted of 18 SNPs that were associated with CRP level at a genome-wide significance level in the largest GWAS for CRP to date (GRS_*GWAS*_) [30]. Second, we obtained summary association statistics from GWAS consortia for a panel of 32 common somatic and psychiatric outcomes (Table 1). BPP and BZA selected the studies, and contacted each consortium with a standardized request for study data, including the name of the study or consortium, the number of cases and controls, the number of available CRP SNPs for GRS_*CRP*_ and GRS_*GWAS*_, and the estimated effect for each SNP (or its proxy) on outcome, i.e., the per allele regression coefficient with standard error or the odds ratio (OR) and corresponding 95% confidence interval. Data were available for 32 different outcomes in five broad disease/trait classes (autoimmune/inflammatory, cardiovascular, metabolic, neurodegenerative, and psychiatric), including at least 1,566, and up to 184,305, participants per outcome from populations of European ancestry (Table 1). These outcomes were selected based on the following two inclusion criteria: (i) the outcome having been associated with CRP level in epidemiological studies and (ii) availability of large meta-GWAS analyses for the outcome (Table 1).

**Table 1:** Diseases and traits included in this study.

Disease or Trait	Cases	Controls	Total	Reference
**Autoimmune/inflammatory**				
Celiac disease	4,533	10,750	15,283	[31]
IBD (all types)	13,020	34,774	47,794	[32,33]
Crohn disease	6,333	15,056	21,389	[32]
Ulcerative colitis	6,687	19,718	26,405	[33]
Psoriasis vulgaris	4,007	4,934	8,941	[34,35]
Psoriatic arthritis	1,946	4,934	6,880	[34,35]
Cutaneous psoriasis	1,363	3,517	4,880	[34,35]
Rheumatoid arthritis	5,538	20,167	25,705	[36]
Systemic lupus erythematous	1,311	3,340	4,651	[37]
Systemic sclerosis	2,356	5,187	7,543	[38]
Type 1 diabetes	9,934	16,956	26,890	[39]
Knee osteoarthritis	5,755	18,505	24,260	[40]
**Cardiovascular**				
Coronary artery disease	60,801	123,504	184,305	[41]
Systolic blood pressure	—	—	69,368	[42]
Diastolic blood pressure	—	—	69,372	[42]
Ischemic stroke (all types)	3,548	5,972	9,520	[43]
Ischemic stroke (cardioembolic)	790	5,972	6,762	[43]
Ischemic stroke (large vessel)	844	5,972	6,816	[43]
Ischemic stroke (small vessel)	580	5,972	6,522	[43]
**Metabolic**				
Body mass index	—	—	123,865	[44]
Type 2 diabetes	6,698	15,872	22,570	[45]
Chronic kidney disease	6,271	68,083	74,354	[46]
eGFR_**cr**_	—	—	74,354	[46]
Serum albumin level	—	—	53,189	[47]
Serum protein level	—	—	25,537	[47]
**Neurodegenerative**				
Amyotrophic lateral sclerosis	4,133	8,130	12,663	[48]
Alzheimer disease	4,663	8,357	13,020	[49]
Parkinson disease	5,333	12,019	17,352	[50]
**Psychiatric**				
Autism	90	1,476	1,566	[51]
Bipolar disorder	7,481	9,250	16,731	[52]
Major depressive disorder	9,240	9,519	18,759	[53]
Schizophrenia	34,241	45,604	79,845	[54]

eGFR_cr_, estimated glomerular filtration rate from serum creatinine.

### Genetic Instruments

Weak IVs yielding insufficient statistical power may have hampered estimation of causal effects of CRP on the outcomes in previous analyses (S1 Table). Our MR approach, by using GWAS data and combining multiple independent SNPs into a GRS (i.e., IV), has the potential to greatly increase power. The selected SNPs have been described elsewhere [30,55,56] and are further detailed in S2–S4 Tables. These IVs were used to test the combined effect of the associations of CRP-level-influencing alleles with the outcomes. Our approach was implemented in such a way that the effects of both independent SNPs in the *CRP* gene (GRS_*CRP*_) [55,56] (S1 Methods) and independent SNPs known to be genome-wide significantly associated with CRP levels (GRS_*GWAS*_) [30], as well as pleiotropic effects of SNPs, could be discriminated [57]. Pleiotropy exists if CRP SNPs influence exposures (risk factors) other than CRP level and therefore violate one of the key MR assumptions.

### Statistical Analysis

All analyses were done using the GRS function implemented in the grs.summary module of the R package Genetics ToolboX (version 2.15.1 for Windows). The grs.summary module approximates the regression of an outcome onto an additive GRS, using only single SNP association summary statistics extracted from GWAS results. The method is described in more detail elsewhere [58]. In brief, we performed MR analyses using GRS IVs in two steps. First, we used four individual *CRP* gene SNPs (i.e., IVs) associated with CRP level [56,59] (S2 and S3 Tables) to create a weighted GRS, named GRS_*CRP*,_ corresponding to the joint effect of the four SNPs within the *CRP* gene [55]. We extracted ω (the estimated coefficient, or weight) for individual SNPs from association results reported by the CRP Coronary Heart Disease Genetics Collaboration (CCGC) [29,55]; ω represents a one-unit (in mg/l) increase of the natural log of CRP level (lnCRP) per dose of the coded allele. The four tagging SNPs represent 98% of the common variation in the *CRP* gene, assuming a minor allele frequency of ≥0.05 and an *r* ^2^ threshold of ≥0.8, and aggregately explain ~2% of the total variation (i.e., phenotypic variance) in serum CRP level in populations of European descent [55,59]. Second, we constructed a multilocus GRS, named GRS_*GWAS*_, that combined 18 SNPs associated with serum CRP level at a genome-wide significance level (*p <* 5×10^−8^; S2 and S3 Tables), derived from a large meta-GWAS analysis of CRP conducted by the CHARGE (Cohorts for Heart and Aging Research in Genomic Epidemiology) Consortium [30]. This multilocus GRS explains approximately ~5% of the total variation in serum CRP level [30].

We integrated ω for each CRP SNP from the reference data of CCGC [55] or meta-analysis of GWASs [30] for CRP level with the summary association statistics extracted from the GWAS consortium data for each outcome (S1 Data; S2 Methods). This MR approach using meta-GWAS summary statistics data is equivalent to an inverse-variance-weighted meta-analysis and has previously been validated in comparison to individual-level data [57,60]. To estimate the causal effect of CRP level on an outcome, we obtained the β values (estimated effects from regression analysis) for the effects of CRP SNPs on the outcome, with standard errors, se_β_, from the corresponding GWAS results. Where no summary statistics for a CRP SNP in the GRS IVs were available in the look-up dataset, we chose the proxy SNP that had the highest linkage disequilibrium with the initial SNP (*r* ^2^ > 0.9 in HapMap release 22; S3 Table). If several proxy SNPs had the exact same *r* ^2^ value, we chose the proxy nearest to the original SNP in the instrument. Separate regressions of outcomes on GRSs were performed to calculate α_IV_ estimators (i.e., causal IV estimators) for each outcome. Correspondingly, the value of a GRS is the sum of the ω values multiplied by the allele dosage (i.e., 0, 1, or 2) for each CRP SNP in the CCGC or in the CHARGE Consortium data [30,55]. For uncorrelated SNPs, when maximizing the likelihood function, the α_IV_ value and its standard error, se_α_, can be approximated with the formula α ≅ (Σω × β × se_β_^−2^) /(Σω^2^ × se_β_^−2^), with se_α_ ≅ √1/ Σω^2^ × se_β_^−2^. lnCRP was used as the outcome in reference studies [30,55], so in obtaining the ω values (i.e., effect sizes) for each of the CRP SNPs, a unit increase in lnCRP equals a 10 symmetric percentage (s%) increase in CRP level, which corresponds to a unit change in the level of a continuous outcome or logit of risk estimate (i.e., beta coefficient) for a dichotomous outcome [61]. The α_IV_ value (i.e., causal estimate) for each CRP SNP is, therefore, presented for each outcome as corresponding to a 10-s% increase in actual CRP level. During the course of this study, an updated, larger GWAS dataset for coronary artery disease (CAD) became publicly available (CARDIoGRAMplusC4D Consortium, release 2015 [41]); we therefore redid the analysis for CAD using the release 2015 data.

To assess which SNPs might have violated the key MR assumption regarding pleiotropy, we performed goodness-of-fit tests to correct both GRSs for the heterogeneity of their corresponding SNPs’ effects on each outcome. Heterogeneity, which indicates the potential presence of pleiotropy, was measured using the *Q* statistic and was considered statistically significant at a conservative uncorrected *p-*value of <0.05. Although heterogeneity could be an indicator of pleiotropy, there are other factors that could introduce heterogeneity in the analyses. Even though the adjustments for heterogeneity that we have made could be overconservative, we have used this method in order to minimize false positives. After stepwise removal of SNPs with potential pleiotropic effects, we repeated the analyses until significant heterogeneity was no longer observed.

To further ensure the strength of these two GRSs as IVs, we generated an *F*-statistic for each outcome. We used variance in lnCRP explained by each set of CRP SNPs (2% and 5%, respectively, for GRS_*CRP*_ and GRS_*GWAS*_) to calculate the *F*-statistic using the formula *F*-statistic = [*R* ^2^ × (*n −* 1 − *K*)]/[(1 − *R* ^2^) × *K*], where *R* ^2^ represents the proportion of variability in CRP level that is explained by the GRS, *n* represents sample size, and *K* represents the number of IVs included in model (i.e., for this study *K* = 1) [62]. As a rule of thumb, an *F*-value above ten indicates that a causal estimate is unlikely to be biased due to weak instruments [57].

### Multiple Testing

The present study included 32 independent sample sets. For each sample set, we did one statistical test, for which a global nominal significance level of ≤0.05 was considered as satisfactory to derive conclusions. The need for correction for multiple testing is debatable. Nevertheless, to ensure the validity of our conclusions, we took a conservative approach and applied a Bonferroni-corrected significance threshold calculated as 0.05 divided by 32 (i.e., 0.0016). We considered a statistical test with an observed *p-*value more than 0.05 as a definitely nonsignificant result, i.e., no association; an observed *p-*value equal to or less than 0.05 as nominally significant evidence for a potential, but yet to be confirmed, causal association; and an observed *p-*value equal to or less than 0.0016 as statistically significant evidence for a causal association.

### CRP Polygenic Risk Score and Schizophrenia Using Individual-Level Data

In an ancillary follow-up study, inspired by comments by the editors and the reviewers, we aimed further to determine whether GRS_*GWAS*_ was causally associated with schizophrenia using individual-level data retrieved from the Psychiatric Genomics Consortium (PGC) schizophrenia dataset (S3 Methods) [54]. This dataset consisted of 36 independent cohorts with a combined 25,629 cases and 30,976 controls for which we had ethics approval (S4 Methods). Three family-based samples of European ancestry (1,235 parent–affected offspring trios) were excluded from our analysis. To evaluate whether the observed protective causal association between GRS_*GWAS*_ and schizophrenia was persistent, we investigated whether the CRP_*PRS*_ was also protectively associated with schizophrenia. Briefly, CRP_*PRS*_ values were calculated for each individual by summing the total effect of the SNP dosages by their effect size. In addition to the 18 genome-wide significant CRP SNPs, we grouped subthreshold CRP-associated SNPs at the following *p*-value thresholds: 1 × 10^−4^, 0.001, 0.01, 0.05, and 0.1. Standardized CRP_*PRS*_ values were tested for association with schizophrenia case status in each cohort with adjustment for ten principal components (PCs). A fixed effects inverse-variance-weighted meta-analysis was performed across all 36 cohorts to obtain the overall effect size estimate as explained in S4 Methods and elsewhere [63]. The variance in schizophrenia case status explained by CRP_*PRS*_ was estimated using the deviation in Nagelkerke’s pseudo-*R* ^2^ between a null model (which included ten PCs) and the full model (which included GRS in addition to the ten PCs), calculated in R using the Functions for Medical Statistics Book with Some Demographic Data (fmsb) R package (S3 Methods). Similar to previous studies, the statistical significance of CRP_*PRS*_ values was estimated based on their logistic regression coefficient [64], and reported CRP_*PRS*_ ORs correspond to a 1-SD increase in CRP_*PRS*_ [65].

## Results

Using GRS_*CRP*_, we first tested whether a *CRP*-gene-determined increase in lnCRP was associated with each outcome. In Table 2, the causal effects of lnCRP estimated for each outcome are summarized. We found no heterogeneity in the IV analyses (*p*_heterogeneity_ ≥ 0.11 for all outcomes), and GRS_*CRP*_ was a strong instrument (*F* ≥ 31). IV analyses provided nominal evidence for potential causal relationships of lnCRP with risk of Crohn disease (OR 0.78 [95% CI 0.65–0.94]; *p <* 0.009), psoriatic arthritis (1.45 [1.04–2.04]; *p <* 0.03), and schizophrenia (0.90 [0.82–0.99]; *p <* 0.03), and with an increase in systolic blood pressure (SBP) (mean increase 1.23 mm Hg per 10-s% increase in CRP level [95% CI 0.45–2.01]; *p <* 0.002) and diastolic blood pressure (DBP) (0.70 [0.20–1.19]; *p <* 0.006). GRS_*CRP*_ showed no significant effect on any of the other outcomes (Table 2; S1 Fig).

**Table 2:** The effect of the CRP genetic risk score instrument of four SNPs in CRP (GRS_*CRP*_) with somatic and neuropsychiatric outcomes.

Disease or Trait	*M*	*N*	Effect Size (95% CI)^1^	Goodness-of-Fit Test *p*-Value	*p*-Het	*F*-Value
**Autoimmune/inflammatory**						
Celiac disease	3	15,283	0.96 (0.77 to 1.21)	0.750	0.19	311.86
IBD (all types)	3	47,794	0.97 (0.84 to 1.13)	0.700	0.30	975.35
Crohn disease	4	21,389	0.78 (0.65 to 0.94)	0.009	0.25	436.47
Ulcerative colitis	4	26,405	1.10 (0.92 to 1.31)	0.290	0.92	538.84
Psoriasis vulgaris	4	8,941	1.23 (0.96 to 1.57)	0.110	0.95	182.43
Psoriatic arthritis	4	6,880	1.45 (1.04 to 2.04)	0.030	0.92	140.37
Cutaneous psoriasis	4	4,880	1.10 (0.76 to 1.59)	0.620	0.60	99.55
Rheumatoid arthritis	4	25,702	0.94 (0.77 to 1.15)	0.550	0.17	524.55
Systemic lupus erythematous	3	4,651	1.20 (0.80 to 1.81)	0.380	0.19	94.88
Systemic sclerosis	3	7,518	1.07 (0.78 to 1.45)	0.680	0.85	153.90
Type 1 diabetes	2	26,890	1.15 (0.90 to 1.47)	0.260	0.34	548.73
Knee osteoarthritis	4	24,260	0.94 (0.78 to 1.13)	0.500	0.23	495.06
**Cardiovascular**						
CAD	4	184,305	1.00 (0.93 to 1.07)	0.965	0.65	1,775.37
SBP^2^	4	69,372	1.23 (0.45 to 2.01)	0.002	0.51	1,415.63
DBP^2^	4	69,368	0.70 (0.2x to 1.19)	0.006	0.68	1,415.71
Ischemic stroke (all types)	4	9,520	1.19 (0.93 to 1.53)	0.160	0.93	194.24
Ischemic stroke (cardioembolic)	4	6,762	1.02 (0.65 to 1.58)	0.940	0.96	137.96
Ischemic stroke (large vessel)	4	6,816	1.44 (0.93 to 2.21)	0.100	0.31	139.06
Ischemic stroke (small vessel)	4	6,552	1.18 (0.71 to 1.95)	0.520	0.36	133.06
**Metabolic**						
Body mass index^3^	4	123,864	−0.017 (−0.06 to 0.02)	0.410	0.50	2,527.82
Type 2 diabetes	4	22,570	1.11 (0.94 to 1.32)	0.230	0.50	460.57
Chronic kidney disease	4	74,354	1.04 (0.88 to 1.22)	0.670	0.90	1,517.39
eGFR_**cr**_ ^4^	4	74,354	0.004 (−0.01 to 0.02)	0.400	0.88	1,517.39
Serum albumin level^5^	4	53,189	−0.002 (−0.02 to 0.01)	0.770	0.88	1,085.45
Serum protein level^5^	4	25,537	0.008 (−0.02 to 0.04)	0.640	0.12	521.12
**Neurodegenerative**						
Amyotrophic lateral sclerosis	2	12,263	0.79 (0.60 to 1.04)	0.090	0.23	258.39
Alzheimer disease	2	13,020	1.26 (0.89 to 1.78)	0.200	0.11	265.67
Parkinson disease	3	17,352	1.00 (0.85 to 1.17)	0.960	0.33	354.08
**Psychiatric**						
Autism	3	1,566	1.02 (0.97 to 1.07)	0.380	0.69	31.92
Bipolar disorder	4	16,731	1.17 (0.97 to 1.42)	0.110	0.49	341.41
Major depressive disorder	3	18,759	0.98 (0.81 to 1.18)	0.810	0.86	382.80
Schizophrenia	3	79,845	0.90 (0.82 to 0.99)	0.030	0.79	1,629.45

^1^Effect size (95% CI) per 1-mg/l increase in lnCRP. For risk of disease, effect size is given as an OR, otherwise given in the specific units in which the outcome was measured. Derived from the IV causal estimator α.

^2^Effect size unit is millimeters of mercury per 1-mg/l increase in lnCRP.

^3^Effect size unit is standard deviations per 1-mg/l increase in lnCRP (the body mass index results were inverse normal transformed to a distribution with μ = 0 and σ = 1).

^4^Effect size unit is milliliters/minute/1.73 m^2^ per 1-mg/l increase in lnCRP.

^5^Effect size unit is grams/deciliter per 1-mg/l increase in lnCRP.

eGFR_cr_, estimated glomerular filtration rate from serum creatinine; *F-*value, *F*-statistic value for the genetic instrument; *M*, number of markers used in the genetic instrument; *N*, number of samples in the disease/trait meta-analysis; *p-*het, *p-*value of heterogeneity of effect test.

**Fig 1:**
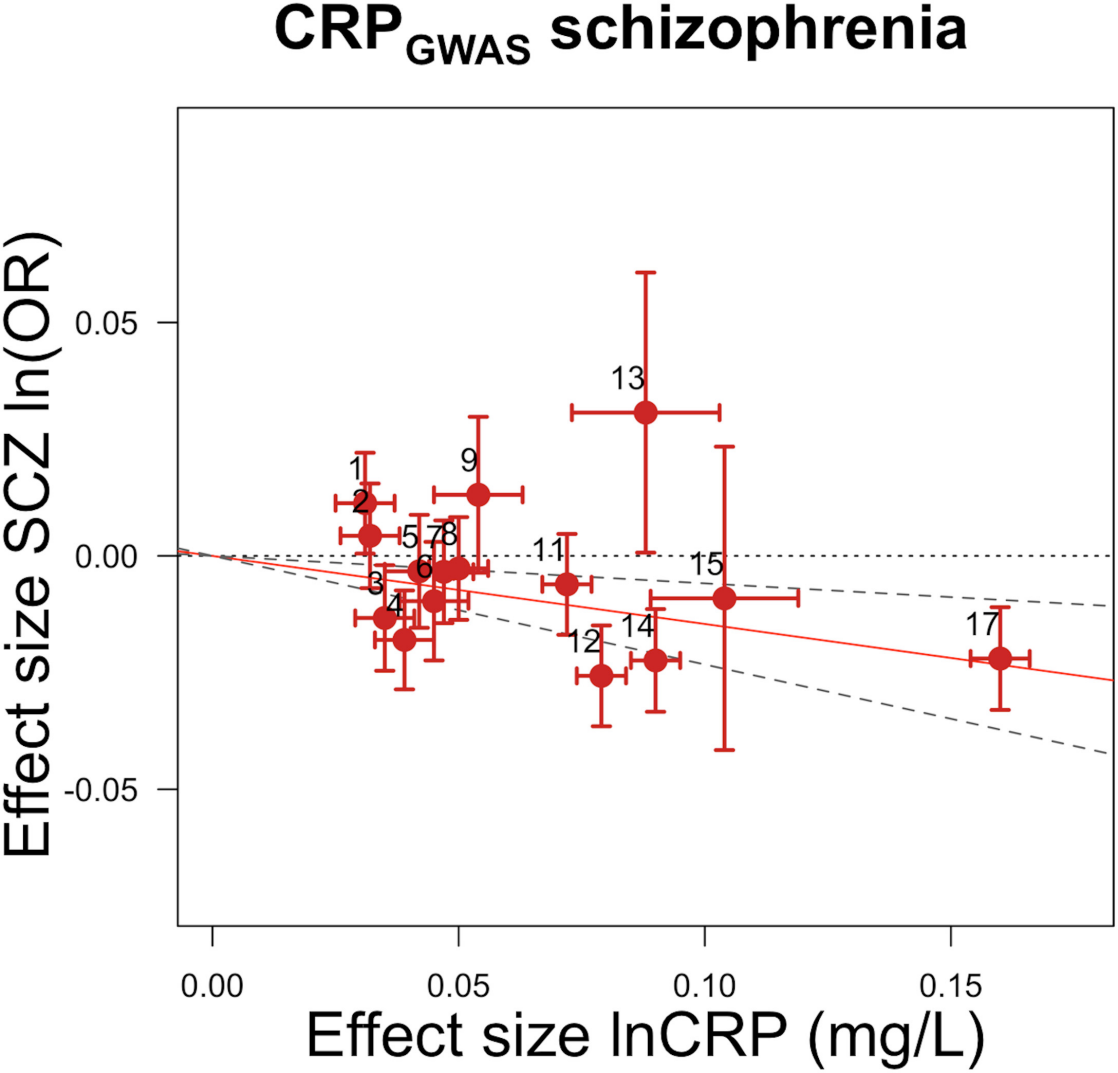
Genetic risk score GRS_*GWAS*_ for schizophrenia. The *x-*axis shows the effect size for the 15 SNPs for which data were available in the PGC schizophrenia dataset comprising the GRS_*GWAS*_ influencing levels of CRP, with corresponding standard error bars. The *y-*axis shows the log OR of the GRS_*GWAS*_ SNPs for schizophrenia (SCZ) with corresponding standard error bars. The effect estimate of CRP level on disease risk is represented by the red solid line, with gradient α. The 95% CI of this α estimate is represented by the grey dashed lines. The included SNPs are shown by Arabic numbering: #1, rs2847281 (gene: *PTPN2*; chromosome: 18; basepair position: 12811593); #2, rs340029 (*RORA*; 15; 58682257); #3, rs6901250 (*GPRC6A*; 6; 117220718); #4, rs10745954 (*ASCL1*; 12; 102007224); #5, rs4705952 (*IRF1*; 5; 131867517); #6, rs12037222 (*PABPC4*; 1; 39837548); #7, rs12239046 (*NLRP3*; 1; 245668218); #8, rs6734238 (*IL1F10*; 2; 113557501); #9, rs13233571 (*BCL7B*; 7; 72609167); #11, rs1260326 (*GCKR*; 2; 27584444); #12, rs4129267 (*IL6R*; 1; 152692888); #13, rs1800961 (*HNF4A*; 20; 42475778); #14, rs4420065 (*LEPR*; 1; 5934049); #15, rs10521222 (*SALL1*; 16; 49716211); 12; 119905190); #17, rs2794520 (*CRP*; 1; 157945440). The three SNPs of #10, rs9987289 (*PPP1R3B*; 8; 9220768); #16, rs1183910 (*HNF1A*; and #18, rs4420638 (*APOC1*; 19; 50114786) were not present in the data of the PGC.

**Fig 2:**
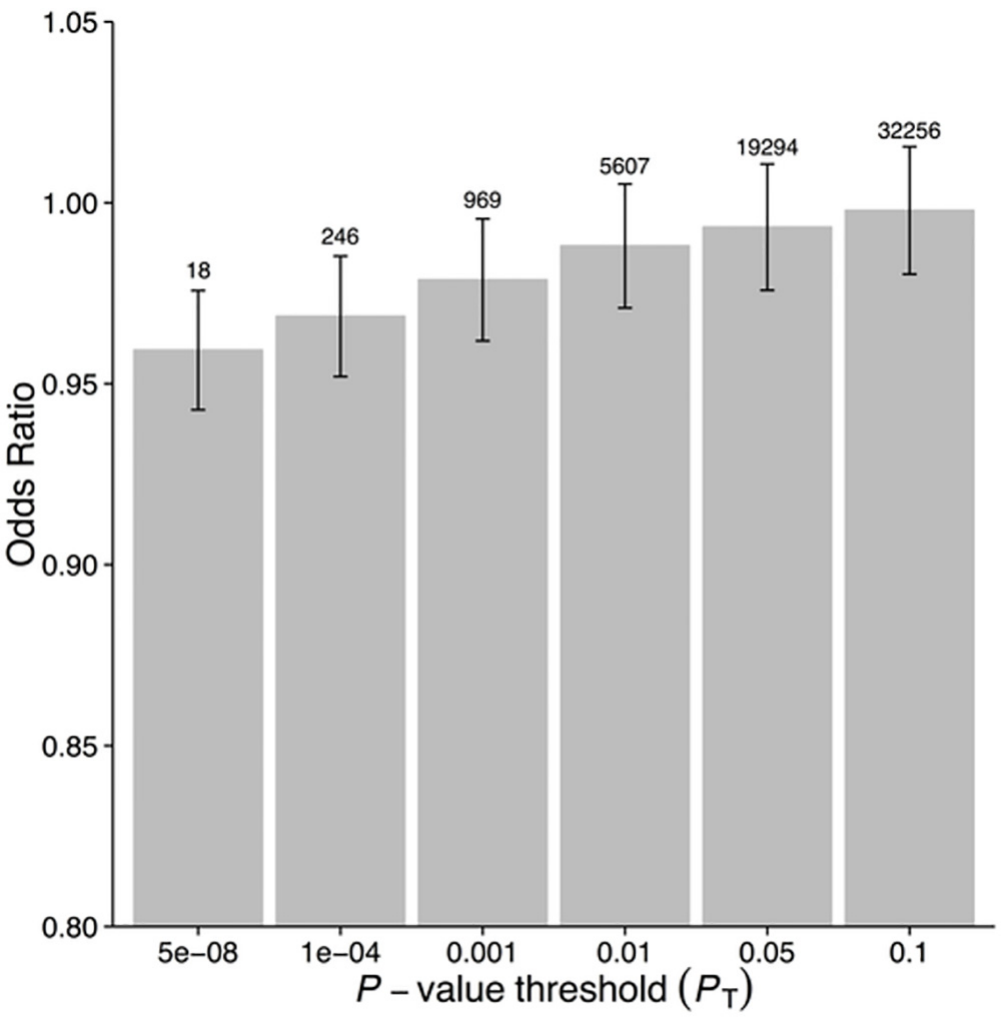
Polygenic risk scores for elevated CRP level and protective effect on schizophrenia, using individual-level genetic data.

**Fig 3:**
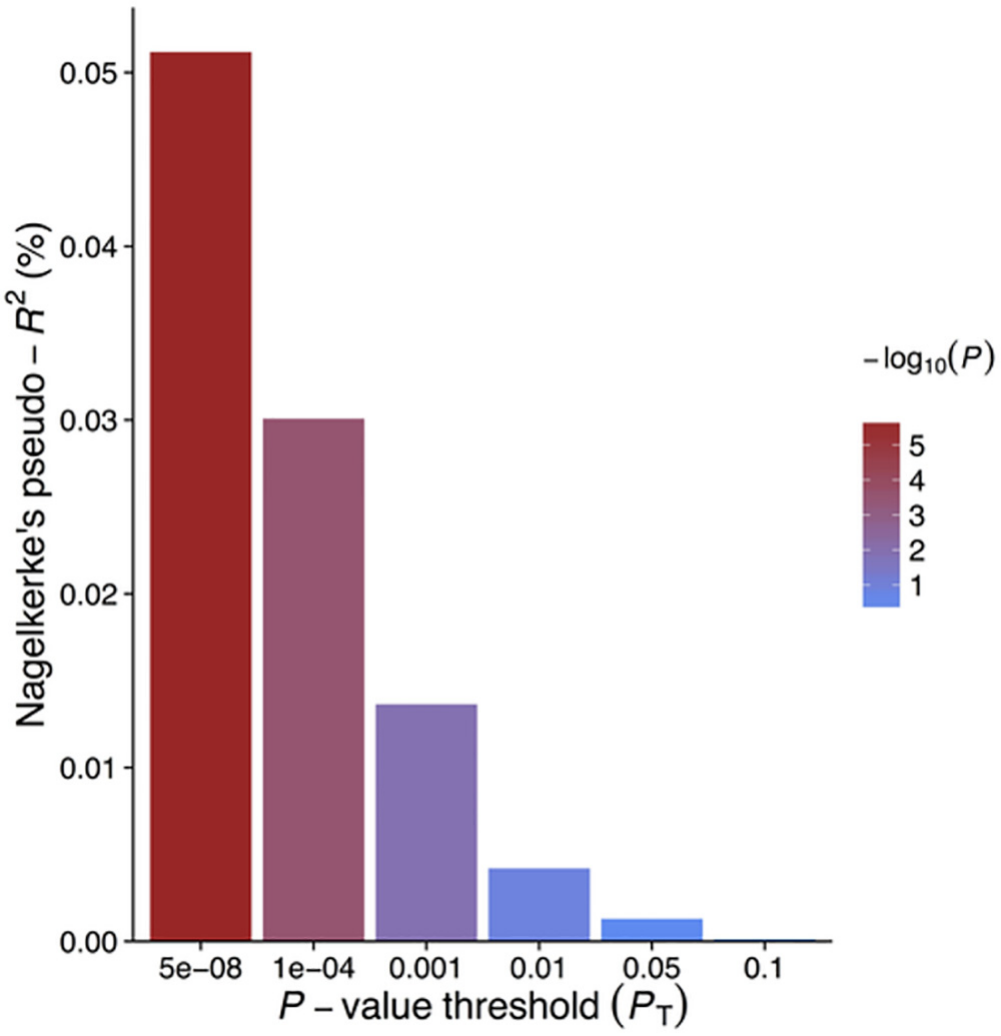
Polygenic risk scores for elevated CRP level and explained variance of schizophrenia using individual-level genetic data.

GRS_*GWAS*_ showed a statistically significant protective effect of lnCRP on the risk of schizophrenia (per 10-s% increase in CRP level, OR 0.86 [95% CI 0.79–0.94]; *p <* 0.0010) (Figs 1 and S1; Table 3). In a follow-up analysis using the individual-level PGC data, we found that a GRS incorporating the same 18 CRP SNPs used to construct the GRS_*GWAS*_ was again significantly associated with a lower risk of schizophrenia (OR 0.96 [95% CI 0.94–0.98]; *p <* 1.72 × 10^−6^). This signal persisted when we included all SNPs meeting a less stringent *p-*value threshold of 1 × 10^−4^ (OR 0.97 [95% CI 0.95–0.99]; *p <* 2.45 × 10^−4^). At less stringent *p-*value thresholds, less variance was explained by the logistic model, and the protective effect of CRP risk scores became less significant, but across all *p-*value thresholds, the direction of the effect was consistently protective (Figs 2 and 3). To ensure that the association between risk alleles for CRP and schizophrenia was not driven by a small number of genome-wide significant SNPs, we performed a leave-one-out sensitivity analysis of the 18 genome-wide SNPs. In the 18 sets of 17 SNPs, the variance explained (Nagelkerke’s pseudo-*R* ^2^) ranged from 0.012% to 0.034%, with *p-*values ranging from 9.3 × 10^−5^ to 1.6 × 10^−2^, suggesting that the protective effect observed between risk alleles for CRP and schizophrenia was not driven by a small number of SNPs with large effects.

**Table 3:** The effect of the CRP genetic risk score instrument of 18 SNPs associated with CRP (GRS_*GWAS*_) on somatic and neuropsychiatric outcomes.

Disease or Trait	*M*	Effect Size (95% CI)^1^	Goodness-of-Fit Test *p*-Value	*p*-Het	*F*-Value
**Autoimmune/inflammatory**					
Celiac disease	18	0.99 (0.85 to 1.16)	0.930	7.2 × 10^−4^	804.26
IBD (all types)	15	0.85 (0.74 to 0.98)	0.030	1.4 × 10^−5^	2,515.37
Crohn disease	17	0.81 (0.70 to 0.94)	0.005	4.4 × 10^−7^	1,125.63
Ulcerative colitis	17	1.05 (0.91 to 1.21)	0.490	0.01	1,389.63
Psoriasis vulgaris	17	1.12 (0.90 to 1.40)	0.310	0.19	470.47
Psoriatic arthritis	17	1.36 (1.00 to 1.84)	0.049	0.04	362.00
Cutaneous psoriasis	17	1.00 (0.72 to 1.39)	0.990	0.16	256.74
Rheumatoid arthritis	18	0.93 (0.80 to 1.08)	0.350	1.8 × 10^−6^	1,352.79
Systemic lupus erythematous	11	1.06 (0.71 to 1.58)	0.780	0.27	244.68
Systemic sclerosis	11	0.84 (0.62 to 1.14)	0.280	0.63	396.89
Type 1 diabetes	15	1.10 (0.92 to 1.31)	0.310	3.47 × 10^−3^	1,415.16
Knee osteoarthritis	18	1.17 (1.01 to 1.36)	0.040	0.10	1,276.74
**Cardiovascular**					
CAD	18	0.88 (0.84 to 0.94)	2.4 × 10^−5^	7.5 × 10^−12^	9,403.21
SBP^2^	18	0.72 (0.11 to 1.34)	0.020	0.14	3,650.84
DBP^2^	18	0.45 (0.06 to 0.84)	0.020	0.02	3,651.05
Ischemic stroke (all types)	18	1.06 (0.87 to 1.29)	0.570	0.37	500.95
Ischemic stroke (cardioembolic)	18	0.98 (0.69 to 1.39)	0.920	0.35	355.79
Ischemic stroke (large vessel)	18	1.30 (0.92 to 1.82)	0.140	0.97	358.63
Ischemic stroke (small vessel)	18	0.85 (0.58 to 1.25)	0.420	0.76	343.16
**Metabolic**					
Body mass index^3^	18	−0.005 (−0.03 to 0.02)	0.740	0.11	6,519.11
Type 2 diabetes	18	1.090 (0.95 to 1.24)	0.210	1.8 × 10^−3^	1,187.79
Chronic kidney disease	18	0.960 (0.84 to 1.09)	0.500	0.07	3,913.26
eGFR_**cr**_ ^4^	18	0.011 (0.003 to 0.02)	0.005	7.2 × 10^−9^	3,913.26
Serum albumin level^5^	18	0.011 (0.0004 to 0.02)	0.041	2.3 × 10^−18^	2,799.32
Serum protein level^5^	18	0.031 (0.008 to 0.05)	0.009	0.03	1,343.95
**Neurodegenerative**					
Amyotrophic lateral sclerosis	8	1.01 (0.79 to 1.29)	0.960	0.56	666.37
Alzheimer disease	11	1.26 (0.99 to 1.61)	0.060	0.23	685.16
Parkinson disease	10	1.06 (0.90 to 1.25)	0.500	0.50	913.16
**Psychiatric**					
Autism	9	0.89 (0.70 to 1.13)	0.350	0.99	82.32
Bipolar disorder	18	1.21 (1.05 to 1.40)	0.007	0.15	880.47
Major depressive disorder	15	1.14 (0.96 to 1.36)	0.140	0.84	987.21
Schizophrenia	15	0.86 (0.79 to 0.94)	0.001	0.66	4,202.26

^1^Effect size (95% CI) per 1-mg/l increase in lnCRP. For risk of disease, effect size is given as an OR, otherwise given in the specific units in which the outcome was measured. Derived from the IV causal estimator α.

^2^Effect size unit is millimeters of mercury per 1-mg/l increase in lnCRP.

^3^Effect size unit is standard deviations per 1-mg/l increase in lnCRP (the body mass index results were inverse normal transformed to a distribution with μ = 0 and σ = 1).

^4^Effect size unit is milliliters/minute/1.73 m^2^ per 1-mg/l increase in lnCRP.

^5^Effect size unit is grams/deciliter per 1-mg/l increase in lnCRP.

eGFR_cr_, estimated glomerular filtration rate from serum creatinine; *F-*value, *F*-statistic value for the genetic instrument; *M*, number of markers used in the genetic instrument; *p-*het, *p-*value of heterogeneity of effect test.

**Fig 4:**
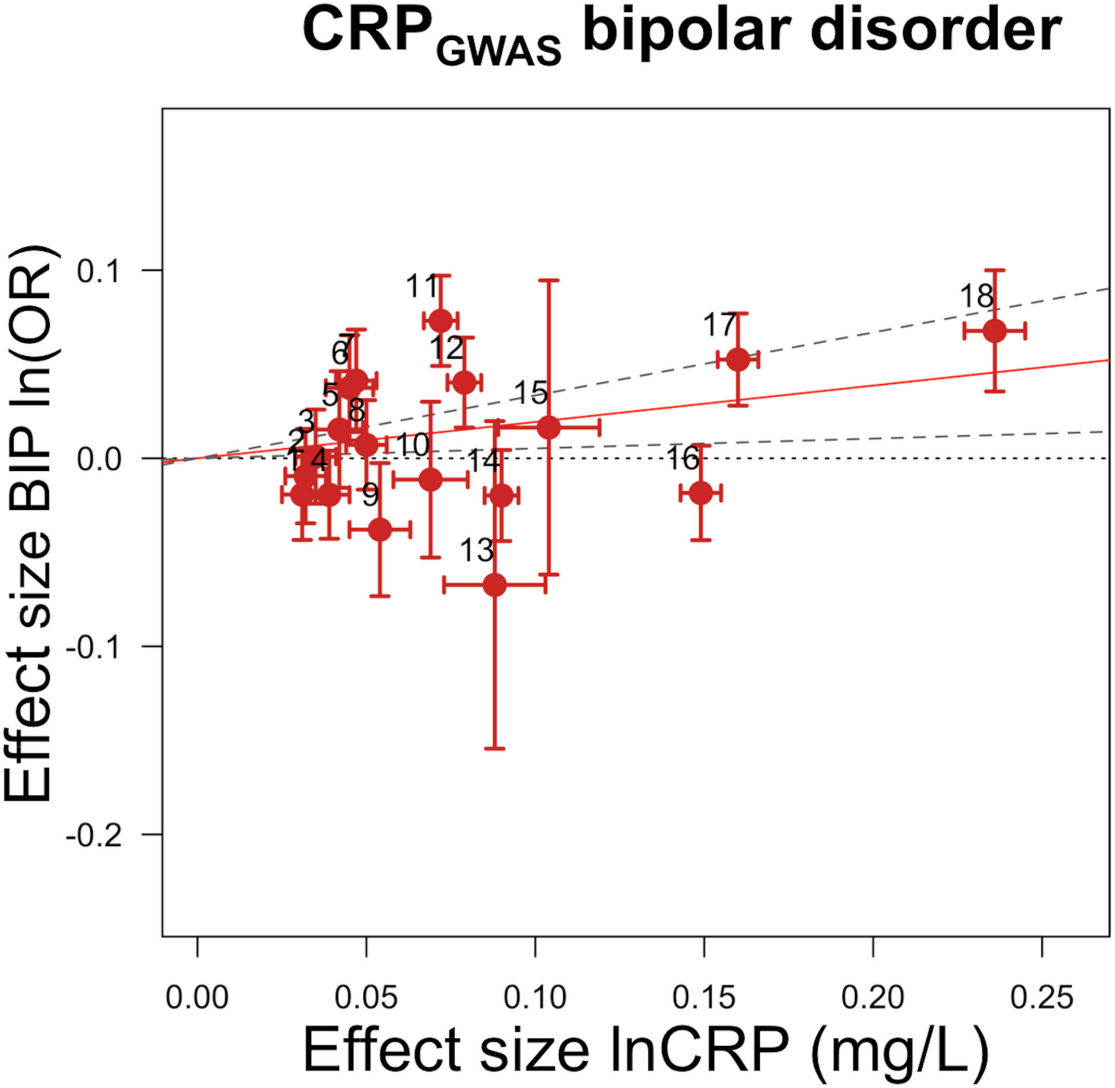
Genetic risk score GRS_*GWAS*_ for bipolar disorder. The *x-*axis shows the effect size for the 18 SNPs comprising the GRS_*GWAS*_ influencing levels of CRP, with corresponding standard error bars. The *y-*axis shows the log OR of the GRS_*GWAS*_ SNPs for bipolar disorder (BIP) with corresponding standard error bars. The effect estimate of CRP level on disease risk is represented by the red solid line, with gradient α. The 95% CI of this α estimate is represented by the grey dashed lines. The included SNPs are shown by Arabic numbering: #1, rs2847281 (gene: *PTPN2*; chromosome: 18; basepair position: 12811593); #2, rs340029 (*RORA*; 15; 58682257); #3, rs6901250 (*GPRC6A*; 6; 117220718); #4, rs10745954 (*ASCL1*; 12; 102007224); #5, rs4705952(*IRF1*; 5; 131867517); #6, rs12037222 (*PABPC4*; 1; 39837548); #7, rs12239046 (*NLRP3*; 1; 245668218); #8, rs6734238 (*IL1F10*; 2; 113557501); #9, rs13233571 (*BCL7B*; 7; 72609167); #10, rs9987289 (*PPP1R3B*; 8; 9220768); #11, rs1260326 (*GCKR*; 2; 27584444); #12, rs4129267 (*IL6R*; 1; 152692888); #13, rs1800961 (*HNF4A*; 20; 42475778); #14, rs4420065 (*LEPR*; 1; 5934049); #15, rs10521222 (*SALL1*; 16; 49716211); #16, rs1183910 (*HNF1A*; 12; 119905190); #17, rs2794520 (*CRP*; 1; 157945440); #18, rs4420638 (*APOC1*; 19; 50114786).

GRS_*GWAS*_ also showed moderate but nominally significant effects of lnCRP on the risk of IBD (OR 0.85 [95% CI 0.74–0.98]; *p <* 0.03), Crohn disease (0.81 [0.70–0.94]; *p <* 0.005), psoriatic arthritis (1.36 [1.00–1.84]; *p <* 0.049), knee osteoarthritis (1.17 [1.01–1.36]; *p <* 0.04), and bipolar disorder (1.21 [1.05–1.40]; *p <* 0.007), while its effect was statistically significant for CAD (0.88 [0.84–0.94]; *p <* 2.4 × 10^−5^) (Table 3; Figs 4 and S1). GRS_*GWAS*_ revealed a nominally significant effect of lnCRP on blood pressure: an increase of 0.72 (95% CI 0.11–1.34; *p <* 0.02) and 0.45 (0.06–0.84; *p <* 0.02) mm Hg in SBP and DBP, respectively (Table 3; S1 Fig). Likewise, a genetically determined 10-s% increase in CRP level was nominally associated with a 0.01 ml/min/1.73 m^2^ (95% CI 0.003–0.02; *p <* 0.005) higher estimated glomerular filtration rate from serum creatinine (eGFR_cr_), a 0.01 g/dl (0.0004–0.02; *p <* 0.04) higher serum albumin level, and a 0.03 g/dl (0.008–0.05; *p <* 0.009) higher serum protein level. The remaining outcomes tested for causal associations using GRS_*GWAS*_ did not reach statistical significance, though the corresponding GRS_*GWAS*_ proved to be a strong IV, with *F*-values ≥ 82 (Table 3; S1 Fig).

Using GRS_*GWAS*_, there was no significant evidence of heterogeneity of the effect size for knee osteoarthritis, bipolar disorder, schizophrenia, or SBP, while the heterogeneity test was statistically significant for psoriatic arthritis, IBD, Crohn disease, CAD, DBP, eGFR_cr_, serum albumin, and serum protein. These heterogeneities in the effects of GRS_*GWAS*_ may be attributable to pleiotropic effects of the SNPs used to build the GRS_*GWAS*_. We subsequently performed a stepwise removal of SNPs from GRS_*GWAS*_ until no significant heterogeneity remained (Table 4). This adjustment in the GRS_*GWAS*_ resulted in the removal of three SNPs from the GRS_*GWAS*_ for IBD (in *GCKR*, *IRF1*, and *PTPN2*), five SNPs from the GRS_*GWAS*_ for Crohn disease (in *GCKR*, *IL6R*, *IRF1*, *PABPC4*, and *PTPN2*), one SNP from the GRS_*GWAS*_ for psoriatic arthritis (in *IRF1*), three SNPs for CAD (in *APOC1*, *HNF1A*, and *IL6R*), one SNP from the GRS_*GWAS*_ for DBP (in *PABPC4*), two SNPs from the GRS_*GWAS*_ for eGFR_cr_ (in *LEPR* and *GCKR*), six SNPs from the GRS_*GWAS*_ for serum albumin level (in *APOC1*, *BCL7B*, *GCKR*, *PPP1R3B*, *PTPN2*, and *IRF1*), and one SNP from the GRS_*GWAS*_ for serum protein level (in *GCKR*). After removal of these variants from the GRS_*GWAS*_, we found no statistically significant (at *p <* 0.0016) association between genetically increased lnCRP level and any of these outcomes (Table 4). However, the effect estimate of CRP on DBP, serum albumin, and psoriatic arthritis showed nominal association at *p <* 0.05. For example, for DBP, 17 SNPs remained in the GRS_*GWAS*_ and yielded a slightly lower causal estimate (compared to the values before adjustment) of a 0.39 (95% CI −0.01 to 0.78) mm Hg increase in DBP per 10-s% increase in lnCRP level, with a nominal significance of *p <* 0.05.

**Table 4:** The effect of the CRP genetic risk score instrument of 18 SNPs associated with CRP (GRS_*GWAS*_) on somatic and neuropsychiatric outcomes after correcting for heterogeneity.

Disease or Trait	*M*	Effect Size (95% CI)^1^	Goodness-of-Fit Test *p*-Value	*p*-Het
**Autoimmune/inflammatory**				
Celiac disease	16	1.05 (0.90 to 1.23)	0.56	0.10
IBD	12	0.92 (0.79 to 1.06)	0.24	0.14
Crohn disease	12	0.93 (0.79 to 1.08)	0.34	0.12
Ulcerative colitis	16	1.11 (0.96 to 1.28)	0.16	0.12
Psoriatic arthritis	16	1.42 (1.05 to 1.94)	0.02	0.14
Rheumatoid arthritis	13	0.83 (0.71 to 0.97)	0.02	0.09
Type 1 diabetes	14	1.06 (0.89 to 1.27)	0.52	0.07
**Cardiovascular**				
CAD	15	0.98 (0.91 to 1.06)	0.65	0.20
DBP^2^	17	0.385 (0.008 to 0.78)	0.05	0.09
**Metabolic**				
Type 2 diabetes	17	0.95 (0.82 to 1.10)	0.52	0.09
eGFR_cr_ ^3^	16	0.001 (−0.007 to 0.01)	0.74	0.11
Serum albumin level^4^	12	−0.017 (−0.03 to −0.004)	0.01	0.07
Serum protein level^4^	17	0.021 (−0.002 to 0.05)	0.07	0.31

^1^Effect size (95% CI) per 1-mg/l increase in lnCRP. For risk of disease, effect size is given as an OR, otherwise given in the specific units in which the outcome was measured. Derived from the IV causal estimator α.

^2^Effect size unit is millimeters of mercury per 1-mg/l increase in lnCRP.

^3^Effect size unit is milliliters/minute/1.73 m^2^ per 1-mg/l increase in lnCRP.

^4^Effect size unit is grams/deciliter per 1-mg/l increase in lnCRP.

*M*, number of markers used in the genetic instrument; *p-*het, *p-*value of heterogeneity of effect test.

Likewise, we hypothesized that the fact that GRS_*GWAS*_ showed a nonsignificant effect of CRP on celiac disease, ulcerative colitis, rheumatoid arthritis, type 1 diabetes, and type 2 diabetes can be to some extent explained by the significant heterogeneity observed for these outcomes (Table 3). The stepwise adjustment in the GRS_*GWAS*_ resulted in the removal of two SNPs from the GRS_*GWAS*_ for celiac disease (in *PABPC4* and *PTPN2*), one SNP from the GRS_*GWAS*_ for ulcerative colitis (in *GCKR*), five SNPS from the GRS_*GWAS*_ for rheumatoid arthritis (in *HNF4A*, *IL6R*, S*ALL1*, *NLRP3*, and *PTPN2)*, one SNP from the GRS_*GWAS*_ for type 1 diabetes (in *PTPN2*), and one SNP from the GRS_*GWAS*_ for type 2 diabetes (in *APOC1*). After adjusting for heterogeneity, the association of GRS_*GWAS*_ with these outcomes remained statistically nonsignificant (Table 4).

## Discussion

In this large-scale cross-consortium MR study of 32 complex outcomes, we found evidence for a potential protective causal relationship between elevated CRP level and schizophrenia in both genetic IVs (i.e., GRS_*CRP*_ and GRS_*GWAS*_) and confirmed this protective relationship in follow-up analyses using individual-level genotype data from the schizophrenia GWAS. We also found a statistically significant association of CRP level with CAD, and nominally significant evidence for a predisposing causal association of CRP level with IBD, Crohn disease, psoriatic arthritis, knee osteoarthritis, SBP, DBP, eGFR_cr_, serum albumin level, serum protein level, and bipolar disorder, using GRS_*GWAS*_ as an IV. However, after adjustment for heterogeneity, neither GRS showed a significant effect (at *p* < 0.0016) of CRP level on any of these outcomes, including CAD, nor on the 20 other common somatic and psychiatric outcomes we investigated, including celiac disease, ulcerative colitis, psoriasis (all types), rheumatoid arthritis, systemic lupus erythematous, systemic sclerosis, type 1 and 2 diabetes, stroke (all types), body mass index, chronic kidney disease, amyotrophic lateral sclerosis, Alzheimer disease, Parkinson disease, autism, and major depressive disorder.

### CRP Protection against Schizophrenia

Strikingly, as opposed to the current literature and previous inconclusive small-scale studies [66–68], our findings suggest that genetically elevated levels of CRP are not predisposing but in fact protective for schizophrenia. The significant causal protective role of CRP for schizophrenia was consistent in both IVs using summary statistics, i.e., GRS_*CRP*_ and GRS_*GWAS*_. When incorporating 18 genome-wide CRP-associated SNPs using individual-level data, we confirmed a modest, but significant, protective effect of CRP level for schizophrenia. This signal persisted when we included all SNPs meeting a less stringent *p*-value threshold of 1×10^−4^. Notably, the leave-one-out sensitivity analysis revealed that the genetic overlap between CRP level and schizophrenia we observed at genome-wide and 1×10^−4^ significance thresholds was not driven by a few major SNPs. In contrast, others have previously shown that CRP levels are significantly elevated in patients with schizophrenia [69,70], with a recent meta-analysis concluding that the association between elevated CRP and schizophrenia is indeed robust [71]. Given that clinical studies report elevated CRP levels in schizophrenia, one would expect to find that alleles for elevated CRP would confer an increased risk for schizophrenia. The fact that we found the completely opposite effect—in a cohort of over 25,000 cases and 30,000 controls—should give one pause when deriving clinical meaning from these results. Our observation that a genetically determined marginal increase in the level of CRP is likely to be protective for schizophrenia may fuel the debate about whether the observed CRP elevation in schizophrenia is a by-product of the pathogenesis of schizophrenia or directly contributing to clinical manifestations of the disorder [6]. Our finding may also point out potential biases in previous studies regarding the causes of elevated CRP levels in patients with schizophrenia, such as reverse causality and/or pleiotropic effects within chosen instruments.

The exact mechanism for how elevated CRP levels are linked to schizophrenia requires a well-defined experimental analysis. In addition to CRP variants, other recent studies have identified several inflammatory genetic variants associated with schizophrenia and bipolar disorder, which include variants in the major histocompatibility complex (MHC) region on Chromosome 6p21 [72]—harboring many cytokine genes [54,73–76]—and in the *IL10* promoter [77], *TNF* promoter [78], *IL1B* [79], and C4 [80].

### Biological Annotation

Following comments made by the reviewers, we explored the possible underlying pathways that may explain the potential protective causal association between CRP and schizophrenia. We performed a follow-up in silico functional pathway analysis using a previously reported approach [81] as summarized in S5 Methods and S4–S13 Tables. In brief, our results show that pathways associated with the interferon response are significantly enriched amongst genes harbored by CRP loci and their associated expression quantitative trait loci (eQTLs) and that there are differentially expressed genes between schizophrenia cases and controls. Previous studies showed that the induction of T cell IFN cytokine release stimulates microglia and astrocytes to facilitate glutamate clearance in neuronal cells without evoking inflammatory mediators [82,83]. One could speculate that CRP-interferon pathways may induce neuroprotection by contributing to glutamate clearance, leading to the protection of neurons against the oxidative stress associated with an excess of glutamate [84,85], and thereby offering a protective effect against schizophrenia.

### CRP GRS_*GWAS*_ Association with Bipolar Disorder

As for bipolar disorder, we found a nominal effect of a 1.21-fold increase in risk for bipolar disorder with a 10-s% increase in CRP level. Though this nominal predisposing effect needs to be confirmed, our finding corroborates epidemiological observations suggesting that elevated CRP is associated with the disease and supports a potential causal influence of general inflammation in bipolar disorder [86]. We note that, though it may be biologically sensible, this result failed to pass multiple testing correction. Confirmation by replication in independent cohorts, functional follow-up analyses, or the use of a stronger CRP GRS_*GWAS*_ in upcoming studies is required to draw a definitive conclusion.

### CRP GRS_*GWAS*_ Association with Blood Pressure and Hypertension

We found nominally significant evidence for an up to ~0.70-mm Hg increase in blood pressure with a 10-s% increase in CRP level and no evidence of heterogeneity for SBP. Additionally, there was nominally borderline significance for a causal association between CRP and DBP after adjustment for heterogeneity. These nominally significant findings, on the one hand, are in line with numerous epidemiological studies that have highlighted an association between elevated CRP and an increased risk of hypertension. For instance, one study found an association between CRP loci and hypertension in Asian individuals [87]. An additional line of support for a possible causal association of CRP and blood pressure comes from an experimental study in which an increase in *CRP* gene expression in mice, and subsequently CRP protein levels, led to a rise in SBP particularly [88]. Moreover, an ex vivo study by Zhou et al. showed that combining IL6 treatment and mechanical strain leads to a consistent increase in CRP expression at the protein and mRNA levels in smooth muscle cells [89]. Both inflammatory factors and local mechanical strains are abundant in blood vessels and are well-known risk factors for high blood pressure. Our finding did not reach a statistically significant level after correction for multiple testing; thus, it may echo previous MR studies that have failed to find a causal relationship between CRP level and blood pressure or hypertension in Europeans [90,91]. However, our systematic literature review showed that previous studies had some limitations (S1 Table). For instance, no study used a refined GWAS set of 18 CRP-associated SNPs; instead, they tested single or a limited set of CRP SNPs. Using such instruments might have led to biased estimates as their corresponding effects on CRP levels have been found to be small [30,57]. A combination of weak instruments and small sample sizes might have led to type II error [28,57] and hence to a conclusion of no causal association between CRP and blood pressure traits in previous studies. When all of the evidence is taken together, a direct link between CRP and blood pressure remains to be elucidated, though our nominal associations between GRS_*CRP*_ and GRS_*GWAS*_ and blood pressure do add to a line of findings from experimental studies suggesting a potential causal relationship between CRP and blood pressure.

### CRP GRS_*GWAS*_ Association with Osteoarthritis

Our nominally significant finding that CRP might be a potential causal factor for knee osteoarthritis (using GRS_*GWAS*_) should be interpreted with caution. In line with our findings, we have previously shown that levels of CRP were higher in women with early radiological knee osteoarthritis (i.e., Kellgren-Lawrence grade 2+) and in women whose disease progressed [92]. Additionally, another study showed that genetically elevated CRP levels contribute to osteoarthritis severity [93]. However, other studies have found contrasting results [71,72,94]. One systematic review provided evidence that the relationship between CRP and osteoarthritis does exist but is dependent on body mass index [95]. It remains to be further investigated whether weight gain over the lifetime mediates the potential causal association between genetically elevated CRP and knee osteoarthritis.

### CRP GRS_*GWAS*_ Shows No Association with Other Remaining Outcomes

The present study was able to calculate nominal causal estimates for IBD, Crohn disease, psoriatic arthritis, CAD, eGFR_cr_, serum albumin level, and serum protein level using CRP GRS_*GWAS*_, but the estimates were altered by removal of SNPs from GRS_*GWAS*_ based on heterogeneity tests, resulting in nominal or nonsignificant associations. These outcomes appeared therefore to have heterogeneity in the causal estimates, suggesting that these observed estimates were biased, likely due to pleiotropic effects of CRP loci. These results corroborate negative findings of previous studies (S1 Table), suggesting that a causal role of CRP in these traits and diseases is unlikely.

### Methodological Concerns and Advantages

#### Pleiotropic biases in Mendelian randomization analyses using CRP GRS_*GWAS*_

A detailed evaluation of pleiotropic SNPs in our study showed that the method applied to identify heterogeneity sources was able to indicate and exclude several already known pleiotropic loci from the GRS_*GWAS*_ IV. For instance, the use of a SNP in *IL6R* (rs4129267), amongst others, resulted in heterogeneity of effects on CAD risk. The same variant contributed to heterogeneity of effects for Crohn disease in our study, and it has been shown that this SNP is associated with levels of biomarkers other than CRP [56]. Further, a MR study found that *IL6R* SNPs, specifically the nonsynonymous SNP rs8192284, are associated with CAD risk and CRP levels [96]. Our selected *IL6R* SNPs, namely rs4537545 and rs4129267, are in extremely high linkage disequilibrium with rs8192284 (*r* ^2^ ≥ 0.96 for both SNPs in HapMap data, CEU population). Carriers of the risk allele of rs8192284 have higher CRP, IL6, and fibrinogen levels [96]. Fibrinogen is also a well-known risk factor for CAD. Therefore, it is unclear so far which biomarker(s) mediates the effect of *IL6R* SNPs on CAD. Besides the *IL6* locus, *APOC1* and *PABPC4* have been indicated as pleiotropic in three out of 32 our investigated outcomes, and *PTPN2* and *GCKR* in six. With this information taken together, we were able to disentangle at least part of the pleiotropy regarding the causal estimates of CRP for outcomes. Again, we found no significant association of CRP GRS_*GWAS*_ with IBD, Crohn disease, psoriatic arthritis, CAD, eGFR_cr_, serum albumin level, and serum protein level after adjustment for heterogeneity.

#### Using summary statistics of large-scale consortia

It is of utmost interest whether the observed effect of CRP as a risk predictor for human disease is causal, and thus whether reduction of CRP levels will lower the risk of disease. Here, we investigated the causality of CRP in 32 phenotypes by leveraging very large sample sizes collected by GWAS consortia, an approach that was much better powered than most previous MR studies. We found that genetically elevated CRP levels approximated by powerful instruments did not appear to contribute directly to most of the studied somatic and psychiatric outcomes. Our findings are consistent with previous MR studies reporting null associations of genetically elevated CRP levels with inflammation-related outcomes including CAD [56,59,97], type 2 diabetes [98], high body mass index [99], Alzheimer disease, and depression [100]. All previous MR studies were substantially limited to a single or a few outcomes, used only SNPs in the *CRP* gene, or had sample sizes much smaller than that of the present study (S1 Table). In addition to these studies, the current GWAS data do not corroborate epidemiological observations suggesting that elevated CRP levels are associated with amyotrophic lateral sclerosis [101], Alzheimer disease [102], Parkinson disease [103], and major depressive disorder [104]. Furthermore, patients with immunity-related disorders frequently have a very high CRP level (as high as 100 mg/l) due to their disease status. Our findings may therefore more favorably indicate reverse causality. Taken together, these results show that CRP is highly unlikely to contribute causally to most of the major common somatic and neuropsychiatric outcomes that were investigated in the present study, with the possible exception of schizophrenia.

#### Strength of instrumental variables

The results presented in Table 2 show that our GRS_*CRP*_ is not a weak instrument, as indicated by its high *F*-values owing to the large sample sizes of available outcomes from GWASs for the phenotypes under study. The strength of our instrument increased considerably in all disease classes when we used variants of multiple loci associated with CRP in GWASs. However, the variants comprising the CRP GRS_*GWAS*_ explain on average only a moderate ~5% of the total variance in baseline CRP levels [30]. Moreover, the possibility of effect modification by nongenetic CRP-related factors on the outcomes remains to be investigated. We may be able to create even stronger instruments based on ongoing efforts to identify additional variation influencing CRP levels. Even if larger sample sizes and stronger instruments can be realized, the overwhelming lack of causal effects observed for most outcomes in our study implies that therapies targeted at lowering CRP will not directly result in decreased risk of the investigated outcomes, or in better symptom management [105,106].

#### Using summary statistics instead of individual-level data

Here we used summary association statistics obtained from previously conducted meta-GWASs in order to maximize our study power. One may argue this may induce bias compared to when one uses individual-level data. Nevertheless, previous studies showed high agreement in results from MR methods using GWAS summary data and individual-level data [60,107]; Furthermore, our analyses of individual-level data for schizophrenia led to the same conclusion as our analyses using summary statistics data, confirming the robustness of our methodological approach.

#### Other potential sources of bias

An important rationale for MR is that the gene variants do not change over time and are inherited randomly. Thus, the genetic variants are considered free from confounding and reverse causation [108]. However, one cannot completely control for the possibility of confounding of genotype–intermediate phenotype–disease associations. For instance, there could be a confounding effect by ethnic/racial group (i.e., population stratification), but this is unlikely to be a major problem in most situations [108]. In the present study, we included summary statistics data from highly credible results of meta-GWASs. All the original meta-GWASs corrected for population stratification in cohort-level analyses and at meta-GWAS level.

Another caveat of MR is that developmental compensation might occur, through a genotype being expressed during fetal development that in turn buffers the effects of either environmental or genetic factors, a process called canalization [108,109]. Therefore, buffering mechanisms could hamper the associations between genetic variants and the outcome of interest. As opposed to this, a lifetime exposure to a risk factor may enhance its effects on the disease [109]. However, it is not clear to what extent genetically determined small changes in any given exposure would be sufficient to induce compensation [108].

All 32 of the meta-GWASs from which instrument summary estimates were taken were performed in individuals of European descent in Europe and the US and included thousands of samples for each outcome (S1 Table), which was also the case for our previous CRP meta-GWAS from which we chose the CRP-associated SNPs to calculate GRS_*GWAS*_. Therefore, the results of this MR study are applicable to individuals of European descent and are not necessarily generalizable to other ethnic groups.

### Conclusion

We showed that elevated CRP levels driven by genetic factors are causally associated with protection against schizophrenia, suggesting that CRP may be one important puzzle piece that leads to an improved understanding of the pathogenesis of schizophrenia. We observed nominal evidence that genetically elevated CRP is causally associated with SBP, DBP, knee osteoarthritis, and bipolar disorder. Based on current GWAS data, we cannot verify any causal effect of CRP on the other 27 common somatic and neuropsychiatric outcomes investigated in the present study. Therefore, disease-associated rise in CRP levels may be a response to the disease process rather than a cause for these 27 outcomes. This implies that interventions to lower CRP levels are unlikely to result in decreased risk for the majority of common complex outcomes.

## Supporting Information

S1 Consortia Consortia coauthors and collaborators. (DOCX)

S1 Data Individual association summary statistics of CRP lead SNPs and/or proxies with traits and diseases. (XLSX)

S1 Fig GRS_*CRP*_ and GRS_*GWAS*_ for each studied outcome. (DOCX)

S1 Financial Disclosure Authors’ funding information. (PDF)

S1 Methods Linkage disequilibrium of the four GRS_*CRP*_ SNPs. (DOCX)

S2 Methods CRP GRS_*GWAS*_ for Alzheimer disease and body mass index. (DOCX)

S3 Methods Web links. (DOCX)

S4 Methods CRP polygenic risk score (CRP_*PRS*_) for schizophrenia. (DOCX)

S5 Methods In silico (gene) pathway analyses highlight the role of interferon in the causal pathway between CRP and schizophrenia. (DOCX)

S1 Table Previous Mendelian randomization analyses using CRP variants as instruments. (XLSX)

S2 Table CRP lead variants used in the genetic risk scores as instrumental variables. (XLSX)

S3 Table Proxy SNPs of CRP lead variants used in the genetic risk scores as instrumental variables. (XLSX)

S4 Table Biologically prioritized candidate gene set associated with CRP used as the input query to the gene set enrichment analysis. (XLSX)

S5 Table Pathway enrichment results for the biologically prioritized candidate gene set associated with CRP used as the input query to the gene set enrichment analysis. (XLSX)

S6 Table Genes (*n* = 144) that were significantly differentially expressed between schizophrenia and unaffected controls in the hippocampus. (XLSX)

S7 Table Pathway enrichment results for 144 genes that were significantly differentially expressed between schizophrenia and unaffected controls in the hippocampus. (XLSX)

S8 Table Biologically prioritized candidate gene set of CRP from Vaez et al. [81] (from S5 Table, “CRP genes,” in blue) and differentially expressed genes in schizophrenia cases versus controls from Hwang et al. [110] (from S7 Table, “SCZ expr genes,” in red) used as the input query to the pathway analysis. (XLSX)

S9 Table Pathway enrichment results for the combined set of the biologically prioritized candidate gene set of CRP from Vaez et al. [81] and differentially expressed genes in schizophrenia cases versus controls from Hwang et al. 2013. (XLSX)

S10 Table List of genes at 108 genome-wide significant loci associated with schizophrenia. (XLSX)

S11 Table Brain and blood eQTL for credible sets of SNPs of the 108 schizophrenia loci. (XLSX)

S12 Table List of genes at 108 genome-wide significant loci associated with schizophrenia (yellow), brain eQTL (red), and blood eQTL (blue). (XLSX)

S13 Table Pathway enrichment results for the list of genes at 108 genome-wide significant loci associated with schizophrenia (yellow) and those associated with brain eQTL (red) and blood eQTL (blue). (XLSX)

## Abbreviations

CAD: coronary artery disease
CCGC: CRP Coronary Heart Disease Genetics Collaboration
CRP: C-reactive protein
CRP_*PRS*_: C-reactive protein polygenic risk score
DBP: diastolic blood pressure
eGFR_cr_: estimated glomerular filtration rate from serum creatinine
eQTL: expression quantitative trait locus
GRS: genetic risk score
GWAS: genome-wide association study
IBD: inflammatory bowel disease
IV: instrumental variable
lnCRP: natural log of CRP level
MR: Mendelian randomization
OR: odds ratio
PC: principal component
PGC: Psychiatric Genomics Consortium
SBP: systolic blood pressure
SNP: single nucleotide polymorphism
s%: symmetric percentage
