# Supplementary material for: Investigating the Causal Relationship of C-Reactive Protein with 32 Complex Somatic and Psychiatric Outcomes: A Large-Scale Cross-Consortium Mendelian Randomization Study
Source: PLoS Med. 2016 Jun 21;13(6):e1001976. doi: 10.1371/journal.pmed.1001976 (PMC4915710; doi:10.1371/journal.pmed.1001976)
Supplement: S1 Consortia — (DOCX) [file pmed.1001976.s001.docx]

Investigating the causal relationship of C-reactive protein with 32 complex somatic and psychiatric outcomes: A large scale cross-consortia Mendelian randomization study.

Supplementary Methods - 6: Consortia co-authors and collaborators

## The Schizophrenia Working Group of the Psychiatric Genomics Consortium 1

1. The Autism Spectrum Disorders Working Group of The Psychiatric Genomics Consortium 9
2. The Genetic and Environmental Risk for Alzheimer’s disease (GERAD1) Consortium 13
3. The International Consortium for Blood Pressure (ICBP) 15
4. The Psoriasis and Psoriatic Arthritis Genetics Consortium 24

## Schizophrenia Working Group of the Psychiatric Genomics Consortium

## As published in: Nature; Article Type: Biology article DOI: 10.1038/nature13595

**Authors:**

Stephan Ripke^1,2^, Benjamin M. Neale^1,2,3,4^, Aiden Corvin^5^, James T. R. Walters^6^, Kai-How Farh^1^, Peter A. Holmans^6,7^, Phil Lee^1,2,4^, Brendan Bulik-Sullivan^1,2^, David A. Collier^8,9^, Hailiang Huang^1,3^, Tune H. Pers^3,10,11^, Ingrid Agartz^12,13,14^, Esben Agerbo^15,16,17^, Margot Albus^18^, Madeline Alexander^19^, Farooq Amin^20,21^, Silviu A. Bacanu^22^, Martin Begemann^23^, Richard A Belliveau Jr^2^, Judit Bene^24,25^, Sarah E. Bergen ^2,26^, Elizabeth Bevilacqua^2^, Tim B Bigdeli ^22^, Donald W. Black^27^, Richard Bruggeman^28^, Nancy G. Buccola^29^, Randy L. Buckner^30,31,32^, William Byerley^33^, Wiepke Cahn^34^, Guiqing Cai^35,36^, Murray J. Cairns^39,120,170^, Dominique Campion^37^, Rita M. Cantor^38^, Vaughan J. Carr^39,40^, Noa Carrera^6^, Stanley V. Catts^39,41^, Kimberly D. Chambert^2^, Raymond C. K. Chan^42^, Ronald Y. L. Chen^43^, Eric Y. H. Chen^43,44^, Wei Cheng^45^, Eric F. C. Cheung^46^, Siow Ann Chong^47^, C. Robert Cloninger^48^, David Cohen^49^, Nadine Cohen^50^, Paul Cormican^5^, Nick Craddock^6,7^, Benedicto Crespo-Facorro^210^, James J. Crowley^51^, David Curtis^52,53^, Michael Davidson^54^, Kenneth L. Davis^36^, Franziska Degenhardt^55,56^, Jurgen Del Favero^57^, Lynn E. DeLisi^128,129^ , Ditte Demontis^17,58,59^, Dimitris Dikeos^60^, Timothy Dinan^61^, Srdjan Djurovic^14,62^, Gary Donohoe^5,63^, Elodie Drapeau^36^, Jubao Duan^64,65^, Frank Dudbridge^66^, Naser Durmishi^67^, Peter Eichhammer^68^, Johan Eriksson^69,70,71^, Valentina Escott-Price^6^, Laurent Essioux^72^, Ayman H. Fanous^73,74,75,76^, Martilias S. Farrell^51^, Josef Frank^77^, Lude Franke^78^, Robert Freedman^79^, Nelson B. Freimer^80^, Marion Friedl^81^, Joseph I. Friedman^36^, Menachem Fromer^1,2,4,82^, Giulio Genovese^2^, Lyudmila Georgieva^6^, Elliot S. Gershon^209^, Ina Giegling^81,83^, Paola Giusti-Rodríguez^51^, Stephanie Godard^84^, Jacqueline I. Goldstein^1,3^, Vera Golimbet^85^, Srihari Gopal^86^, Jacob Gratten^87^, Lieuwe de Haan^88^, Christian Hammer^23^, Marian L. Hamshere^6^, Mark Hansen^89^, Thomas Hansen^17,90^, Vahram Haroutunian^36,91,92^, Annette M. Hartmann^81^, Frans A. Henskens^39,93,94^, Stefan Herms^55,56,95^, Joel N. Hirschhorn^3,11,96^, Per Hoffmann^55,56,95^, Andrea Hofman^55,56^, Mads V. Hollegaard^97^, David M. Hougaard^97^, Masashi Ikeda^98^, Inge Joa^99^, Antonio Julià^100^, René S. Kahn^34^, Luba Kalaydjieva^101,102^, Sena Karachanak-Yankova^103^, Juha Karjalainen^78^, David Kavanagh^6^, Matthew C. Keller^104^, Brian J. Kelly^120^, James L. Kennedy^105,106,107^, Andrey Khrunin^108^, Yunjung Kim^51^, Janis Klovins^109^, James A. Knowles^110^, Bettina Konte^81^, Vaidutis Kucinskas^111^, Zita Ausrele Kucinskiene^111^, Hana Kuzelova-Ptackova^112^, Anna K. Kähler^26^, Claudine Laurent^19,113^, Jimmy Lee Chee Keong^47,114^, S. Hong Lee^87^, Sophie E. Legge^6^, Bernard Lerer^115^, Miaoxin Li^43,44,116^ Tao Li^117^, Kung-Yee Liang^118^, Jeffrey Lieberman^119^, Svetlana Limborska^108^, Carmel M. Loughland^39,120^, Jan Lubinski^121^, Jouko Lönnqvist^122^, Milan Macek Jr^112^, Patrik K. E. Magnusson^26^, Brion S. Maher^123^, Wolfgang Maier^124^, Jacques Mallet^125^, Sara Marsal^100^, Manuel Mattheisen^17,58,59,126^, Morten Mattingsdal^14,127^, Robert W. McCarley^128,129^, Colm McDonald^130^, Andrew M. McIntosh^131,132^, Sandra Meier^77^, Carin J. Meijer^88^, Bela Melegh^24,25^, Ingrid Melle^14,133^, Raquelle I. Mesholam-Gately^128,134^, Andres Metspalu^135^, Patricia T. Michie^39,136^, Lili Milani^135^, Vihra Milanova^137^, Younes Mokrab^8^, Derek W. Morris^5,63^, Ole Mors^17,58,138^, Kieran C. Murphy^139^, Robin M. Murray^140^, Inez Myin-Germeys^141^, Bertram Müller-Myhsok^142,143,144^, Mari Nelis^135^, Igor Nenadic^145^, Deborah A. Nertney^146^, Gerald Nestadt^147^, Kristin K. Nicodemus^148^, Liene Nikitina-Zake^109^, Laura Nisenbaum^149^, Annelie Nordin^150^, Eadbhard O’Callaghan^151^, Colm O’Dushlaine^2^, F. Anthony O’Neill^152^, Sang-Yun Oh^153^, Ann Olincy^79^, Line Olsen^17,90^, Jim Van Os^141,154^, Psychosis Endophenotypes International Consortium^155^, Christos Pantelis^39,156^, George N. Papadimitriou^60^, Sergi Papiol^23^, Elena Parkhomenko^36^, Michele T. Pato^110^, Tiina Paunio^157,158^, Milica Pejovic-Milovancevic^159^, Diana O. Perkins^160^, Olli Pietiläinen^158,161^, Jonathan Pimm^53^, Andrew J. Pocklington^6^, John Powell^140^, Alkes Price^3^,^162^, Ann E. Pulver^147^, Shaun M. Purcell^82^, Digby Quested^163^, Henrik B. Rasmussen^17,90^, Abraham Reichenberg^36^, Mark A. Reimers^164^, Alexander L. Richards^6^, Joshua L. Roffman^30,32^, Panos Roussos^82,165^, Douglas M. Ruderfer^6,82^, Veikko Salomaa^71^, Alan R. Sanders^64,65^, Ulrich Schall^39,120^, Christian R. Schubert^166^, Thomas G. Schulze^77,167^, Sibylle G. Schwab^168^, Edward M. Scolnick^2^, Rodney J. Scott^39,169,170^, Larry J. Seidman^128,134^, Jianxin Shi^171^, Engilbert Sigurdsson^172^, Teimuraz Silagadze^173^, Jeremy M. Silverman^36,174^, Kang Sim^47^, Petr Slominsky^108^, Jordan W. Smoller^2,4^, Hon-Cheong So^43^, Chris C. A. Spencer^175^, Eli A. Stahl^3,82^, Hreinn Stefansson^176^, Stacy Steinberg^176^, Elisabeth Stogmann^177^, Richard E. Straub^178^, Eric Strengman^179,34^, Jana Strohmaier^77^, T. Scott Stroup^119^, Mythily Subramaniam^47^, Jaana Suvisaari^122^, Dragan M. Svrakic^48^, Jin P. Szatkiewicz^51^, Erik Söderman^12^, Srinivas Thirumalai^180^, Draga Toncheva^103^, Paul A. Tooney^39,120,170^ , Sarah Tosato^181^, Juha Veijola^182,183^, John Waddington^184^, Dermot Walsh^185^, Dai Wang^86^, Qiang Wang^117^, Bradley T. Webb^22^, Mark Weiser^54^, Dieter B. Wildenauer^186^, Nigel M. Williams^6^, Stephanie Williams^51^, Stephanie H. Witt^77^, Aaron R. Wolen^164^, Emily H. M. Wong^43^, Brandon K. Wormley^22^, Jing Qin Wu^39,170^, Hualin Simon Xi^187^, Clement C. Zai^105,106^, Xuebin Zheng^188^, Fritz Zimprich^177^, Naomi R. Wray^87^, Kari Stefansson^176^, Peter M. Visscher^87^, Wellcome Trust Case-Control Consortium 2^189^, Rolf Adolfsson^150^, Ole A. Andreassen^14,133^, Douglas H. R. Blackwood^132^, Elvira Bramon^190^, Joseph D. Buxbaum^35,36,91,191^, Anders D. Børglum^17,58,59,138^, Sven Cichon^55,56,95,192^, Ariel Darvasi^193^, Enrico Domenici^194^, Hannelore Ehrenreich^23^, Tõnu Esko^3,11,96,135^, Pablo V. Gejman^64,65^, Michael Gill^5^, Hugh Gurling^53^, Christina M. Hultman^26^, Nakao Iwata^98^, Assen V. Jablensky^39,102,186,195^, Erik G. Jönsson^12,14^, Kenneth S. Kendler^196^, George Kirov^6^, Jo Knight^105,106,107^, Todd Lencz^197,198,199^, Douglas F. Levinson^19^, Qingqin S. Li^86^, Jianjun Liu^188,200^, Anil K. Malhotra^197,198,199^, Steven A. McCarroll^2,96^, Andrew McQuillin^53^, Jennifer L. Moran^2^, Preben B. Mortensen^15,16,17^, Bryan J. Mowry^87,201^, Markus M. Nöthen^55,56^, Roel A. Ophoff^38,80,34^, Michael J. Owen^6,7^, Aarno Palotie^2,4,161^, Carlos N. Pato^110^, Tracey L. Petryshen^2,128,202^, Danielle Posthuma^203,204,205^, Marcella Rietschel^77^, Brien P. Riley^196^, Dan Rujescu^81,83^, Pak C. Sham^43,44,116^ Pamela Sklar^82,91,165^, David St Clair^206^, Daniel R. Weinberger^178,207^, Jens R. Wendland^166^, Thomas Werge^17,90,208^, Mark J. Daly^1,2,3^, Patrick F. Sullivan^26,51,160^ & Michael C. O’Donovan^6,7^

**Affiliations**

^1^Analytic and Translational Genetics Unit, Massachusetts General Hospital, Boston, Massachusetts 02114, USA. ^2^Stanley Center for Psychiatric Research, Broad Institute of MIT and Harvard, Cambridge, Massachusetts 02142, USA. ^3^Medical and Population Genetics Program, Broad Institute of MIT and Harvard, Cambridge, Massachusetts 02142, USA. ^4^Psychiatric and Neurodevelopmental Genetics Unit, Massachusetts General Hospital, Boston, Massachusetts 02114, USA. ^5^Neuropsychiatric Genetics Research Group, Department of Psychiatry, Trinity College Dublin, Dublin 8, Ireland. ^6^MRC Centre for Neuropsychiatric Genetics and Genomics, Institute of Psychological Medicine and Clinical Neurosciences, School of Medicine, Cardiff University, Cardiff, CF24 4HQ, UK. ^7^National Centre for Mental Health, Cardiff University, Cardiff, CF24 4HQ, UK. ^8^Eli Lilly and Company Limited, Erl Wood Manor, Sunninghill Road, Windlesham, Surrey, GU20 6PH, UK. ^9^Social, Genetic and Developmental Psychiatry Centre, Institute of Psychiatry, King’s College London, London, SE5 8AF, UK. ^10^Center for Biological Sequence Analysis, Department of Systems Biology, Technical University of Denmark, DK-2800, Denmark. ^11^Division of Endocrinology and Center for Basic and Translational Obesity Research, Boston Children’s Hospital, Boston, Massachusetts, 02115USA. ^12^Department of Clinical Neuroscience, Psychiatry Section, Karolinska Institutet, SE-17176 Stockholm, Sweden. ^13^Department of Psychiatry, Diakonhjemmet Hospital, 0319 Oslo, Norway. ^14^NORMENT, KG Jebsen Centre for Psychosis Research, Institute of Clinical Medicine, University of Oslo, 0424 Oslo, Norway. ^15^Centre for Integrative Register-based Research, CIRRAU, Aarhus University, DK-8210 Aarhus, Denmark. ^16^National Centre for Register-based Research, Aarhus University, DK-8210 Aarhus, Denmark. ^17^The Lundbeck Foundation Initiative for Integrative Psychiatric Research, iPSYCH, Denmark. ^18^State Mental Hospital, 85540 Haar, Germany. ^19^Department of Psychiatry and Behavioral Sciences, Stanford University, Stanford, California 94305, USA. ^20^Department of Psychiatry and Behavioral Sciences, Atlanta Veterans Affairs Medical Center, Atlanta, Georgia 30033, USA. ^21^Department of Psychiatry and Behavioral Sciences, Emory University, Atlanta Georgia 30322, USA. ^22^Virginia Institute for Psychiatric and Behavioral Genetics, Department of Psychiatry, Virginia Commonwealth University, Richmond, Virginia 23298, USA. ^23^Clinical Neuroscience, Max Planck Institute of Experimental Medicine, Göttingen 37075, Germany. ^24^Department of Medical Genetics, University of Pécs, Pécs H-7624, Hungary. ^25^Szentagothai Research Center, University of Pécs, Pécs H-7624, Hungary.

^26^Department of Medical Epidemiology and Biostatistics, Karolinska Institutet, Stockholm SE-17177, Sweden.

^27^Department of Psychiatry, University of Iowa Carver College of Medicine, Iowa City, Iowa 52242, USA. ^28^University Medical Center Groningen, Department of Psychiatry, University of Groningen NL-9700 RB, The Netherlands. ^29^School of Nursing, Louisiana State University Health Sciences Center, New Orleans, Louisiana 70112, USA. ^30^Athinoula A. Martinos Center, Massachusetts General Hospital, Boston, Massachusetts 02129, USA. ^31^Center for Brain Science, Harvard University, Cambridge, Massachusetts, 02138 USA. ^32^Department of Psychiatry, Massachusetts General Hospital, Boston, Massachusetts, 02114 USA. ^33^Department of Psychiatry, University of California at San Francisco, San Francisco, California, 94143 USA. ^34^University Medical Center Utrecht, Department of Psychiatry, Rudolf Magnus Institute of Neuroscience, 3584 Utrecht, The Netherlands. ^35^Department of Human Genetics, Icahn School of Medicine at Mount Sinai, New York, New York 10029 USA. ^36^Department of Psychiatry, Icahn School of Medicine at Mount Sinai, New York, New York 10029 USA. ^37^Centre Hospitalier du Rouvray and INSERM U1079 Faculty of Medicine, 76301 Rouen, France. ^38^Department of Human Genetics, David Geffen School of Medicine, University of California, Los Angeles, California 90095, USA. ^39^Schizophrenia Research Institute, Sydney NSW 2010, Australia. ^40^School of Psychiatry, University of New South Wales, Sydney NSW 2031, Australia. ^41^Royal Brisbane and Women’s Hospital, University of Queensland, Brisbane, St Lucia QLD 4072, Australia. ^42^Institute of Psychology, Chinese Academy of Science, Beijing 100101, China. ^43^Department of Psychiatry, Li Ka Shing Faculty of Medicine, The University of Hong Kong, Hong Kong, China. ^44^State Key Laboratory for Brain and Cognitive Sciences, Li Ka Shing Faculty of Medicine, The University of Hong Kong, Hong Kong, China. ^45^Department of Computer Science, University of North Carolina, Chapel Hill, North Carolina 27514, USA. ^46^Castle Peak Hospital, Hong Kong, China. ^47^Institute of Mental Health, Singapore 539747, Singapore. ^48^Department of Psychiatry, Washington University, St. Louis, Missouri 63110, USA. ^49^Department of Child and Adolescent Psychiatry, Assistance Publique Hopitaux de Paris, Pierre and Marie Curie Faculty of Medicine and Institute for Intelligent Systems and Robotics, Paris, 75013, France. ^50^ Blue Note Biosciences, Princeton, New Jersey 08540, USA. ^51^Department of Genetics, University of North Carolina, Chapel Hill, North Carolina 27599-7264, USA. ^52^Department of Psychological Medicine, Queen Mary University of London, London E1 1BB, UK. ^53^Molecular Psychiatry Laboratory, Division of Psychiatry, University College London, London WC1E 6JJ, UK. ^54^Sheba Medical Center, Tel Hashomer 52621, Israel. ^55^Department of Genomics, Life and Brain Center, D-53127 Bonn, Germany. ^56^Institute of Human Genetics, University of Bonn, D-53127 Bonn, Germany. ^57^Applied Molecular Genomics Unit, VIB Department of Molecular Genetics, University of Antwerp, B-2610 Antwerp, Belgium. ^58^Centre for Integrative Sequencing, iSEQ, Aarhus University, DK-8000 Aarhus C, Denmark. ^59^Department of Biomedicine, Aarhus University, DK-8000 Aarhus C, Denmark. ^60^First Department of Psychiatry, University of Athens Medical School, Athens 11528, Greece. ^61^Department of Psychiatry, University College Cork, Co. Cork, Ireland. ^62^Department of Medical Genetics, Oslo University Hospital, 0424 Oslo, Norway. ^63^Cognitive Genetics and Therapy Group, School of Psychology and Discipline of Biochemistry, National University of Ireland Galway, Co. Galway, Ireland. ^64^Department of Psychiatry and Behavioral Neuroscience, University of Chicago, Chicago, Illinois 60637, USA. ^65^Department of Psychiatry and Behavioral Sciences, NorthShore University HealthSystem, Evanston, Illinois 60201, USA. ^66^Department of Non-Communicable Disease Epidemiology, London School of Hygiene and Tropical Medicine, London WC1E 7HT, UK. ^67^Department of Child and Adolescent Psychiatry, University Clinic of Psychiatry, Skopje 1000, Republic of Macedonia. ^68^Department of Psychiatry, University of Regensburg, 93053 Regensburg, Germany. ^69^Department of General Practice, Helsinki University Central Hospital, University of Helsinki P.O. Box 20, Tukholmankatu 8 B, FI-00014, Helsinki, Finland. ^70^Folkhälsan Research Center, Helsinki, Finland, Biomedicum Helsinki 1, Haartmaninkatu 8, FI-00290, Helsinki, Finland. ^71^National Institute for Health and Welfare, P.O. BOX 30, FI-00271 Helsinki, Finland. ^72^Translational Technologies and Bioinformatics, Pharma Research and Early Development, F. Hoffman-La Roche, CH-4070 Basel, Switzerland. ^73^Department of Psychiatry, Georgetown University School of Medicine, Washington DC 20057, USA. ^74^Department of Psychiatry, Keck School of Medicine of the University of Southern California, Los Angeles, California 90033, USA. ^75^Department of Psychiatry, Virginia Commonwealth University School of Medicine, Richmond, Virginia 23298, USA. ^76^Mental Health Service Line, Washington VA Medical Center, Washington DC 20422, USA. ^77^Department of Genetic Epidemiology in Psychiatry, Central Institute of Mental Health, Medical Faculty Mannheim, University of Heidelberg, Heidelberg , D-68159 Mannheim, Germany. ^78^Department of Genetics, University of Groningen, University Medical Centre Groningen, 9700 RB Groningen, The Netherlands. ^79^Department of Psychiatry, University of Colorado Denver, Aurora, Colorado 80045, USA. ^80^Center for Neurobehavioral Genetics, Semel Institute for Neuroscience and Human Behavior, University of California, Los Angeles, California 90095, USA. ^81^Department of Psychiatry, University of Halle, 06112 Halle, Germany. ^82^Division of Psychiatric Genomics, Department of Psychiatry, Icahn School of Medicine at Mount Sinai, New York, New York 10029, USA. ^83^Department of Psychiatry, University of Munich, 80336, Munich, Germany. ^84^Departments of Psychiatry and Human and Molecular Genetics, INSERM, Institut de Myologie, Hôpital de la Pitiè-Salpêtrière, Paris, 75013, France. ^85^Mental Health Research Centre, Russian Academy of Medical Sciences, 115522 Moscow, Russia. ^86^Neuroscience Therapeutic Area, Janssen Research and Development, Raritan, New Jersey 08869, USA. ^87^Queensland Brain Institute, The University of Queensland, Brisbane, Queensland, QLD 4072, Australia. ^88^Academic Medical Centre University of Amsterdam, Department of Psychiatry, 1105 AZ Amsterdam, The Netherlands. ^89^Illumina, La Jolla, California, California 92122, USA. ^90^Institute of Biological Psychiatry, Mental Health Centre Sct. Hans, Mental Health Services Copenhagen, DK-4000, Denmark. ^91^Friedman Brain Institute, Icahn School of Medicine at Mount Sinai, New York, New York 10029, USA. ^92^J. J. Peters VA Medical Center, Bronx, New York, New York 10468, USA. ^93^Priority Research Centre for Health Behaviour, University of Newcastle, Newcastle NSW 2308, Australia. ^94^School of Electrical Engineering and Computer Science, University of Newcastle, Newcastle NSW 2308, Australia. ^95^Division of Medical Genetics, Department of Biomedicine, University of Basel, Basel, CH-4058, Switzerland. ^96^Department of Genetics, Harvard Medical School, Boston, Massachusetts 02115, USA. ^97^Section of Neonatal Screening and Hormones, Department of Clinical Biochemistry, Immunology and Genetics, Statens Serum Institut, Copenhagen, DK-2300, Denmark. ^98^Department of Psychiatry, Fujita Health University School of Medicine, Toyoake, Aichi, 470-1192, Japan. ^99^Regional Centre for Clinical Research in Psychosis, Department of Psychiatry, Stavanger University Hospital, 4011 Stavanger, Norway. ^100^Rheumatology Research Group, Vall d'Hebron Research Institute, Barcelona, 08035, Spain. ^101^Centre for Medical Research, The University of Western Australia, Perth, WA 6009, Australia. ^102^The Perkins Institute for Medical Research, The University of Western Australia, Perth, WA 6009, Australia. ^103^Department of Medical Genetics, Medical University, Sofia1431, Bulgaria. ^104^Department of Psychology, University of Colorado Boulder, Boulder, Colorado 80309, USA. ^105^Campbell Family Mental Health Research Institute, Centre for Addiction and Mental Health, Toronto, Ontario, M5T 1R8, Canada. ^106^Department of Psychiatry, University of Toronto, Toronto, Ontario, M5T 1R8, Canada. ^107^Institute of Medical Science, University of Toronto, Toronto, Ontario, M5S 1A8, Canada. ^108^Institute of Molecular Genetics, Russian Academy of Sciences, Moscow123182, Russia. ^109^Latvian Biomedical Research and Study Centre, Riga, LV-1067, Latvia. ^110^Department of Psychiatry and Zilkha Neurogenetics Institute, Keck School of Medicine at University of Southern California, Los Angeles, California 90089, USA. ^111^Faculty of Medicine, Vilnius University, LT-01513 Vilnius, Lithuania. ^112^ Department of Biology and Medical Genetics, 2nd Faculty of Medicine and University Hospital Motol, 150 06 Prague, Czech Republic. ^113^ Department of Child and Adolescent Psychiatry, Pierre and Marie Curie Faculty of Medicine, Paris 75013, France. ^114^Duke-NUS Graduate Medical School, Singapore 169857, Singapore. ^115^Department of Psychiatry, Hadassah-Hebrew University Medical Center, Jerusalem 91120, Israel. ^116^Centre for Genomic Sciences, The University of Hong Kong, Hong Kong, China. ^117^Mental Health Centre and Psychiatric Laboratory, West China Hospital, Sichuan University, Chengdu, 610041, Sichuan, China. ^118^Department of Biostatistics, Johns Hopkins University Bloomberg School of Public Health, Baltimore, Maryland 21205, USA. ^119^Department of Psychiatry, Columbia University, New York, New York 10032, USA. ^120^Priority Centre for Translational Neuroscience and Mental Health, University of Newcastle, Newcastle NSW 2300, Australia. ^121^Department of Genetics and Pathology, International Hereditary Cancer Center, Pomeranian Medical University in Szczecin, 70-453 Szczecin, Poland. ^122^Department of Mental Health and Substance Abuse Services; National Institute for Health and Welfare, P.O. BOX 30, FI-00271 Helsinki, Finland. ^123^Department of Mental Health, Bloomberg School of Public Health, Johns Hopkins University, Baltimore, Maryland 21205, USA. ^124^Department of Psychiatry, University of Bonn, D-53127 Bonn, Germany. ^125^Centre National de la Recherche Scientifique, Laboratoire de Génétique Moléculaire de la Neurotransmission et des Processus Neurodégénératifs, Hôpital de la Pitié Salpêtrière, 75013, Paris, France. ^126^Department of Genomics Mathematics, University of Bonn, D-53127 Bonn, Germany. ^127^Research Unit, Sørlandet Hospital, 4604 Kristiansand, Norway. ^128^Department of Psychiatry, Harvard Medical School, Boston, Massachusetts 02115, USA. ^129^VA Boston Health Care System, Brockton, Massachusetts 02301, USA. ^130^Department of Psychiatry, National University of Ireland Galway, Co. Galway, Ireland. ^131^Centre for Cognitive Ageing and Cognitive Epidemiology, University of Edinburgh, Edinburgh EH16 4SB, UK. ^132^Division of Psychiatry, University of Edinburgh, Edinburgh EH16 4SB, UK. ^133^Division of Mental Health and Addiction, Oslo University Hospital, 0424 Oslo, Norway. ^134^Massachusetts Mental Health Center Public Psychiatry Division of the Beth Israel Deaconess Medical Center, Boston, Massachusetts 02114, USA. ^135^Estonian Genome Center, University of Tartu, Tartu 50090, Estonia. ^136^School of Psychology, University of Newcastle, Newcastle NSW 2308, Australia. ^137^First Psychiatric Clinic, Medical University, Sofia 1431, Bulgaria. ^138^Department P, Aarhus University Hospital, DK-8240 Risskov, Denmark. ^139^Department of Psychiatry, Royal College of Surgeons in Ireland, Dublin 2, Ireland. ^140^King’s College London, London SE5 8AF, UK. ^141^Maastricht University Medical Centre, South Limburg Mental Health Research and Teaching Network, EURON, 6229 HX Maastricht, The Netherlands. ^142^Institute of Translational Medicine, University of Liverpool, Liverpool L69 3BX, UK. ^143^Max Planck Institute of Psychiatry, 80336 Munich, Germany. ^144^Munich Cluster for Systems Neurology (SyNergy), 80336 Munich, Germany. ^145^Department of Psychiatry and Psychotherapy, Jena University Hospital, 07743 Jena, Germany. ^146^Department of Psychiatry, Queensland Brain Institute and Queensland Centre for Mental Health Research, University of Queensland, Brisbane, Queensland, St Lucia QLD 4072, Australia. ^147^Department of Psychiatry and Behavioral Sciences, Johns Hopkins University School of Medicine, Baltimore, Maryland 21205, USA. ^148^Department of Psychiatry, Trinity College Dublin, Dublin 2, Ireland. ^149^Eli Lilly and Company, Lilly Corporate Center, Indianapolis, 46285 Indiana, USA. ^150^Department of Clinical Sciences, Psychiatry, Umeå University, SE-901 87 Umeå, Sweden. ^151^DETECT Early Intervention Service for Psychosis, Blackrock, Co. Dublin, Ireland. ^152^Centre for Public Health, Institute of Clinical Sciences, Queen’s University Belfast, Belfast BT12 6AB, UK. ^153^Lawrence Berkeley National Laboratory, University of California at Berkeley, Berkeley, California 94720, USA. ^154^Institute of Psychiatry, King’s College London, London SE5 8AF, UK. ^155^A list of authors and affiliations appear in the Supplementary Information. ^156^Melbourne Neuropsychiatry Centre, University of Melbourne & Melbourne Health, Melbourne, Vic 3053, Australia. ^157^Department of Psychiatry, University of Helsinki, P.O. Box 590, FI-00029 HUS, Helsinki, Finland. ^158^Public Health Genomics Unit, National Institute for Health and Welfare, P.O. BOX 30, FI-00271 Helsinki, Finland. ^159^Medical Faculty, University of Belgrade, 11000 Belgrade, Serbia. ^160^Department of Psychiatry, University of North Carolina, Chapel Hill, North Carolina 27599-7160, USA. ^161^Institute for Molecular Medicine Finland, FIMM, University of Helsinki, P.O. Box 20 FI-00014, Helsinki, Finland. ^162^Department of Epidemiology, Harvard School of Public Health, Boston, Massachusetts 02115, USA. ^163^Department of Psychiatry, University of Oxford, Oxford, OX3 7JX, UK. ^164^Virginia Institute for Psychiatric and Behavioral Genetics, Virginia Commonwealth University, Richmond, Virginia 23298, USA. ^165^Institute for Multiscale Biology, Icahn School of Medicine at Mount Sinai, New York, New York 10029, USA. ^166^PharmaTherapeutics Clinical Research, Pfizer Worldwide Research and Development, Cambridge, Massachusetts 02139, USA. ^167^Department of Psychiatry and Psychotherapy, University of Gottingen, 37073 Göttingen, Germany. ^168^Psychiatry and Psychotherapy Clinic, University of Erlangen, 91054 Erlangen, Germany. ^169^Hunter New England Health Service, Newcastle NSW 2308, Australia. ^170^School of Biomedical Sciences and Pharmacy, University of Newcastle, Callaghan NSW 2308, Australia. ^171^Division of Cancer Epidemiology and Genetics, National Cancer Institute, Bethesda, Maryland 20892, USA. ^172^University of Iceland, Landspitali, National University Hospital, 101 Reykjavik, Iceland. ^173^Department of Psychiatry and Drug Addiction, Tbilisi State Medical University (TSMU), **N33, 0177** Tbilisi, Georgia. ^174^Research and Development, Bronx Veterans Affairs Medical Center, New York, New York 10468, USA. ^175^Wellcome Trust Centre for Human Genetics, Oxford, OX3 7BN, UK. ^176^deCODE Genetics, 101 Reykjavik, Iceland. ^177^Department of Clinical Neurology, Medical University of Vienna, 1090 Wien, Austria. ^178^Lieber Institute for Brain Development, Baltimore, Maryland 21205, USA. ^179^Department of Medical Genetics, University Medical Centre Utrecht, Universiteitsweg 100, 3584 CG, Utrecht, The Netherlands. ^180^Berkshire Healthcare NHS Foundation Trust, Bracknell RG12 1BQ, UK. ^181^Section of Psychiatry, University of Verona, 37134 Verona, Italy. ^182^Department of Psychiatry, University of Oulu, P.O. BOX 5000, 90014, Finland. ^183^University Hospital of Oulu, P.O.BOX 20, 90029 OYS, Finland. ^184^Molecular and Cellular Therapeutics, Royal College of Surgeons in Ireland, Dublin 2, Ireland. ^185^Health Research Board, Dublin 2, Ireland. ^186^School of Psychiatry and Clinical Neurosciences, The University of Western Australia, Perth WA6009, Australia. ^187^Computational Sciences CoE, Pfizer Worldwide Research and Development, Cambridge, Massachusetts 02139, USA. ^188^Human Genetics, Genome Institute of Singapore, A*STAR, Singapore 138672, Singapore. ^189^A list of authors and affiliations appear in the Supplementary Information. ^190^University College London, London WC1E 6BT, UK. ^191^Department of Neuroscience, Icahn School of Medicine at Mount Sinai, New York, New York 10029, USA. ^192^Institute of Neuroscience and Medicine (INM-1), Research Center Juelich, 52428 Juelich, Germany. ^193^Department of Genetics, The Hebrew University of Jerusalem, 91905 Jerusalem, Israel. ^194^Neuroscience Discovery and Translational Area, Pharma Research and Early Development, F. Hoffman-La Roche, CH-4070 Basel, Switzerland. ^195^Centre for Clinical Research in Neuropsychiatry, School of Psychiatry and Clinical Neurosciences, The University of Western Australia, Medical Research Foundation Building, Perth WA 6000, Australia. ^196^Virginia Institute for Psychiatric and Behavioral Genetics, Departments of Psychiatry and Human and Molecular Genetics, Virginia Commonwealth University, Richmond, Virginia 23298, USA. ^197^The Feinstein Institute for Medical Research, Manhasset, New York, 11030 USA. ^198^The Hofstra NS-LIJ School of Medicine, Hempstead, New York, 11549 USA. ^199^The Zucker Hillside Hospital, Glen Oaks, New York,11004 USA. ^200^Saw Swee Hock School of Public Health, National University of Singapore, Singapore 117597, Singapore. ^201^Queensland Centre for Mental Health Research, University of Queensland, Brisbane 4076, Queensland, Australia. ^202^Center for Human Genetic Research and Department of Psychiatry, Massachusetts General Hospital, Boston, Massachusetts 02114, USA. ^203^Department of Child and Adolescent Psychiatry, Erasmus University Medical Centre, Rotterdam 3000, The Netherlands. ^204^Department of Complex Trait Genetics, Neuroscience Campus Amsterdam, VU University Medical Center Amsterdam, Amsterdam 1081, The Netherlands. ^205^Department of Functional Genomics, Center for Neurogenomics and Cognitive Research, Neuroscience Campus Amsterdam, VU University, Amsterdam 1081, The Netherlands. ^206^University of Aberdeen, Institute of Medical Sciences, Aberdeen, AB25 2ZD, UK. ^207^Departments of Psychiatry, Neurology, Neuroscience and Institute of Genetic Medicine, Johns Hopkins School of Medicine, Baltimore, Maryland 21205, USA. ^208^Department of Clinical Medicine, University of Copenhagen, Copenhagen 2200, Denmark. ^209^Departments of Psychiatry and Human Genetics, University of Chicago, Chicago, Illinois 60637, USA. ^210^University Hospital Marqués de Valdecilla, Instituto de Formación e Investigación Marqués de Valdecilla, University of Cantabria, E‐39008 Santander, Spain.

## The Autism Spectrum Disorders Working Group of The Psychiatric Genomics Consortium ^,‡^

Please address correspondence to: **pgc.autism@gmail.com**

**Authors:**

Richard J. L. Anney^1,2,*^, Stephan Ripke^3,4,*^, Verneri Anttila^3,4^, Peter Holmans^2^, Hailiang Huang^3,4^, Lambertus Klei^5^, Phil H. Lee^3,4,6^, Sarah E. Medland^7^, Benjamin Neale^3,4^, Elise Robinson^3,4^, Lauren A. Weiss^8,9^, Joana Almeida^10^, David Amaral^11,12,13^, Evdokia Anagnostou^14^, Elena Bacchelli^15^, Joel S. Bader^16^, Anthony J. Bailey^17,18^, Gillian Baird^19^, Vanessa H. Bal^8^, Agatino Battaglia^20^, Arthur L. Beaudet^21^, Raphael Bernier^22^, Catalina Betancur^23,24,25^, Nadia Bolshakova^1^, Sven Bölte^26,27,28^, Patrick F. Bolton^29,30^, Thomas Bourgeron^31,32,33,34^, Sean Brennan^1^, Cátia Café^10^, Rita M. Cantor^35,36^, Jillian Casey^37,38^, Patrícia B. S. Celestino-Soper^21,39^, Andreas G. Chiocchetti^26^, Ines C. Conceição^40,41^, Judith Conroy^37,38^, Catarina T. Correia^40,41^, Michael L. Cuccaro^42^, Geraldine Dawson^43,44^, Maretha V. De Jonge^45^, Silvia De Rubeis^46,47^, Richard Delorme^31,32,33,48^, Eftichia Duketis^26^, Frederico Duque^10,49^, Sean Ennis^38,50^, A. Gulhan Ercan-Sencicek^51^, M. Daniele Fallin^52^, Bridget Fernandez^53^, Susan E. Folstein^54^, Eric Fombonne^55^, Christine M. Freitag^26^, Louise Gallagher^1^, John Gilbert^42^, Christopher Gillberg^56^, Arthur P. Goldberg^46,47^, Jonathan M. Green^57,58^, Andrew Green^38,50^, Dorothy E. Grice^47^, Stephen J. Guter^59^, Jonathan L. Haines^60^, Robert Hendren^8^, Irva Hertz-Picciotto^11,61^, Christina M. Hultman^62^, Bozenna Iliadou^62^, Suma Jacob^59,63^, Sabine M. Klauck^64^, Alexander Kolevzon^46,47,65,66^, Christine Ladd-Acosta^52^, Ann S. Le Couteur^67^, Marion Leboyer^31,68,69,70^, David H. Ledbetter^71^, Christa Lese Martin^72^, Pat Levitt^73^, Catherine Lord^74^, Jennifer K. Lowe^75,76,77^, Elena Maestrini^15^, Tiago Magalhaes^38,78^, Shrikant M. Mane^79^, Donna M. Martin^80^, Susan G. McGrew^81^, William M. McMahon^82^, Alison Merikangas^1^, Nancy Minshew^5^, Anthony P. Monaco^83,84^, Daniel Moreno-De-Luca^85^, Eric M. Morrow^86^, Susana Mouga^10,49^, Michael T. Murtha^51^, John I. Nurnberger^39,87^, Guiomar Oliveira^10,49^, Alistair T. Pagnamenta^83^, Jeremy R. Parr^67^, Andrew D. Paterson^88,89,90^, Margaret A. Pericak-Vance^42^, Dalila Pinto^46,47,65,66,91,92^, Joseph Piven^93^, Christopher S. Poultney^46,47^, Fritz Poustka^26^, Regina Regan^38,78^, Karola Rehnström^94^, Abraham Reichenberg^46,47^, Jennifer Reichert^46,47^, Wendy Roberts^95^, Kathryn Roeder^96,97^, Bernadette Rogé^98^, Guy A. Rouleau^99^, Stephan J. Sanders^8^, Sven Sandin^62^, Gerard D. Schellenberg^100^, Stephen W. Scherer^88,89,101^, Latha Soorya^46,47,102^, Matthew W. State^8^, Oscar Svantesson^62^, Peter Szatmari^103^, Ann P. Thompson^104^, Kathryn Tsang^8,9^, Herman van Engeland^45^, Astrid M. Vicente^40,41^, Veronica J. Vieland^105^, Jacob A. S. Vorstman^45^, Simon Wallace^17^, Christopher A. Walsh^106,107,108,109,110^, Regina Waltes^26^, Thomas H. Wassink^111^, Ellen M. Wijsman^112,113^, A. Jeremy Willsey^8^, Kerstin Wittemeyer^114^, Timothy W. Yu^106^, Lonnie Zwaigenbaum^115^, Joseph D. Buxbaum^46,47,65,66,91,116^, Aravinda Chakravarti^16^, Edwin H. Cook^59^, Hilary Coon^82^, Daniel H. Geschwind^36,75,76,77^, Michael Gill^1^, Hakon Hakonarson^117,118^, Joachim Hallmayer^119^, Aarno Palotie^94^, Susan Santangelo^120^, James S. Sutcliffe^60,121^, Dan Arking^16,†^, Bernie Devlin^5,†^, and Mark J. Daly^3,4,†^

**Affiliations:**
^1^Dept. of Psychiatry, Trinity College Dublin, Dublin, Ireland. ^2^MRC Centre for Neuropsychiatric Genetics & Genomics, Cardiff University, Cardiff, UK. ^3^Analytic and Translational Genetics Unit, Dept. of Medicine, Massachusetts General Hospital and Harvard Medical School, Boston, MA, USA. ^4^Stanley Center for Psychiatric Research and Program in Medical and Population Genetic, Broad Institute of Harvard and MIT, Cambridge, MA, USA. ^5^Dept. of Psychiatry, University of Pittsburgh School of Medicine, Pittsburgh, PA, USA. ^6^Dept. of Psychiatry, Harvard Medical School, Boston, MA, USA.
^7^Queensland Institute of Medical Research, Brisbane, QLD, Australia. ^8^Dept. of Psychiatry, University of California San Francisco, San Francisco, CA, USA. ^9^Inst. Human Genetics, University of California San Francisco, San Francisco, CA, USA. ^10^Unidade de Neurodesenvolvimento e Autismo do Serviço do Centro de Desenvolvimento da Criança and Centro de Investigação e Formação Clinica, Pediatric Hospital, Centro Hospitalar e Universitário de Coimbra, Coimbra, Portugal. ^11^The MIND Institute, School of Medicine, University of California Davis, Davis, CA, USA. ^12^Dept. of Psychiatry, School of Medicine, University of California Davis, Davis, CA, USA. ^13^Dept. of Behavioural Sciences, School of Medicine, University of California Davis, Davis, CA,USA. ^14^Bloorview Research Institute, University of Toronto, Toronto, ON, Canada. ^15^Dept. of Pharmacy and Biotechnology, University of Bologna, Bologna, Italy. ^16^McKusick-Nathans Institute of Genetic Medicine, Johns Hopkins University, Baltimore, MD, USA. ^17^Dept. of Psychiatry, University of Oxford and Warneford Hospital, Oxford, UK. ^18^Mental Health and Addictions Research Unit, University of British Colombia, Vancouver, BC, Canada. ^19^Paediatric Neurodisability, King’s Health Partners, Kings College London, London, UK. ^20^Stella Maris Institute for Child and Adolescent Neuropsychiatr, Pisa, Italy. ^21^Dept. of Molecular and Human Genetics, Baylor College of Medicine, Houston, TX, USA. ^22^Dept. of Psychiatry and Behavioral Sciences, University of Washington, Seattle, WA, USA. ^23^INSERM U1130, Paris, France. ^24^CNRS UMR 8246, Paris, France. ^25^Sorbonne Universités, UPMC Univ Paris 6, Neuroscience Paris Seine, Paris, France. ^26^Dept. of Child and Adolescent Psychiatry, Psychosomatics and Psychotherapy, JW Goethe University Frankfurt, Frankfurt am Main, Germany. ^27^Dept. of Women’s and Children’s Health, Center of Neurodevelopmental Disorders, Karolinska Institutet, Stockholm, Sweden. ^28^Child and Adolescent Psychiatry, Center for Psychiatry Research, Stockholm County Council, Stockholm, Sweden. ^29^Institute of Psychiatry, Kings College London, London, UK.
^30^South London & Maudsley Biomedical Research Centre for Mental Health, London, UK.
^31^FondaMental Foundation, Créteil, France. ^32^Human Genetics and Cognitive Functions Unit, Institut Pasteur, Paris, France. ^33^Centre National de la Recherche Scientifique URA 2182 Institut Pasteur, Paris, France. ^34^University Paris Diderot, Sorbonne Paris Cité, Paris, France. ^35^Dept. of Psychiatry, David Geffen School of Medicine at University of California Los Angeles, Los Angeles, CA, USA. ^36^Dept. of Human Genetics, David Geffen School of Medicine at University of California Los Angeles, Los Angeles, CA, USA. ^37^Temple Street Children’s University Hospital, Dublin, Ireland. ^38^Academic Centre on Rare Diseases, University College Dublin, Dublin, Ireland. ^39^Dept. of Medical and Molecular Genetics and Program in Medical Neuroscience, Indiana University School of Medicine, Indianapolis, IN, USA. ^40^Instituto Nacional de Saúde Dr. Ricardo Jorge, Lisboa, Portugal. ^41^Center for Biodiversity, Functional and Integrative Genomics, Campus da FCUL, Lisbon, Portugal. ^42^The John P. Hussman Institute for Human Genomics, University of Miami, Miami, FL, USA. ^43^Duke Center for Autism and Brain Developments, Duke University School of Medicine, Durham, NC, USA. ^44^Duke Institute for Brain Sciences, Duke University School of Medicine, Durham, NC, USA. ^45^Dept. of Psychiatry, Brain Center Rudolf Magnus, University Medical Center Utrecht, Utrecht, The Netherlands. ^46^Seaver Autism Center for Research and Treatment, Icahn School of Medicine at Mount Sinai, New York, NY, USA. ^47^Dept. of Psychiatry, Icahn School of Medicine at Mount Sinai, New York, NY, USA. ^48^Dept. of Child and Adolescent Psychiatry, Robert Debré Hospital, Assistance Publique – Hôpitaux de Paris, Paris, France. ^49^University Clinic of Pediatrics and Institute for Biomedical Imaging and Life Science, Faculty of Medicine, University of Coimbra, Coimbra, Portugal. ^50^Centre for Medical Genetics, Our Lady's Hospital Crumlin, Dublin, Ireland. ^51^Programs on Neurogenetics, Yale University School of Medicine, New Haven, CT, USA. ^52^Dept. of Mental Health, Johns Hopkins Bloomberg School of Public Health, Baltimore, MD, USA. ^53^Memorial University of Newfoundland, St. John’s, NL, Canada. ^54^Division of Child and Adolescent Psychiatry, Dept.t of Psychiatry, Miller School of Medicine, University of Miami, Miami, FL, USA. ^55^Dept. of Psychiatry and Institute for Development and Disability, Oregon Health & Science University, Portland, OR, USA. ^56^Gillberg Neuropsychiatry Centre, University of Gothenburg, Gothenburg, Sweden. ^57^Manchester Academic Health Sciences Centre, Manchester, UK. ^58^Institute of Brain, Behaviour, and Mental Health, University of Manchester, Manchester, UK. ^59^Institute for Juvenile Research, Dept. of Psychiatry, University of Illinois at Chicago, Chicago, IL, USA. ^60^Dept. of Molecular Physiology & Biophysics, Vanderbilt University, Nashville, TN, USA. ^61^Dept. of Public Health Sciences, School of Medicine, University of California Davis, Davis, CA, USA. ^62^Karolinska Institutet, Solna, Sweden. ^63^Institute of Translational Neuroscience and Dept. of Psychiatry, University of Minnesota, Minneapolis, MN, USA. ^64^Division of Molecular Genome Analysis and Working Group Cancer Genome Research, Deutsches Krebsforschungszentrum, Heidelberg, Germany. ^65^Friedman Brain Institute, Icahn School of Medicine at Mount Sinai, New York, NY, USA. ^66^The Mindich Child Health and Development Institute, Icahn School of Medicine at Mount Sinai, New York, NY, USA. ^67^Institutes of Neuroscience and Health and Society, Newcastle University, Newcastle Upon Tyne, UK. ^68^INSERM U955, Paris, France. ^69^Faculté de Médecine, Université Paris Est, Créteil, France. ^70^Dept. of Psychiatry, Henri Mondor-Albert Chenevier Hospital, Assistance Publique – Hôpitaux de Paris, Créteil, France. ^71^Chief Scientific Officer, Geisinger Health System, Danville, PA, USA. ^72^Autism & Developmental Medicine Institute, Geisinger Health System, Danville, PA, USA. ^73^Dept. of Pediatrics, Keck School of Medicine, University of Southern California, Los Angeles, California, USA. ^74^Dept. of Psychiatry, Weill Cornell Medical College, Cornell University, New York, NY, USA. ^75^Center for Autism Research and Treatment, Semel Institute, David Geffen School of Medicine at University of California Los Angeles, Los Angeles, CA, USA. ^76^ Program in Neurogenetics, Dept. of Neurology, David Geffen School of Medicine, University of California, Los Angeles, Los Angeles, CA, USA. ^77^Center for Neurobehavioral Genetics, Semel Institute, David Geffen School of Medicine, University of California, Los Angeles, Los Angeles, CA, USA. ^78^National Childrens Research Centre, Our Lady's Hospital Crumlin, Dublin, Ireland. ^79^Yale Center for Genomic Analysis, Yale University School of Medicine, New Haven, CT, USA. ^80^Dept. of Pediatrics and Human Genetics, University of Michigan, Ann Arbor, MI, USA. ^81^Dept. of Pediatrics, Vanderbilt University, Nashville, TN, USA. ^82^Dept. of Psychiatry, University of Utah, Salt Lake City, UT, USA. ^83^Wellcome Trust Centre for Human Genetics, Oxford University, Oxford, UK. ^84^Tufts University, Boston, MA, USA. ^85^Dept. of Psychiatry, Yale University School of Medicine, New Haven, CT, USA.
^86^Dept. of Psychiatry and Human Behaviour, Brown University, Providence, RI, USA. ^87^Institute of Psychiatric Research, Dept. of Psychiatry, Indiana University School of Medicine, Indianapolis, IN, USA. ^88^Dept. of Molecular Genetics, University of Toronto, Toronto, ON, Canada. ^89^The Centre for Applied Genomics, The Hospital for Sick Children, Toronto, ON, Canada. ^90^Dalla Lana School of Public Health, Toronto, ON, Canada. ^91^Dept. of Genetics and Genomic Sciences, Icahn School of Medicine at Mount Sinai, New York, NY, USA. ^92^The Icahn Institute for Genomics and Multiscale Biology, Icahn School of Medicine at Mount Sinai, New York, NY, USA. ^93^University of North Carolina, Chapel Hill, NC, USA. ^94^Sanger Institute, Hinxton, UK. ^95^Autism Research Unit, The Hospital for Sick Children, Toronto, ON, Canada. ^96^Dept. of Computational Biology, Carnegie Mellon University, Pittsburgh, PA, USA. ^97^Dept. of Statistics, Carnegie Mellon University, Pittsburgh, PA, USA. ^98^Centre d’Etudes et de Recherches en Psychopathologie, Toulouse University, Toulouse, France. ^99^Montreal Neurological Institute, Dept of Neurology and Neurosurgery, McGill University, Montreal, QC, Canada. ^100^Dept. of Pathology and Laboratory Medicine, University of Pennsylvania, Philadelphia, PA, USA. ^101^McLaughlin Centre, University of Toronto, Toronto, ON, Canada. ^102^Dept. of Psychiatry, Rush University Medical Center, Chicago, IL, USA. ^103^Dept. of Psychiatry, University of Toronto, ON, Canada. ^104^Dept. of Psychiatry and Behavioral Neurosciences, McMasters University, Hamilton, ON, Canada. ^105^Battelle Center for Mathematical Medicine, The Research Institute at Nationwide Children’s Hospital, Columbus, OH, USA. ^106^Division of Genetics, Children's Hospital Boston, Harvard Medical School, Boston, MA, USA. ^107^Program in Genetics and Genomics, Harvard Medical School, Boston, MA, USA.
^108^Howard Hughes Medical Institute, Harvard Medical School, Boston, MA, USA. ^109^Dept. of Pediatrics, Harvard Medical School, Boston, MA, USA. ^110^Dept. of Neurology, Harvard Medical School, Boston, MA, USA. ^111^Dept. of Psychiatry, Carver College of Medicine, Iowa City, IA, USA. ^112^Dept. of Medicine, University of Washington, Seattle, WA, USA.
^113^Dept. of Biostatistics, University of Washington, Seattle, WA, USA. ^114^School of Education, University of Birmingham, Birmingham, UK. ^115^Dept. of Pediatrics, University of Alberta, Edmonton, AB, Canada. ^116^Dept. of Neuroscience, Icahn School of Medicine at Mount Sinai, New York, NY, USA. ^117^The Center for Applied Genomics and Division of Human Genetics, Children’s Hospital of Philadelphia, University of Pennsylvania School of Medicine, Philadelphia, PA, USA. ^118^Dept of Pediatrics, University of Pennsylvania, Philadelphia, PA.USA. ^119^Dept. of Psychiatry, Stanford University, Stanford, CA, USA.
^120^Maine Medical Center Research Institute, Portland, ME, USA. ^121^Center for Human Genetics Research, Vanderbilt University, Nashville, TN, USA.

^*^These authors contributed equally to this work.

^†^These authors contributed equally to this work.

**iii. The Genetic and Environmental Risk for Alzheimer’s disease (GERAD1) Consortium**

*Data used in the preparation of this article were obtained from the Genetic and Environmental Risk for Alzheimer’s disease (GERAD1) Consortium. As such, the investigators within the GERAD1 consortia contributed to the design and implementation of GERAD1 and/or provided data but did not participate in analysis or writing of this report.

**Authors :**

Denise Harold^1^, Rebecca Sims^1^, Amy Gerrish^1^, Jade Chapman^1^, Valentina Escott-Price^1^, Richard Abraham^1^, Paul Hollingworth^1^, Marian Hamshere^1^, Jaspreet Singh Pahwa^1^, Kimberley Dowzell^1^, Amy Williams^1^, Nicola Jones^1^, Charlene Thomas^1^, Alexandra Stretton^1^, Angharad Morgan^1^, Kate Williams^1^, Sarah Taylor^1^,Simon Lovestone^2^, John Powell^2^, Petroula Proitsi^2^, Michelle K Lupton^2^, Carol Brayne^3^, David C. Rubinsztein^4^, Michael Gill^5^, Brian Lawlor^5^, Aoibhinn Lynch^5^, Kevin Morgan^6^, Kristelle Brown^6^, Peter Passmore^7^, David Craig^7^, Bernadette McGuinness^7^, Janet A Johnston^7^, Stephen Todd^7^, Clive Holmes^8^, David Mann^9^, A. David Smith^10^, Seth Love^11^, Patrick G. Kehoe^11^, John Hardy^12^, Rita Guerreiro^13,33^, Andrew Singleton^13^, Simon Mead^14^, Nick Fox^15^, Martin Rossor^15^, John Collinge^14^, Wolfgang Maier^16^, Frank Jessen^16^, Reiner Heun^16^, Britta Schürmann^16,17^, Alfredo Ramirez^16^, Tim Becker^34^_,_ Christine Herold^34^, André Lacour^34^, Dmitriy Drichel^34^, Hendrik van den Bussche^18^, Isabella Heuser^19^, Johannes Kornhuber^20^, Jens Wiltfang^21^, Martin Dichgans^22,23^, Lutz Frölich^24^, Harald Hampel^25^, Michael Hüll^26^, Dan Rujescu^27^, Alison Goate^28^, John S.K. Kauwe^29^, Carlos Cruchaga^28^, Petra Nowotny^28^, John C. Morris^28^, Kevin Mayo^28^, Gill Livingston^30^, Nicholas J. Bass^30^, Hugh Gurling^30^, Andrew McQuillin^30^, Rhian Gwilliam^31^, Panagiotis Deloukas^31^, Markus M. Nöthen^32^, Peter Holmans^1^, Michael ODonovan^1^, Michael J.Owen^1^, Julie Williams^1^.

**Affiliations**

^1^Medical Research Council (MRC) Centre for Neuropsychiatric Genetics and Genomics, Neurosciences and Mental Health Research Institute, Department of Psychological Medicine and Neurology, School of Medicine, Cardiff University, Cardiff, UK. ^2^Kings College London, Institute of Psychiatry, Department of Neuroscience, De Crespigny Park, Denmark Hill, London. ^3^Institute of Public Health, University of Cambridge, Cambridge, UK. ^4^Cambridge Institute for Medical Research, University of Cambridge, Cambridge, UK. ^5^Mercers Institute for Research on Aging, St. James Hospital and Trinity College, Dublin, Ireland. ^6^Institute of Genetics, Queens Medical Centre, University of Nottingham, UK. ^7^Ageing Group, Centre for Public Health, School of Medicine, Dentistry and Biomedical Sciences, Queens University Belfast, UK. ^8^Division of Clinical Neurosciences, School of Medicine, University of Southampton, Southampton, UK. ^9^Clinical Neuroscience Research Group, Greater Manchester Neurosciences Centre, University of Manchester, Salford, UK. ^10^Oxford Project to Investigate Memory and Ageing (OPTIMA), University of Oxford, Department of Pharmacology, Mansfield Road, Oxford, UK. ^11^University of Bristol Institute of Clinical Neurosciences, School of Clinical Sciences, Frenchay Hospital, Bristol, UK. ^12^Department of Molecular Neuroscience and Reta Lilla Weston Laboratories, Institute of Neurology, UCL, London, UK. ^13^Laboratory of Neurogenetics, National Institute on Aging, National Institutes of Health, Bethesda, Maryland, United States of America. ^14^MRC Prion Unit, Department of Neurodegenerative Disease, UCL Institute of Neurology, London, UK. ^15^Dementia Research Centre, Department of Neurodegenerative Diseases, University College London, Institute of Neurology, London, UK. ^16^Department of Psychiatry, University of Bonn, Sigmund-Freud-Straβe 25, 53105 Bonn, Germany. ^17^Institute for Molecular Psychiatry, University of Bonn, Bonn, Germany. ^18^Institute of Primary Medical Care, University Medical Center Hamburg-Eppendorf, Germany. ^19^Department of Psychiatry, Charité Berlin, Germany. ^20^Department of Psychiatry, Friedrich-Alexander-University Erlangen-Nürnberg, Germany. ^21^Department of Psychiatry and Psychotherapy, University Medical Center (UMG), Georg-August-University, Göttingen, Germany. ^22^Institute for Stroke and Dementia Reserach, Klinikum der Universität München, Marchioninistr. 15, 81377, Munich, Germany. ^23^Department of Neurology, Klinikum der Universität München, Marchioninistr. 15, 81377, Munich, Germany. ^24^Central Institute of Mental Health, Medical Faculty Mannheim, University of Heidelberg, Germany. ^25^Institute for Memory and Alzheimers Disease & INSERM, Sorbonne Universites, Pierre and Marie Curie University, Paris ; Institute for Brain and Spinal Cord Disorders (ICM), Department of Neurology, Hospital of Pitié-Salpétrière. ^26^Centre for Geriatric Medicine and Section of Gerontopsychiatry and Neuropsychology, Medical School, University of Freiburg, Germany. ^27^Department of Psychiatry, University of Halle, Halle, Germany. ^28^Departments of Psychiatry, Neurology and Genetics, Washington University School of Medicine, St Louis, MO 63110, US. ^29^Department of Biology, Brigham Young University, Provo, UT, 84602, USA. ^30^Department of Mental Health Sciences, University College London, UK. ^31^The Wellcome Trust Sanger Institute, Wellcome Trust Genome Campus, Hinxton, Cambridge, UK. ^32^Department of Genomics, Life & Brain Center, University of Bonn, Bonn, Germany. ^33^Department of Molecular Neuroscience, Institute of Neurology, University College London, Queen Square, London WC1N 3BG, UK. ^34^Deutsches Zentrum für Neurodegenerative Erkrankungen (DZNE), Bonn.

## The International Consortium for Blood Pressure (ICBP)

**Authors:**

Georg B. Ehret1,2,3*, Patricia B. Munroe4*#, Kenneth M. Rice5*, Murielle Bochud2*, Andrew D. Johnson6,7*, Daniel I. Chasman8,9*, Albert V. Smith10,11*, Martin D. Tobin12, Germaine C. Verwoert13,14,15, Shih-Jen Hwang6,16,7, Vasyl Pihur1, Peter Vollenweider17, Paul F. O'Reilly18, Najaf Amin13, Jennifer L Bragg-Gresham19, Alexander Teumer20, Nicole L. Glazer21, Lenore Launer22, Jing Hua Zhao23, Yurii Aulchenko13, Simon Heath24, Siim Sõber25, Afshin Parsa26, Jian'an Luan23, Pankaj Arora27, Abbas Dehghan13,14,15, Feng Zhang28, Gavin Lucas29, Andrew A. Hicks30, Anne U. Jackson31, John F Peden32, Toshiko Tanaka33, Sarah H. Wild34, Igor Rudan35,36, Wilmar Igl37, Yuri Milaneschi33, Alex N. Parker38, Cristiano Fava39,40, John C. Chambers18,41, Ervin R. Fox42, Meena Kumari43, Min Jin Go44, Pim van der Harst45, Wen Hong Linda Kao46, Marketa Sjögren39, D. G. Vinay47, Myriam Alexander48, Yasuharu Tabara49, Sue Shaw-Hawkins4, Peter H. Whincup50, Yongmei Liu51, Gang Shi52, Johanna Kuusisto53, Bamidele Tayo54, Mark Seielstad55,56, Xueling Sim57, Khanh-Dung Hoang Nguyen1, Terho Lehtimäki58, Giuseppe Matullo59,60, Ying Wu61, Tom R. Gaunt62, N. Charlotte Onland-Moret63,64, Matthew N. Cooper65, Carl G.P. Platou66, Elin Org25, Rebecca Hardy67, Santosh Dahgam68, Jutta Palmen69, Veronique Vitart70, Peter S. Braund71,72, Tatiana Kuznetsova73, Cuno S.P.M. Uiterwaal63, Adebowale Adeyemo74, Walter Palmas75, Harry Campbell35, Barbara Ludwig76, Maciej Tomaszewski71,72, Ioanna Tzoulaki77,78, Nicholette D. Palmer79, CARDIoGRAM consortium80, CKDGen Consortium80, KidneyGen Consortium80, EchoGen consortium80, CHARGE-HF consortium80, Thor Aspelund10,11, Melissa Garcia22, Yen-Pei C. Chang26, Jeffrey R. O'Connell26, Nanette I. Steinle26, Diederick E. Grobbee63, Dan E. Arking1, Sharon L. Kardia81, Alanna C. Morrison82, Dena Hernandez83, Samer Najjar84,85, Wendy L. McArdle86, David Hadley50,87, Morris J. Brown88, John M. Connell89, Aroon D. Hingorani90, Ian N.M. Day62, Debbie A. Lawlor62, John P. Beilby91,92, Robert W. Lawrence65, Robert Clarke93, Rory Collins93, Jemma C Hopewell93, Halit Ongen32, Albert W. Dreisbach42, Yali Li94, J H. Young95, Joshua C. Bis21, Mika Kähönen96, Jorma Viikari97, Linda S. Adair98, Nanette R. Lee99, Ming-Huei Chen100, Matthias Olden101,102, Cristian Pattaro30, Judith A. Hoffman Bolton103, Anna Köttgen104,103, Sven Bergmann105,106, Vincent Mooser107, Nish Chaturvedi108, Timothy M. Frayling109, Muhammad Islam110, Tazeen H. Jafar110, Jeanette Erdmann111, Smita R. Kulkarni112, Stefan R. Bornstein76, Jürgen Grässler76, Leif Groop113,114, Benjamin F. Voight115, Johannes Kettunen116,126, Philip Howard117, Andrew Taylor43, Simonetta Guarrera60, Fulvio Ricceri59,60, Valur Emilsson118, Andrew Plump118, Inês Barroso119,120, Kay-Tee Khaw48, Alan B. Weder121, Steven C. Hunt122, Yan V. Sun81, Richard N. Bergman123, Francis S. Collins124, Lori L. Bonnycastle124, Laura J. Scott31, Heather M. Stringham31, Leena Peltonen119,125,126,127, Markus Perola125, Erkki Vartiainen125, Stefan-Martin Brand128,129, Jan A. Staessen73, Thomas J. Wang6,130, Paul R. Burton12,72, Maria Soler Artigas12, Yanbin Dong131, Harold Snieder132,131, Xiaoling Wang131, Haidong Zhu131, Kurt K. Lohman133, Megan E. Rudock51, Susan R Heckbert134,135, Nicholas L Smith134,136,135, Kerri L Wiggins137, Ayo Doumatey74, Daniel Shriner74, Gudrun Veldre25,138, Margus Viigimaa139,140, Sanjay Kinra141, Dorairajan Prabhakaran142, Vikal Tripathy142, Carl D. Langefeld79, Annika Rosengren143, Dag S. Thelle144, Anna Maria Corsi145, Andrew Singleton83, Terrence Forrester146, Gina Hilton1, Colin A. McKenzie146, Tunde Salako147, Naoharu Iwai148, Yoshikuni Kita149, Toshio Ogihara150, Takayoshi Ohkubo149,151, Tomonori Okamura148, Hirotsugu Ueshima152, Satoshi Umemura153, Susana Eyheramendy154, Thomas Meitinger155,156, H.-Erich Wichmann157,158,159, Yoon Shin Cho44, Hyung-Lae Kim44, Jong-Young Lee44, James Scott160, Joban S. Sehmi160,41, Weihua Zhang18, Bo Hedblad39, Peter Nilsson39, George Davey Smith62, Andrew Wong67, Narisu Narisu124, Alena Stančáková53, Leslie J. Raffel161, Jie Yao161, Sekar Kathiresan162,27, Chris O'Donnell163,27,9, Stephen M. Schwartz134, M. Arfan Ikram13,15, W. T. Longstreth Jr.164, Thomas H. Mosley165, Sudha Seshadri166, Nick R.G. Shrine12, Louise V. Wain12, Mario A. Morken124, Amy J. Swift124, Jaana Laitinen167, Inga Prokopenko51,168, Paavo Zitting169, Jackie A. Cooper69, Steve E. Humphries69, John Danesh48, Asif Rasheed170, Anuj Goel32, Anders Hamsten171, Hugh Watkins32, Stephan J.L. Bakker172, Wiek H. van Gilst45, Charles S. Janipalli47, K. Radha Mani47, Chittaranjan S. Yajnik112, Albert Hofman13, Francesco U.S. Mattace-Raso13,14, Ben A. Oostra173, Ayse Demirkan13, Aaron Isaacs13, Fernando Rivadeneira13,14, Edward G Lakatta174, Marco Orru175,176, Angelo Scuteri174, Mika Ala-Korpela177,178,179, Antti J Kangas177, Leo-Pekka Lyytikäinen58, Pasi Soininen177,178, Taru Tukiainen180,181,177, Peter Würtz177,18,180, Rick Twee-Hee Ong56,57,182, Marcus Dörr183, Heyo K. Kroemer184, Uwe Völker20, Henry Völzke185, Pilar Galan186, Serge Hercberg186, Mark Lathrop24, Diana Zelenika24, Panos Deloukas119, Massimo Mangino28, Tim D. Spector28, Guangju Zhai28, James F. Meschia187, Michael A. Nalls83, Pankaj Sharma188, Janos Terzic189, M. J. Kranthi Kumar47, Matthew Denniff71, Ewa Zukowska-Szczechowska190, Lynne E. Wagenknecht79, F. Gerald R. Fowkes191, Fadi J. Charchar192, Peter E.H. Schwarz193, Caroline Hayward70, Xiuqing Guo161, Charles Rotimi74, Michiel L. Bots63, Eva Brand194, Nilesh J. Samani71,72, Ozren Polasek195, Philippa J. Talmud69, Fredrik Nyberg68,196, Diana Kuh67, Maris Laan25, Kristian Hveem66, Lyle J. Palmer197,198, Yvonne T. van der Schouw63, Juan P. Casas199, Karen L. Mohlke61, Paolo Vineis200,60, Olli Raitakari201, Santhi K. Ganesh202, Tien Y. Wong203,204, E Shyong Tai205,57,206, Richard S. Cooper54, Markku Laakso53, Dabeeru C. Rao207, Tamara B. Harris22, Richard W. Morris208, Anna F. Dominiczak209, Mika Kivimaki210, Michael G. Marmot210, Tetsuro Miki49, Danish Saleheen170,48, Giriraj R. Chandak47, Josef Coresh211, Gerjan Navis212, Veikko Salomaa125, Bok-Ghee Han44, Xiaofeng Zhu94, Jaspal S. Kooner160,41, Olle Melander39, Paul M Ridker8,213,9, Stefania Bandinelli214, Ulf B. Gyllensten37, Alan F. Wright70, James F. Wilson34, Luigi Ferrucci33, Martin Farrall32, Jaakko Tuomilehto215,216,217,218, Peter P. Pramstaller30,219, Roberto Elosua29,220, Nicole Soranzo119,28, Eric J.G. Sijbrands13,14, David Altshuler221,115, Ruth J.F. Loos23, Alan R. Shuldiner26,222, Christian Gieger157, Pierre Meneton223, Andre G. Uitterlinden13,14,15, Nicholas J. Wareham23, Vilmundur Gudnason10,11, Jerome I. Rotter161, Rainer Rettig224, Manuela Uda175, David P. Strachan50, Jacqueline C.M. Witteman13,15, Anna-Liisa Hartikainen225, Jacques S. Beckmann105,226, Eric Boerwinkle227, Ramachandran S. Vasan6,228, Michael Boehnke31, Martin G. Larson6,229, Marjo-Riitta Järvelin18,230,231,232,233, Bruce M. Psaty21,135*, Gonçalo R Abecasis19*, Aravinda Chakravarti1*#, Paul Elliott18,233*, Cornelia M. van Duijn13,234*, Christopher Newton-Cheh27,115*#, Daniel Levy6,16,7*#, Mark J. Caulfield4*#, Toby Johnson4*

**Affiliations**

1. Center for Complex Disease Genomics, McKusick-Nathans Institute of Genetic Medicine, Johns Hopkins University School of Medicine, Baltimore, MD 21205, USA 2. Institute of Social and Preventive Medicine (IUMSP), Centre Hospitalier Universitaire Vaudois and University of Lausanne, Bugnon 17, 1005 Lausanne, Switzerland 3. Cardiology, Department of Specialties of Internal Medicine, Geneva University Hospital, Rue Gabrielle-Perret-Gentil 4, 1211 Geneva 14, Switzerland 4. Clinical Pharmacology and The Genome Centre, William Harvey Research Institute, Barts and The London School of Medicine and Dentistry, Queen Mary University of London, London EC1M 6BQ, UK 5. Department of Biostatistics, University of Washington, Seattle, WA, USA 6. Framingham Heart Study, Framingham, MA, USA 7. National Heart Lung, and Blood Institute, Bethesda, MD, USA 8. Division of Preventive Medicine, Brigham and Women's Hospital, 900 Commonwealth Avenue East, Boston MA 02215, USA 9. Harvard Medical School, Boston, MA, USA 10. Icelandic Heart Association, Kopavogur, Iceland 11. University of Iceland, Reykajvik, Iceland 12. Department of Health Sciences, University of Leicester, University Rd, Leicester LE1 7RH, UK 13. Department of Epidemiology, Erasmus Medical Center, PO Box 2040, 3000 CA, Rotterdam, The Netherlands 14. Department of Internal Medicine, Erasmus Medical Center, Rotterdam, The Netherlands 15. Netherlands Consortium for Healthy Aging (NCHA), Netherland Genome Initiative (NGI), The Netherlands 16. Center for Population Studies, National Heart Lung, and Blood Institute, Bethesda, MD, USA 17. Department of Internal Medicine, Centre Hospitalier Universitaire Vaudois, 1011 Lausanne, Switzerland 18. Department of Epidemiology and Biostatistics, School of Public Health, Imperial College London, Norfolk Place, London W2 1PG, UK 19. Center for Statistical Genetics, Department of Biostatistics, University of Michigan School of Public Health, Ann Arbor, MI 48103, USA 20. Interfaculty Institute for Genetics and Functional Genomics, Ernst-Moritz-Arndt-University Greifswald, 17487 Greifswald, Germany 21. Cardiovascular Health Research Unit, Departments of Medicine, Epidemiology and Health Services, University of Washington, Seattle, WA, USA 22. Laboratory of Epidemiology, Demography, Biometry, National Institute on Aging, National Institutes of Health, Bethesda, Maryland 20892, USA 23. MRC Epidemiology Unit, Institute of Metabolic Science, Cambridge CB2 0QQ, UK 24. Centre National de Génotypage, Commissariat à L'Energie Atomique, Institut de Génomique, Evry, France 25. Institute of Molecular and Cell Biology, University of Tartu, Riia 23, Tartu 51010, Estonia 26. University of Maryland School of Medicine, Baltimore, MD, USA, 21201, USA 27. Center for Human Genetic Research, Cardiovascular Research Center, Massachusetts General Hospital, Boston, Massachusetts, 02114, USA 28. Department of Twin Research & Genetic Epidemiology, King's College London, UK 29. Cardiovascular Epidemiology and Genetics, Institut Municipal d'Investigacio Medica, Barcelona Biomedical Research Park, 88 Doctor Aiguader, 08003 Barcelona, Spain 30. Institute of Genetic Medicine, European Academy Bozen/Bolzano (EURAC), Viale Druso 1, 39100 Bolzano, Italy - Affiliated Institute of the University of Lübeck, Germany 31. Department of Biostatistics, Center for Statistical Genetics, University of Michigan, Ann Arbor, Michigan, 48109, USA 32. Department of Cardiovascular Medicine, The Wellcome Trust Centre for Human Genetics, University of Oxford, Oxford, OX3 7BN, UK 33. Clinical Research Branch, National Institute on Aging, Baltimore MD 21250, USA 34. Centre for Population Health Sciences, University of Edinburgh, EH89AG, UK 35. Centre for Population Health Sciences and Institute of Genetics and Molecular Medicine, College of Medicine and Vet Medicine, University of Edinburgh, EH8 9AG, UK 36. Croatian Centre for Global Health, University of Split, Croatia. 37. Department of Genetics and Pathology, Rudbeck Laboratory, Uppsala University, SE-751 85 Uppsala, Sweden 38. Amgen, 1 Kendall Square, Building 100, Cambridge, MA 02139, USA 39. Department of Clinical Sciences, Lund University, Malmö, Sweden 40. Department of Medicine, University of Verona, Italy 41. Ealing Hospital, London, UB1 3HJ, UK 42. Department of Medicine, University of Mississippi Medical Center, USA 43. Genetic Epidemiology Group, Epidemiology and Public Health, UCL, London, WC1E 6BT, UK 44. Center for Genome Science, National Institute of Health, Seoul, Korea 45. Department of Cardiology, University Medical Center Groningen, University of Groningen, The Netherlands 46. Departments of Epidemiology and Medicine, Johns Hopkins University, Baltimore MD, USA 47. Centre for Cellular and Molecular Biology (CCMB), Council of Scientific and Industrial Research (CSIR), Uppal Road, Hyderabad 500 007, India 48. Department of Public Health and Primary Care, University of Cambridge, CB1 8RN, UK 49. Department of Basic Medical Research and Education, and Department of Geriatric Medicine, Ehime University Graduate School of Medicine, Toon, 791-0295, Japan 50. Division of Community Health Sciences, St George's University of London, London, SW17 0RE, UK 51. Epidemiology & Prevention, Division of Public Health Sciences, Wake Forest University School of Medicine, Winston-Salem, NC 27157, USA 52. Division of Biostatistics and Department of Genetics, School of Medicine, Washington University in St. Louis, Saint Louis, Missouri 63110, USA 53. Department of Medicine, University of Eastern Finland and Kuopio University Hospital, 70210 Kuopio, Finland 54. Department of Preventive Medicine and Epidemiology, Loyola University Medical School, Maywood, IL, USA 55. Department of Laboratory Medicine & Institute of Human Genetics, University of California San Francisco, 513 Parnassus Ave. San Francisco CA 94143, USA 56. Genome Institute of Singapore, Agency for Science, Technology and Research, Singapore, 138672, Singapore 57. Centre for Molecular Epidemiology, Yong Loo Lin School of Medicine, National University of Singapore, Singapore, 117597, Singapore 58. Department of Clinical Chemistry, University of Tampere and Tampere University Hospital, Tampere, 33521, Finland 59. Department of Genetics, Biology and Biochemistry, University of Torino, Via Santena 19, 10126, Torino, Italy 60. Human Genetics Foundation (HUGEF), Via Nizza 52, 10126, Torino, Italy 61. Department of Genetics, University of North Carolina, Chapel Hill, NC, 27599, USA 62. MRC Centre for Causal Analyses in Translational Epidemiology, School of Social & Community Medicine, University of Bristol, Bristol BS8 2BN, UK 63. Julius Center for Health Sciences and Primary Care, University Medical Center Utrecht, Heidelberglaan 100, 3508 GA Utrecht, The Netherlands 64. Complex Genetics Section, Department of Medical Genetics - DBG, University Medical Center Utrecht, 3508 GA Utrecht, The Netherlands 65. Centre for Genetic Epidemiology and Biostatistics, University of Western Australia, Crawley, WA, Australia 66. HUNT Research Centre, Department of Public Health and General Practice, Norwegian University of Science and Technology, 7600 Levanger, Norway 67. MRC Unit for Lifelong Health & Ageing, London, WC1B 5JU, UK 68. Occupational and Environmental Medicine, Department of Public Health and Community Medicine, Institute of Medicine, Sahlgrenska Academy, University of Gothenburg, 40530 Gothenburg, Sweden 69. Centre for Cardiovascular Genetics, University College London, London WC1E 6JF, UK 70. MRC Human Genetics Unit and Institute of Genetics and Molecular Medicine, Edinburgh, EH2, UK 71. Department of Cardiovascular Sciences, University of Leicester, Glenfield Hospital, Leicester, LE3 9QP, UK 72. Leicester NIHR Biomedical Research Unit in Cardiovascular Disease, Glenfield Hospital, Leicester, LE3 9QP, UK; 73. Studies Coordinating Centre, Division of Hypertension and Cardiac Rehabilitation, Department of Cardiovascular Diseases, University of Leuven, Campus Sint Rafaël, Kapucijnenvoer 35, Block D, Box 7001, 3000 Leuven, Belgium; 74. Center for Research on Genomics and Global Health, National Human Genome Research Institute, Bethesda, MD 20892, USA 75. Columbia University, NY, USA 76. Department of Medicine III, Medical Faculty Carl Gustav Carus at the Technical University of Dresden, 01307 Dresden, Germany 77. Epidemiology and Biostatistics, School of Public Health, Imperial College, London, W2 1PG, UK 78. Clinical and Molecular Epidemiology Unit, Department of Hygiene and Epidemiology, University of Ioannina School of Medicine, Ioannina, Greece 79. Wake Forest University Health Sciences, Winston-Salem, NC 27157, USA 80. A list of consortium members is supplied in the S Materials 81. Department of Epidemiology, School of Public Health, University of Michigan, Ann Arbor, MI 48109, USA 82. Division of Epidemiology, Human Genetics and Environmental Sciences, School of Public Health, University of Texas at Houston Health Science Center, 12 Herman Pressler, Suite 453E, Houston, TX 77030, USA 83. Laboratory of Neurogenetics, National Institute on Aging, Bethesda, MD 20892, USA 84. Laboratory of Cardiovascular Science, Intramural Research Program, National Institute on Aging, NIH, Baltimore, Maryland, USA 85. Washington Hospital Center, Division of Cardiology, Washington DC, USA 86. ALSPAC Laboratory, University of Bristol, Bristol, BS8 2BN, UK 87. Pediatric Epidemiology Center, University of South Florida, Tampa, FL, USA 88. Clinical Pharmacology Unit, University of Cambridge, Addenbrookes Hospital, Hills Road, Cambridge CB2 2QQ, UK 89. University of Dundee, Ninewells Hospital &Medical School, Dundee, DD1 9SY, UK 90. Genetic Epidemiology Group, Department of Epidemiology and Public Health, UCL, London WC1E 6BT, UK 91. Pathology and Laboratory Medicine, University of Western Australia, Crawley, WA, Australia; 92. Molecular Genetics, PathWest Laboratory Medicine, Nedlands, WA, Australia; 93. Clinical Trial Service Unit and Epidemiological Studies Unit, University of Oxford, Oxford, OX3 7LF, UK 94. Department of Epidemiology and Biostatistics, Case Western Reserve University, 2103 Cornell Road, Cleveland, OH 44106, USA; 95. Department of Medicine, Johns Hopkins University, Baltimore, USA; 96. Department of Clinical Physiology, University of Tampere and Tampere University Hospital, Tampere, 33521, Finland; 97. Department of Medicine, University of Turku and Turku University Hospital, Turku, 20521, Finland; 98. Department of Nutrition, University of North Carolina, Chapel Hill, NC, 27599, USA; 99. Office of Population Studies Foundation, University of San Carlos, Talamban, Cebu City 6000, Philippines; 100. Department of Neurology and Framingham Heart Study, Boston University School of Medicine, Boston, MA, 02118, USA; 101. Department of Internal Medicine II, University Medical Center Regensburg, 93053 Regensburg, Germany; 102. Department of Epidemiology and Preventive Medicine, University Medical Center Regensburg, 93053 Regensburg, Germany; 103. Department of Epidemiology, Johns Hopkins University, Baltimore MD, USA; 104. Renal Division, University Hospital Freiburg, Germany; 105. Département de Génétique Médicale, Université de Lausanne, 1015 Lausanne, Switzerland ; 106. Swiss Institute of Bioinformatics, 1015 Lausanne, Switzerland ; 107. Division of Genetics, GlaxoSmithKline, Philadelphia, Pennsylvania 19101, USA; 108. International Centre for Circulatory Health, National Heart & Lung Institute, Imperial College, London, UK; 109. Genetics of Complex Traits, Peninsula Medical School, University of Exeter, UK; 110. Department of Community Health Sciences & Department of Medicine, Aga Khan University, Karachi, Pakistan; 111. Medizinische Klinik II, Universität zu Lübeck, Lübeck, Germany; 112. Diabetes Unit, KEM Hospital and Research Centre, Rasta Peth, Pune-411011, Maharashtra, India; 113. Department of Clinical Sciences, Diabetes and Endocrinology Research Unit, University Hospital, Malmö, Sweden; 114. Lund University, Malmö 20502, Sweden; 115. Program in Medical and Population Genetics, Broad Institute of Harvard and MIT, Cambridge, Massachusetts, 02139, USA; 116. Department of Chronic Disease Prevention, National Institute for Health and Welfare, FIN-00251 Helsinki, Finland; 117. William Harvey Research Institute, Barts and The London School of Medicine and Dentistry, Queen Mary University of London, London EC1M 6BQ, UK; 118. Merck Research Laboratory, 126 East Lincoln Avenue, Rahway, NJ 07065, USA; 119. Wellcome Trust Sanger Institute, Hinxton, CB10 1SA, UK 120. University of Cambridge Metabolic Research Labs, Institute of Metabolic Science Addenbrooke's Hospital, CB2 OQQ, Cambridge, UK; 121. Division of Cardiovascular Medicine, Department of Internal Medicine, University of Michigan Medical School, Ann Arbor, MI, USA; 122. Cardiovascular Genetics, University of Utah School of Medicine, Salt Lake City, UT, USA; 123. Department of Physiology and Biophysics, Keck School of Medicine, University of Southern California, Los Angeles, California 90033, USA; 124. National Human Genome Research Institute, National Institutes of Health, Bethesda, Maryland 20892,USA; 125. National Institute for Health and Welfare, 00271 Helsinki, Finland; 126. FIMM, Institute for Molecular Medicine, Finland, Biomedicum, P.O. Box 104, 00251 Helsinki, Finland; 127. Broad Institute, Cambridge, Massachusetts 02142, USA; 128. Leibniz-Institute for Arteriosclerosis Research, Department of Molecular Genetics of Cardiovascular Disease, University of Münster, Münster, Germany; 129. Medical Faculty of the Westfalian Wilhelms University Muenster, Department of Molecular Genetics of Cardiovascular Disease, University of Muenster, Muenster, Germany; 130. Division of Cardiology, Massachusetts General Hospital, Boston, MA, USA; 131. Georgia Prevention Institute, Department of Pediatrics, Medical College of Georgia, Augusta, GA, USA; 132. Department of Epidemiology, University Medical Center Groningen, University of Groningen, Groningen, The Netherlands; 133. Department of Biostatical Sciences, Division of Public Health Sciences, Wake Forest University School of Medicine, Winston-Salem, NC 27157, USA; 134. Department of Epidemiology, University of Washington, Seattle, WA, 98195, USA; 135. Group Health Research Institute, Group Health Cooperative, Seattle, WA, USA; 136. Seattle Epidemiologic Research and Information Center, Veterans Health Administration Office of Research & Development, Seattle, WA 98108, USA; 137. Department of Medicine, University of Washington, 98195, USA; 138. Department of Cardiology, University of Tartu, L. Puusepa 8, 51014 Tartu, Estonia; 139. Tallinn University of Technology, Institute of Biomedical Engineering, Ehitajate tee 5, 19086 Tallinn, Estonia; 140. Centre of Cardiology, North Estonia Medical Centre, Sütiste tee 19, 13419 Tallinn, Estonia; 141. Division of Non-communicable disease Epidemiology, The London School of Hygiene and Tropical Medicine London, Keppel Street, London WC1E 7HT, UK; 142. South Asia Network for Chronic Disease, Public Health Foundation of India, C-1/52, SDA, New Delhi 100016, India; 143. Department of Emergency and Cardiovascular Medicine, Institute of Medicine, Sahlgrenska Academy, University of Gothenburg, 41685 Gothenburg, Sweden; 144. Department of Biostatistics, Institute of Basic Medical Sciences, University of Oslo, 0317 Oslo, Norway; 145. Tuscany Regional Health Agency, Florence, Italy 146. Tropical Medicine Research Institute, University of the West Indies, Mona, Kingston, Jamaica; 147. University of Ibadan, Ibadan, Nigeria 148. Department of Genomic Medicine, and Department of Preventive Cardiology, National Cerebral and Cardiovascular Research Center, Suita, 565-8565, Japan; 149. Department of Health Science, Shiga University of Medical Science, Otsu, 520-2192, Japan; 150. Department of Geriatric Medicine, Osaka University Graduate School of Medicine, Suita, 565-0871, Japan; 151. Tohoku University Graduate School of Pharmaceutical Sciences and Medicine, Sendai, 980-8578, Japan; 152. Lifestyle-related Disease Prevention Center, Shiga University of Medical Science, Otsu, 520-2192, Japan; 153. Department of Medical Science and Cardiorenal Medicine, Yokohama City University School of Medicine, Yokohama, 236-0004, Japan; 154. Department of Statistics, Pontificia Universidad Catolica de Chile, Vicuña Mackena 4860, Santiago, Chile; 155. Institute of Human Genetics, Helmholtz Zentrum Munich, German Research Centre for Environmental Health, 85764 Neuherberg, Germany; 156. Institute of Human Genetics, Klinikum rechts der Isar, Technical University of Munich, 81675 Munich, Germany; 157. Institute of Epidemiology, Helmholtz Zentrum Munich, German Research Centre for Environmental Health, 85764 Neuherberg, Germany; 158. Chair of Epidemiology, Institute of Medical Informatics, Biometry and Epidemiology, Ludwig-Maximilians-Universität, 81377 Munich, Germany; 159. Klinikum Grosshadern, 81377 Munich, Germany; 160. National Heart and Lung Institute, Imperial College London, London, UK, W12 0HS, UK; 161. Medical Genetics Institute, Cedars-Sinai Medical Center, Los Angeles, CA, USA; 162. Medical Population Genetics, Broad Institute of Harvard and MIT, 5 Cambridge Center, Cambridge MA 02142, USA; 163. National Heart, Lung and Blood Institute and its Framingham Heart Study, 73 Mount Wayte Ave., Suite #2, Framingham, MA 01702, USA; 164. Department of Neurology and Medicine, University of Washington, Seattle, USA; 165. Department of Medicine (Geriatrics), University of Mississippi Medical Center, Jackson, MS, USA; 166. Department of Neurology, Boston University School of Medicine, USA; 167. Finnish Institute of Occupational Health, Finnish Institute of Occupational Health, Aapistie 1, 90220 Oulu, Finland; 168. Wellcome Trust Centre for Human Genetics, University of Oxford, UK; 169. Lapland Central Hospital, Department of Physiatrics, Box 8041, 96101 Rovaniemi, Finland; 170. Center for Non-Communicable Diseases Karachi, Pakistan; 171. Atherosclerosis Research Unit, Department of Medicine, Karolinska Institute, Stockholm, Sweden; 172. Department of Internal Medicine, University Medical Center Groningen, University of Groningen, The Netherlands; 173. Department of Medical Genetics, Erasmus Medical Center, Rotterdam, The Netherlands; 174. Gerontology Research Center, National Institute on Aging, Baltimore, MD 21224, USA; 175. Istituto di Neurogenetica e Neurofarmacologia, Consiglio Nazionale delle Ricerche, Cittadella Universitaria di Monserrato, Monserrato, Cagliari, Italy; 176. Unita` Operativa Semplice Cardiologia, Divisione di Medicina, Presidio Ospedaliero Santa Barbara, Iglesias, Italy; 177. Computational Medicine Research Group, Institute of Clinical Medicine, University of Oulu and Biocenter Oulu, 90014 University of Oulu, Oulu, Finland; 178. NMR Metabonomics Laboratory, Department of Biosciences, University of Eastern Finland, 70211 Kuopio, Finland; 179. Department of Internal Medicine and Biocenter Oulu, Clinical Research Center, 90014 University of Oulu, Oulu, Finland; 180. Institute for Molecular Medicine Finland FIMM, 00014 University of Helsinki, Helsinki, Finland; 181. Department of Biomedical Engineering and Computational Science, School of Science and Technology, Aalto University, 00076 Aalto, Espoo, Finland; 182. NUS Graduate School for Integrative Sciences & Engineering (NGS) Centre for Life Sciences (CeLS), Singapore, 117456, Singapore; 183. Department of Internal Medicine B, Ernst-Moritz-Arndt-University Greifswald, 17487 Greifswald, Germany; 184. Institute of Pharmacology, Ernst-Moritz-Arndt-University Greifswald, 17487 Greifswald, Germany; 185. Institute for Community Medicine, Ernst-Moritz-Arndt-University Greifswald, 17487 Greifswald, Germany; 186. U557 Institut National de la Santé et de la Recherche Médicale, U1125 Institut National de la Recherche Agronomique, Université Paris 13, Bobigny, France ; 187. Department of Neurology, Mayo Clinic, Jacksonville, FL, USA 188. Imperial College Cerebrovascular Unit (ICCRU), Imperial College, London, W6 8RF, UK 189. Faculty of Medicine, University of Split, Croatia. 190. Department of Internal Medicine, Diabetology, and Nephrology, Medical University of Silesia, 41-800, Zabrze, Poland 191. Public Health Sciences section, Division of Community Health Sciences, University of Edinburgh, Medical School, Teviot Place, Edinburgh, EH8 9AG, UK 192. School of Science and Engineering, University of Ballarat, 3353 Ballarat, Australia 193. Prevention and Care of Diabetes, Department of Medicine III, Medical Faculty Carl Gustav Carus at the Technical University of Dresden, 01307 Dresden, Germany 194. University Hospital Münster, Internal Medicine D, Münster, Germany 195. Department of Medical Statistics, Epidemiology and Medical Informatics, Andrija Stampar School of Public Health, University of Zagreb, Croatia 196. AstraZeneca R&D, 431 83 Mölndal, Sweden 197. Genetic Epidemiology & Biostatistics Platform, Ontario Institute for Cancer Research, Toronto 198. Samuel Lunenfeld Institute for Medical Research, University of Toronto, Canada 199. Faculty of Epidemiology and Population Health, London School of Hygiene and Tropical Medicine, UK 200. Department of Epidemiology and Public Health, Imperial College, Norfolk Place London W2 1PG, UK 201. Research Centre of Applied and Preventive Cardiovascular Medicine, University of Turku and the Department of Clinical Physiology, Turku University Hospital, Turku, 20521, Finland 202. Department of Internal Medicine, Division of Cardiovascular Medicine, University of Michigan Medical Center, Ann Arbor, Michigan, USA 203. Singapore Eye Research Institute, Singapore, 168751, Singapore 204. Department of Ophthalmology, National University of Singapore, Singapore, 119074, Singapore 205. Department of Medicine, Yong Loo Lin School of Medicine, National University of Singapore, Singapore, 119074, Singapore 206. Duke-National University of Singapore Graduate Medical School, Singapore, 169857, Singapore 207. Division of Biostatistics, Washington University School of Medicine, Saint Louis, MO, 63110, USA 208. Department of Primary Care & Population Health, UCL, London, UK, NW3 2PF, UK 209. BHF Glasgow Cardiovascular Research Centre, University of Glasgow, 126 University Place, Glasgow, G12 8TA, UK 210. Epidemiology Public Health, UCL, London, UK, WC1E 6BT, UK 211. Departments of Epidemiology, Biostatistics, and Medicine, Johns Hopkins University, Baltimore MD, USA 212. Division of Nephrology, Department of Internal Medicine, University Medical Center Groningen, University of Groningen, The Netherlands 213. Division of Cardiology, Brigham and Women's Hospital, 900 Commonwealth Avenue East, Boston MA 02215, USA 214. Geriatric Rehabilitation Unit, Azienda Sanitaria Firenze (ASF), Florence, Italy 215. National Institute for Health and Welfare, Diabetes Prevention Unit, 00271 Helsinki, Finland 216. Hjelt Institute, Department of Public Health, University of Helsinki, 00014 Helsinki, Finland 217. South Ostrobothnia Central Hospital, 60220 Seinäjoki, Finland 218. Red RECAVA Grupo RD06/0014/0015, Hospital Universitario La Paz, 28046 Madrid, Spain 219. Department of Neurology, General Central Hospital, 39100 Bolzano, Italy 220. CIBER Epidemiología y Salud Pública, 08003 Barcelona 221. Department of Medicine and Department of Genetics, Harvard Medical School, Boston, Massachusetts 02115, USA 222. Geriatric Research and Education Clinical Center, Veterans Administration Medical Center, Baltimore, MD, USA 223. U872 Institut National de la Santé et de la Recherche Médicale, Centre de Recherche des Cordeliers, Paris, France 224. Institute of Physiology, Ernst-Moritz-Arndt-University Greifswald, 17487 Greifswald, Germany 225. Institute of Clinical Medicine/Obstetrics and Gynecology, University of Oulu, Finland 226. Service of Medical Genetics, Centre Hospitalier Universitaire Vaudois, 1011 Lausanne, Switzerland 227. Human Genetics Center, 1200 Hermann Pressler, Suite E447 Houston, TX 77030, USA 228. Division of Epidemiology and Prevention, Boston University School of Medicine, Boston, MA, USA 229. Department of Mathematics, Boston University, Boston, MA, USA. 230.Institute of Health Sciences, University of Oulu, BOX 5000, 90014 University of Oulu, Finland. 231. Biocenter Oulu, University of Oulu, BOX 5000, 90014 University of Oulu, Finland 232. National Institute for Health and Welfare, Box 310, 90101 Oulu, Finland 233. MRC-HPA Centre for Environment and Health, School of Public Health, Imperial College London, Norfolk Place, London W2 1PG, UK 234. Centre of Medical Systems Biology (CMSB 1-2), NGI Erasmus Medical Center, Rotterdam, The Netherlands.

## The Psoriasis and Psoriatic Arthritis Genetics Consortium

**Name of co-author: affiliation(s):**

James T Elder :

Department of Dermatology, University of Michigan Ann Arbor, MI 48109, USA;

Ann Arbor Veterans Affairs Hospital, Ann Arbor, MI 48105 USA

Philip E Stuart: Department of Dermatology, University of Michigan Ann Arbor, MI 48109, USA

Rajan P Nair: Department of Dermatology, University of Michigan Ann Arbor, MI 48109, USA

Trilokraj Tejasvi: Department of Dermatology, University of Michigan Ann Arbor, MI 48109, USA.

Johann E Gudjonsson: Department of Dermatology, University of Michigan Ann Arbor, MI 48109, USA

John J Voorhees: Department of Dermatology, University of Michigan Ann Arbor, MI 48109, USA.

Lam C Tsoi: Department of Biostatistics, Center for Statistical Genetics, University of Michigan Ann Arbor, MI 48109, USA

Jun Ding: Department of Biostatistics, Center for Statistical Genetics, University of Michigan Ann Arbor, MI 48109, USA

Yanming Li: Department of Biostatistics, Center for Statistical Genetics, University of Michigan Ann Arbor, MI 48109, USA

Hyun M Kang: Department of Biostatistics, Center for Statistical Genetics, University of Michigan Ann Arbor, MI 48109, USA;

Goncalo R Abecasis: Department of Biostatistics, Center for Statistical Genetics, University of Michigan Ann Arbor, MI 48109, USA

Andre Franke: Institute of Clinical Molecular Biology, Christian-Albrechts-University of Kiel, 24105 Kiel, Germany

Eva Ellinghaus: Institute of Clinical Molecular Biology, Christian-Albrechts-University of Kiel, 24105 Kiel, Germany

Stefan Schreiber: Institute of Clinical Molecular Biology, Christian-Albrechts-University of Kiel, 24105 Kiel, Germany

Ulrich Mrowietz: Department of Dermatology, University Hospital, Schleswig-Holstein, Christian-Albrechts-University of Kiel, 24105 Kiel, Germany

Stephan Weidinger: Department of Dermatology, University Hospital, Schleswig-Holstein, Christian-Albrechts-University of Kiel, 24105 Kiel, Germany

Michael Weichenthal ; Department of Dermatology, University Hospital, Schleswig-Holstein, Christian-Albrechts-University of Kiel, 24105 Kiel, Germany

Sören Mucha: Institute of Clinical Molecular Biology, Christian-Albrechts-University of Kiel, 24105 Kiel, Germany

Dafna D Gladman: Department of Medicine, Division of Rheumatology, University of Toronto, Toronto Western Hospital, Toronto, Ontario M5T 2S8, Canada

Fawnda J Pellett: Department of Medicine, Division of Rheumatology, University of Toronto, Toronto Western Hospital, Toronto, Ontario M5T 2S8, Canada

Vinod Chandran: Department of Medicine, Division of Rheumatology, University of Toronto, Toronto Western Hospital, Toronto, Ontario M5T 2S8, Canada

Cheryl F Rosen: Department of Medicine, Division of Dermatology, University of Toronto, Toronto Western Hospital, Toronto, Ontario MST2S8

Proton Rahman: Department of Medicine, Memorial University, St. John’s, Newfoundland A1C 5B8, Canada

Sulev Koks: Department of Physiology, Centre of Translational Medicine and Centre for Translational Genomics, University of Tartu, 50409 Tartu, Estonia

Külli Kingo: Department of Dermatology and Venerology, University of Tartu, 50409 Tartu, Estonia

Tonu Esko: Estonian Genome Center and Center of Translational Genomics; Estonian Biocenter; Institute of Molecular and Cell Biology, University of Tartu, 50409 Tartu, Estonia

Andres Metspalu: Estonian Genome Center and Center of Translational Genomics; Estonian Biocenter; Institute of Molecular and Cell Biology, University of Tartu, 50409 Tartu, Estonia

Peter Gregersen: Robert S. Boas Center for Genomics and Human Genetics, The Feinstein Institute for Medical Research, Manhasset, NY 11030

Andrew Henschel: National Psoriasis Foundation, Portland, OR 97223 USA

Marin Aurand National Psoriasis Foundation, Portland, OR 97223 USA

Bruce Bebo: National Psoriasis Foundation, Portland, OR 97223 USA

H. Erich Wichmann: Institute of Epidemiology I, Helmholtz Centre Munich, German Research Center for Environmental Health, 85764 Neuherberg, Germany; Institute of Medical Informatics, Biometry and Epidemiology, Ludwig-Maximilians-University, 81377 Munich, Germany; Klinikum Grosshadern, 81377 Munich, Germany

Christian Gieger: Institute of Genetic Epidemiology, Helmholtz Centre Munich, German Research Center for Environmental Health, 85764 Neuherberg, Germany

Thomas Illig :Research Unit Molecular Epidemiology, Helmholtz Centre Munich, German Research Center for Environmental Health, 85764 Neuherberg, Germany

Juliane Winkelmann: Department of Neurology, Technische Universität München, Munich, Germany; Institute of Human Genetics, Technische Universität München, Munich, Germany; Institute of Human Genetics, Helmholtz Zentrum Munich, German Research Center for Environmental Health, Munich, Germany

Susanne Moebus: Institute for Medical Informatics, Biometry and Epidemiology (IMIBE), University of Duisburg-Essen, Hufelandstr. 55, 45122 Essen, Germany

Karl-Heinz Jöckel: Institute for Medical Informatics, Biometry and Epidemiology (IMIBE), University of Duisburg-Essen, Hufelandstr. 55, 45122 Essen, Germany

Raimund Erbel: Clinic of Cardiology, West German Heart Centre, University Hospital of Essen, University Duisburg-Essen, Essen, Germany

Markus M. Nöthen: Institute of Human Genetics, University of Bonn, Bonn, Germany; Department of Genomics, Life & Brain Center, University of Bonn, Bonn, Germany

Henry W Lim Henry Ford Hospital, Detroit, Michigan, 48202, USA
