# Supplementary material for: Investigating the Causal Relationship of C-Reactive Protein with 32 Complex Somatic and Psychiatric Outcomes: A Large-Scale Cross-Consortium Mendelian Randomization Study
Source: PLoS Med. 2016 Jun 21;13(6):e1001976. doi: 10.1371/journal.pmed.1001976 (PMC4915710; doi:10.1371/journal.pmed.1001976)
Supplement: S1 Fig — (DOCX) [file pmed.1001976.s003.docx]

Investigating the causal relationship of C-reactive protein with 32 complex somatic and psychiatric outcomes: A large scale cross-consortia Mendelian randomization study.

S1 Figure. GRS P Plots of CRP versus each outcome

Contents: Page

A. Celiac disease 2

B. Inflammatory Bowel Disease (all types) 2

C. Crohn’s Disease 2

D. Ulcerative Colitis 3

E. Psoriasis Vulgaris 3

F. Psoriatic Arthritis 3

G. Psoriasis Cutaneous 4

H. Rheumatoid Arthritis 4

I. Systemic Lupus Erythematosus 4

J. Systemic Sclerosis 5

K. Type I Diabetes 5

L. Knee Osteoarthritis 5

M. Coronary Artery Disease 6

N. Systolic Blood Pressure 6

O. Diastolic Blood Pressure 6

P. Ischemic Stroke (all types) 7

Q. Ischemic Stroke (Cardioembolic Stroke) 7

R. Ischemic Stroke (Large Vessel Disease) 7

S. Ischemic Stroke (Small Vessel Disease) 8

T. Body Mass Index 8

U. Type II Diabetes 8

V. Chronic Kidney Disease 9

W. eGFR for Creatinine 9

X. Serum Albumin Levels 9

Y. Serum Protein Levels 10

Z. Amyotrophic Lateral Sclerosis 10

AA. Alzheimer's Disease 10

AB. Parkinsons's Disease 11

AC. Autism 11

AD. Bipolar Disorder 11

AE. Major Depressive Disorder 12

AF. Schizophrenia 12

For each disease / trait we show the plots for two genetic risk scores ; on the left the GRS*_CRP_* and on the right GRS*_GWAS_*. For every graph, the estimated effects on disease risk (log odds) or trait level (vertical axis) are plotted against estimated effects on the natural log CRP levels (mg/ml) (horizontal axis), for either the GRS*_CRP_* SNPs or GRS*_GWAS_* SNPs that are associated with CRP levels. The grey vertical lines indicate the 95% confidence interval (CI) for each individual SNP. The effect estimate estimate of CRP levels on disease risk or trait level is represented by a red solid line with gradient α. The 95% CI of this α estimate is represented by red dashed lines.

## A. Celiac disease


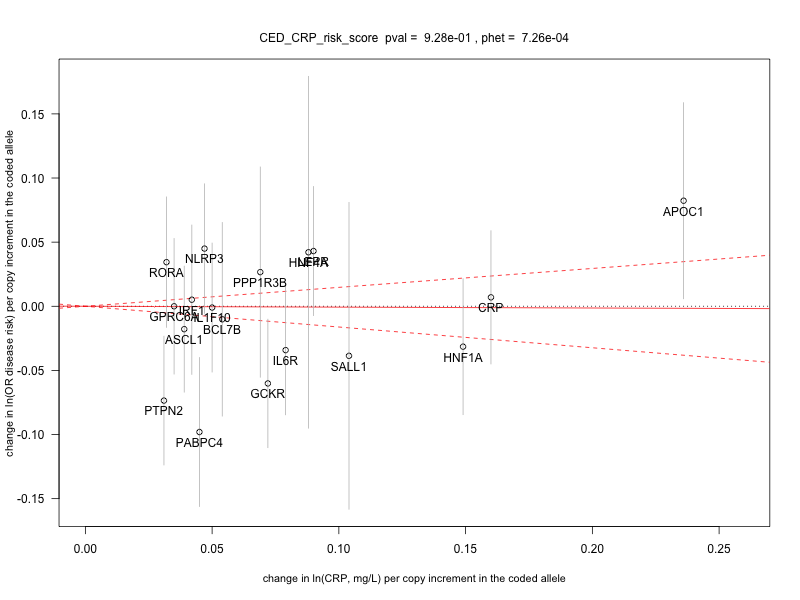

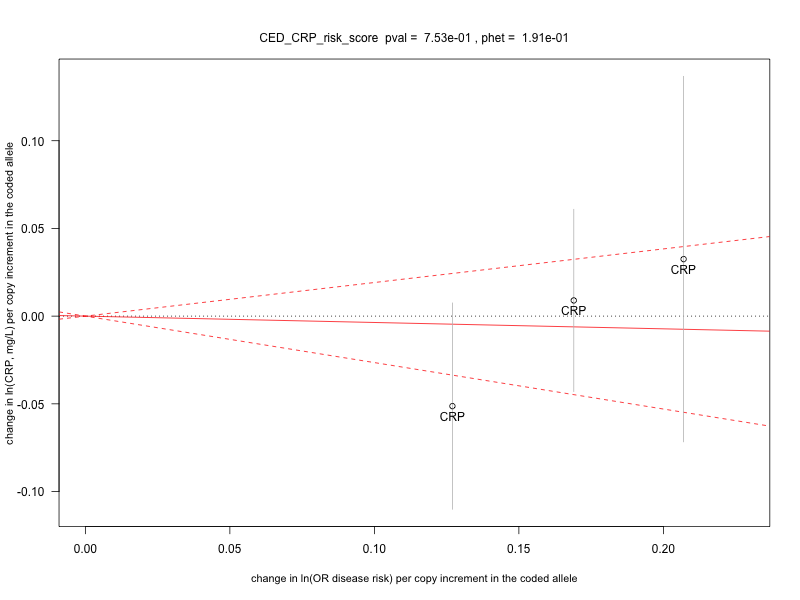


## B. Inflammatory Bowel Disease (all types)


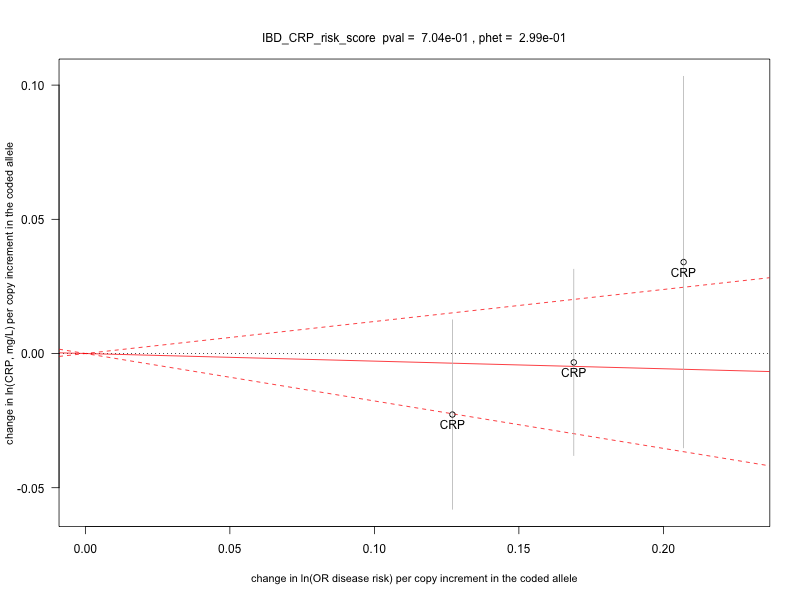

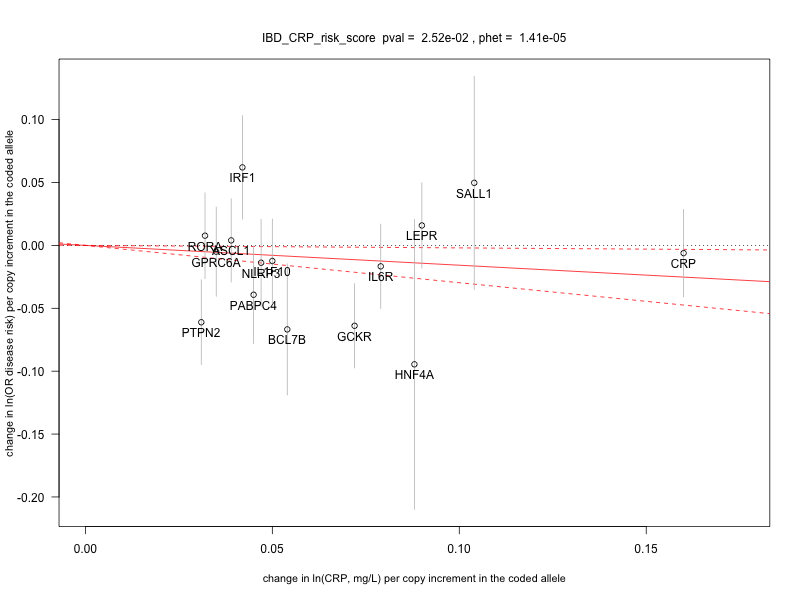


## C. Crohn’s Disease


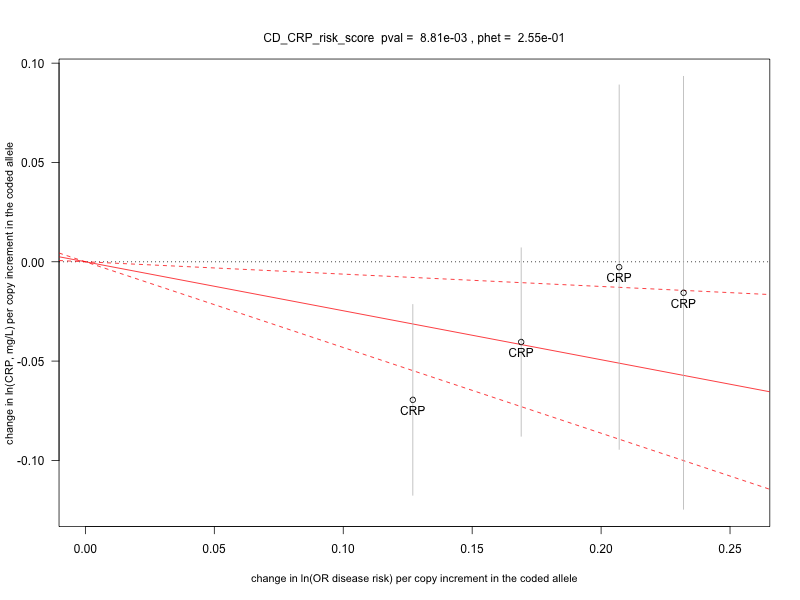

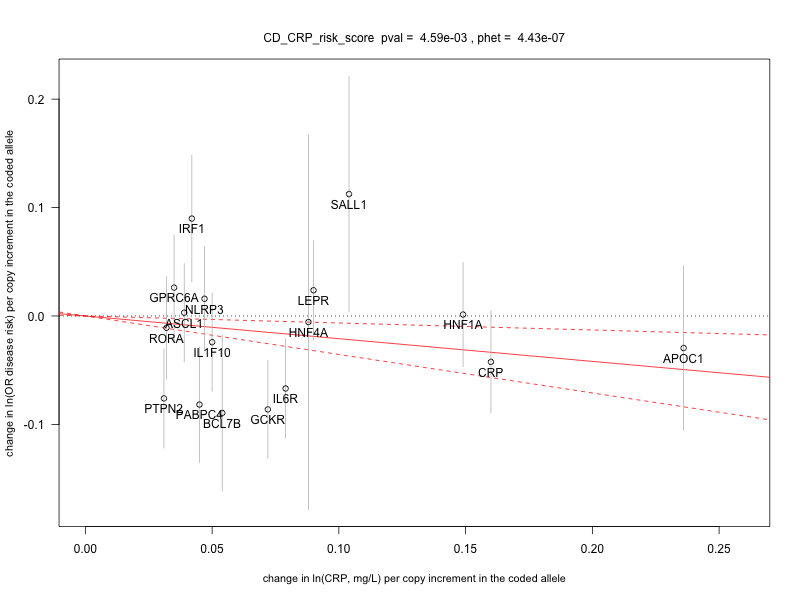


## D. Ulcerative Colitis


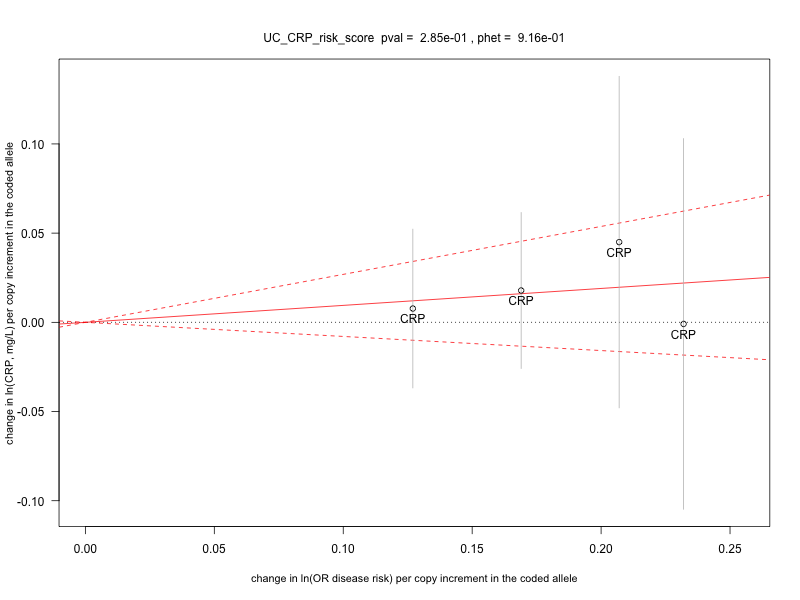

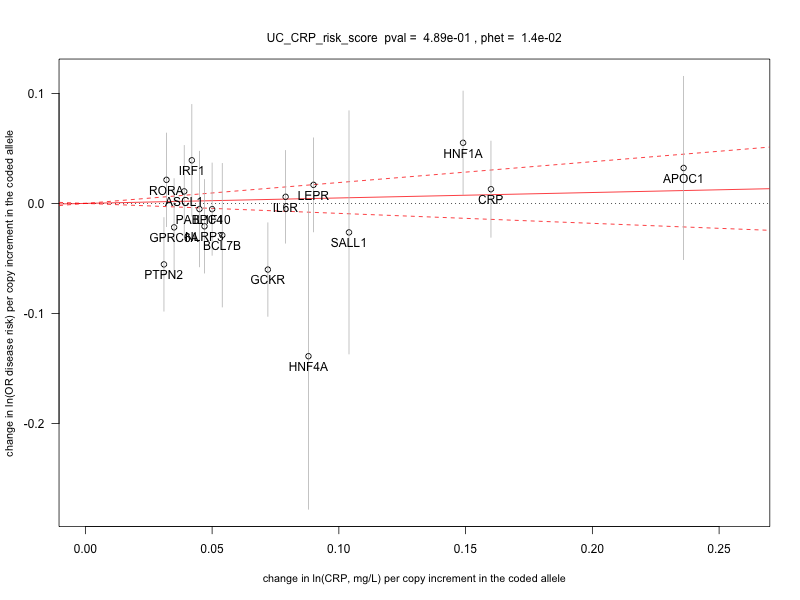


## E. Psoriasis Vulgaris


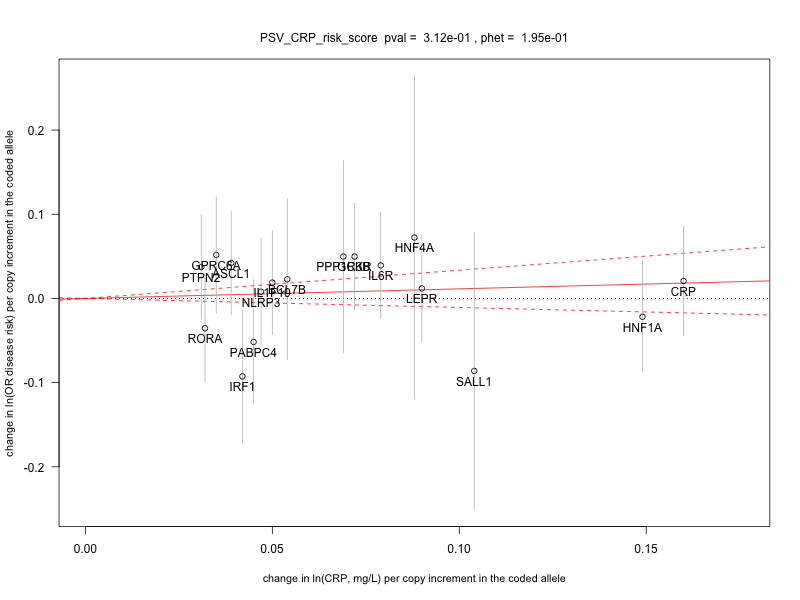

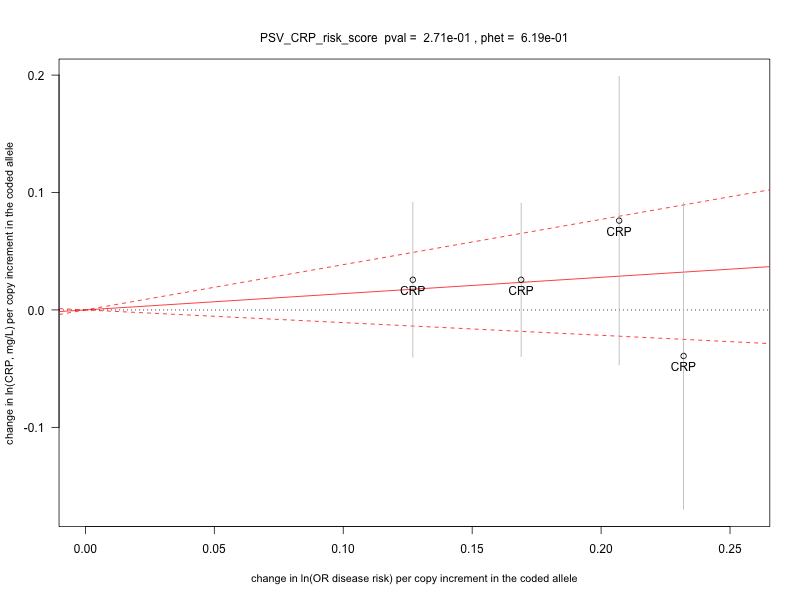


## F. Psoriatic Arthritis


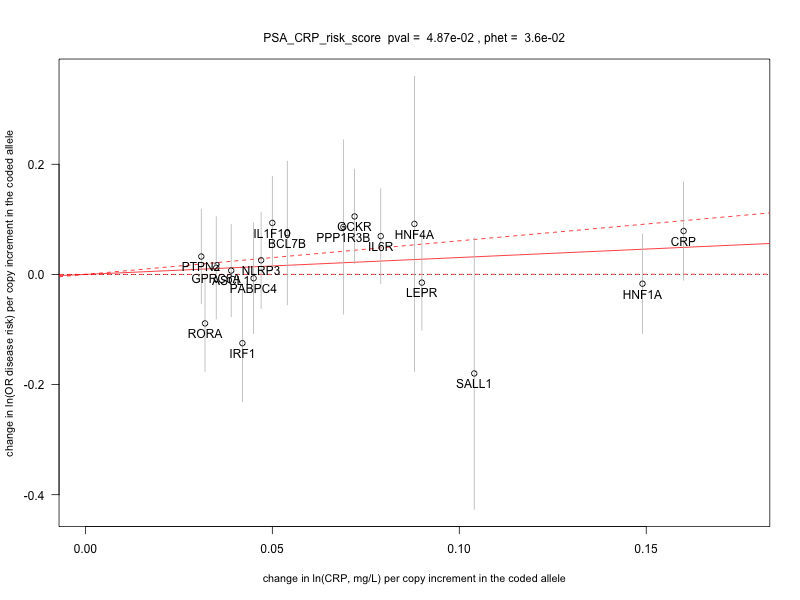

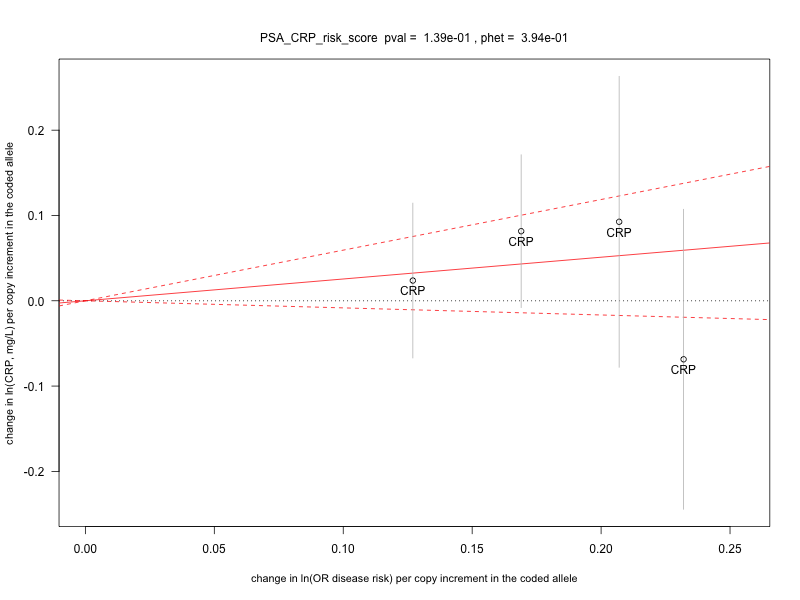


## G. Psoriasis Cutaneous


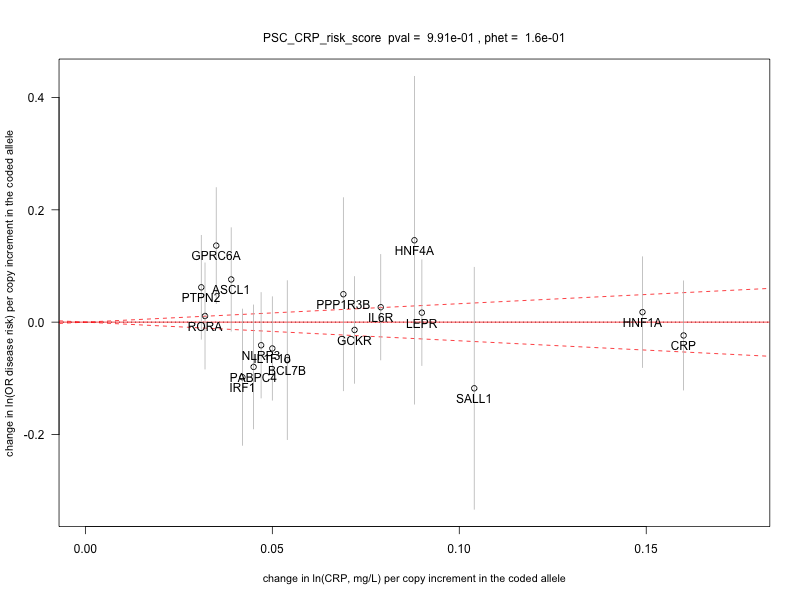

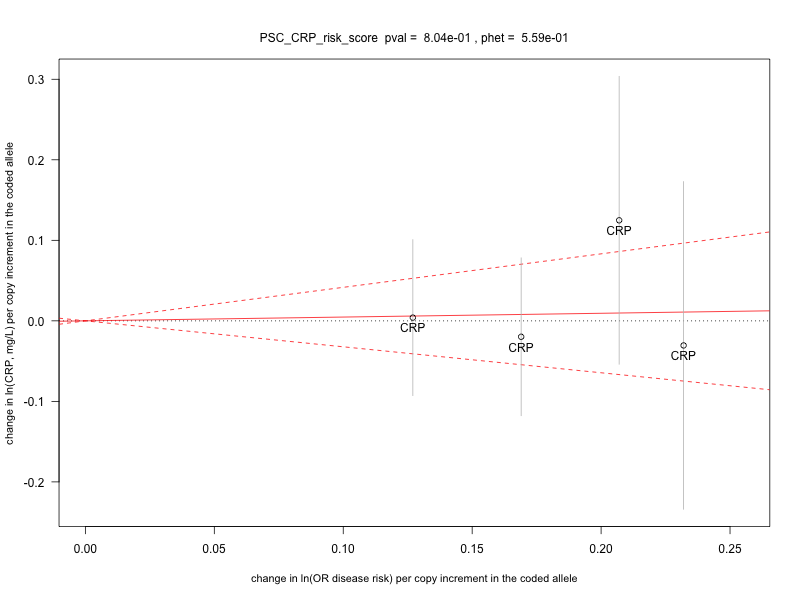


## H. Rheumatoid Arthritis


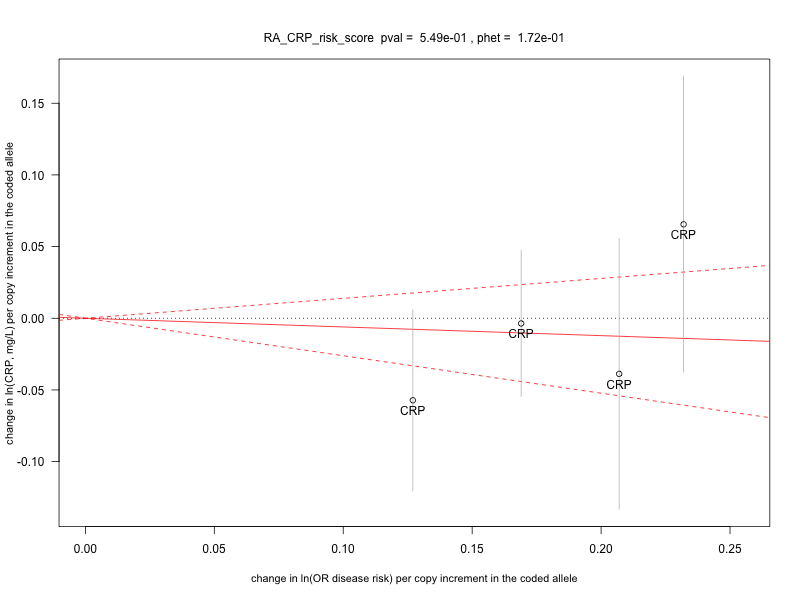

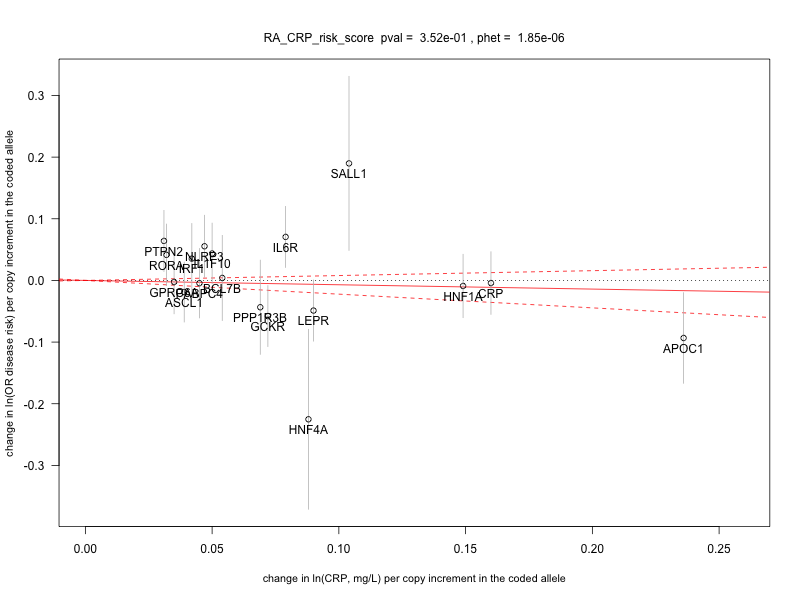


## I. Systemic Lupus Erythematosus


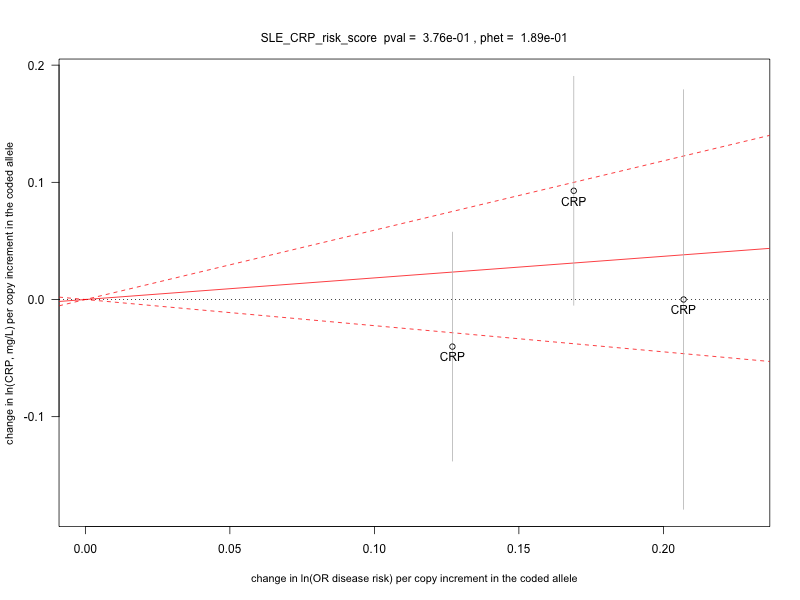

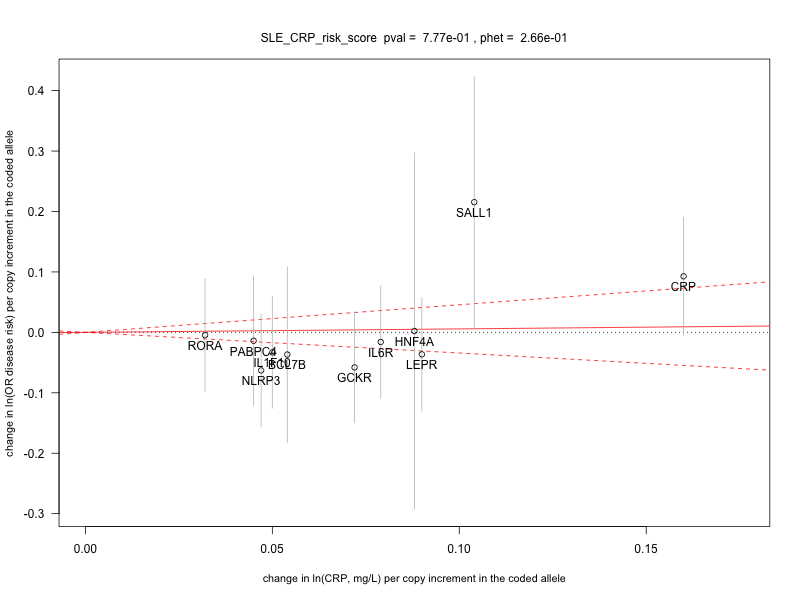


## J. Systemic Sclerosis


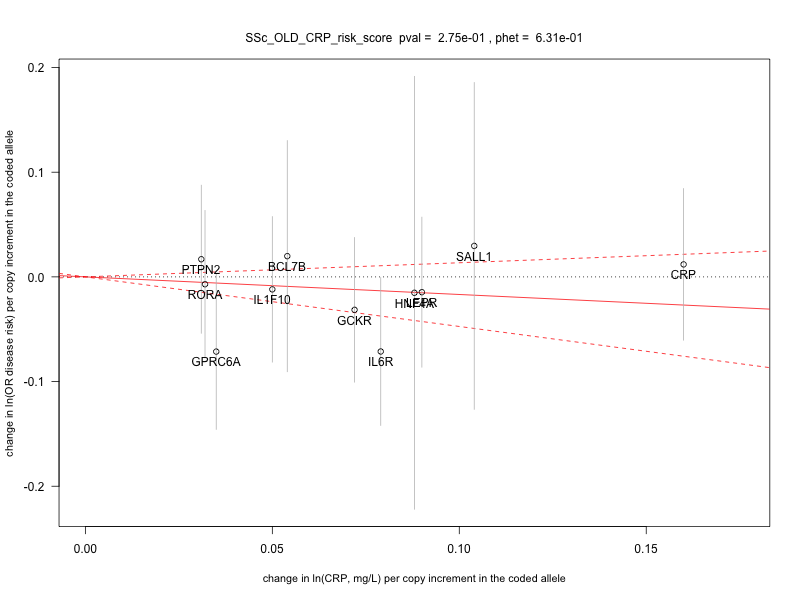

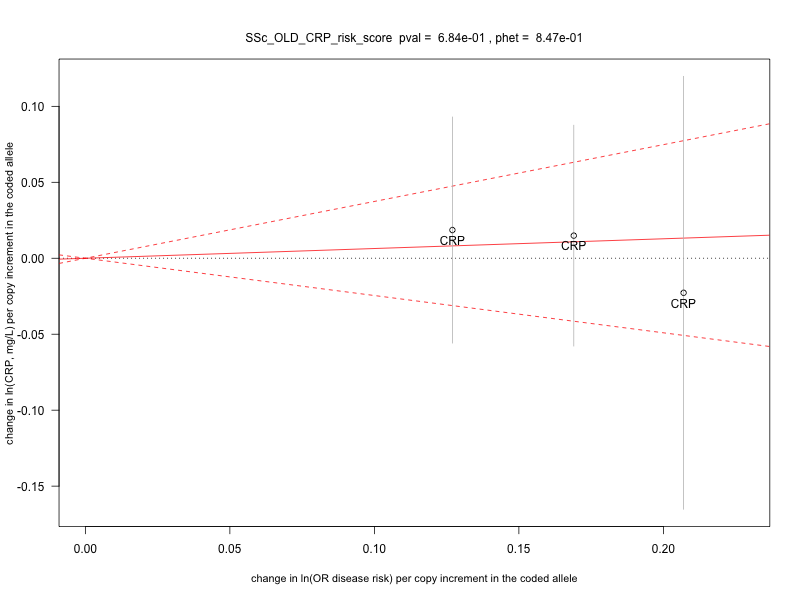


## K. Type I Diabetes


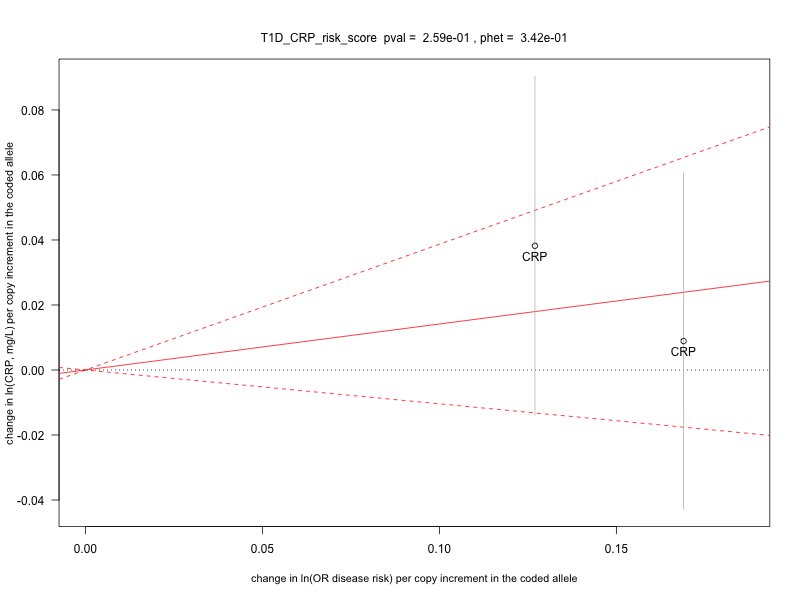

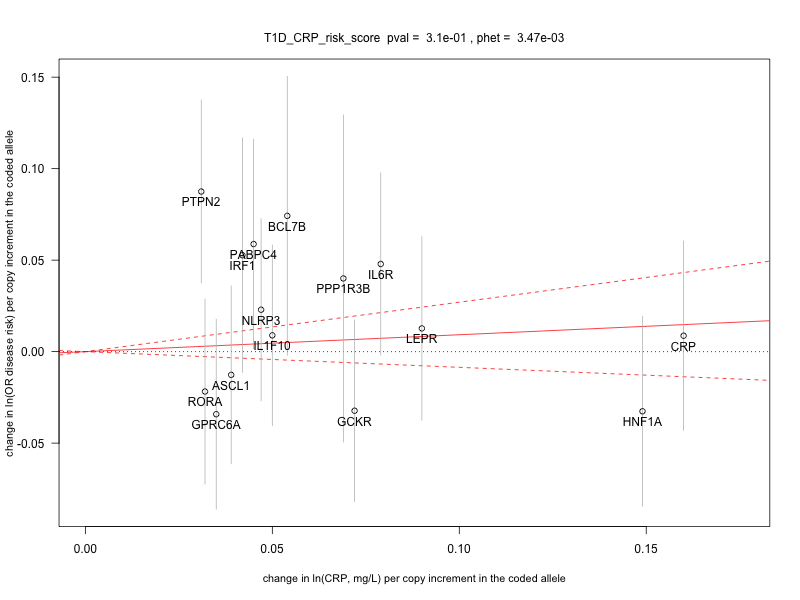


## L. Knee Osteoarthritis


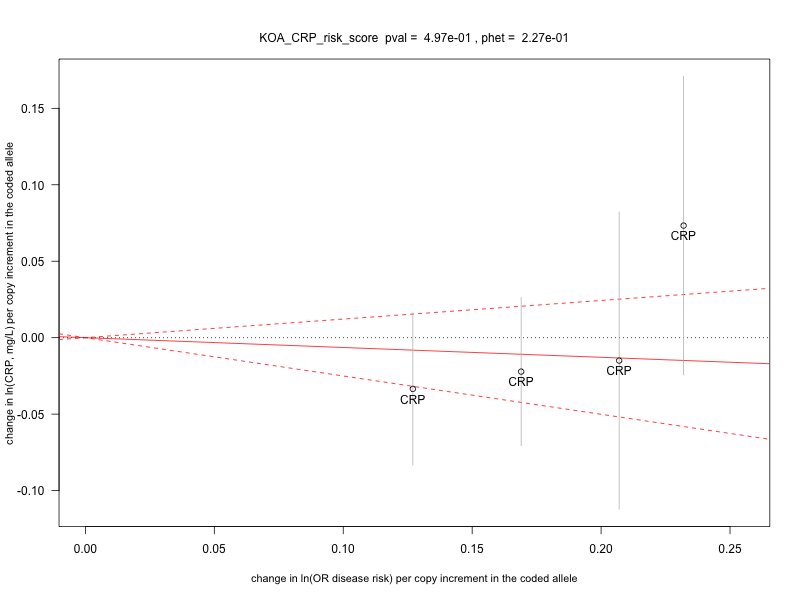

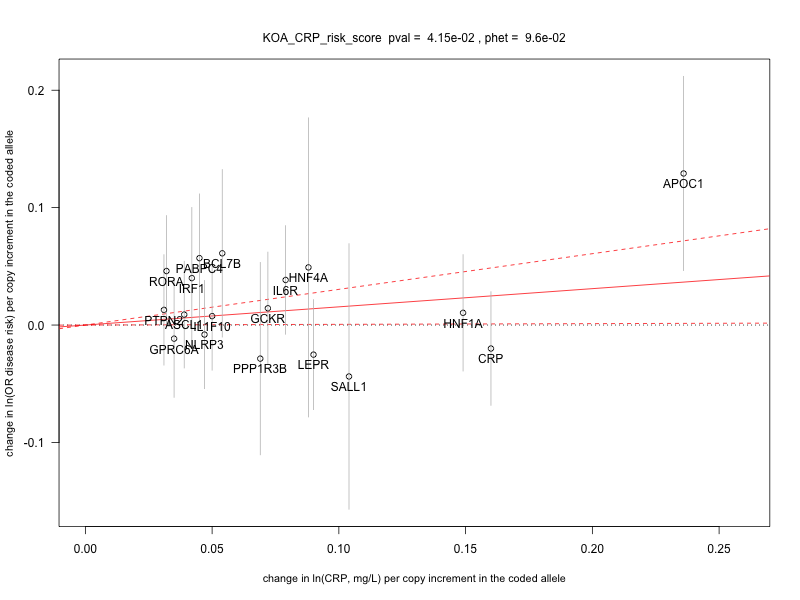


## M. Coronary Artery Disease


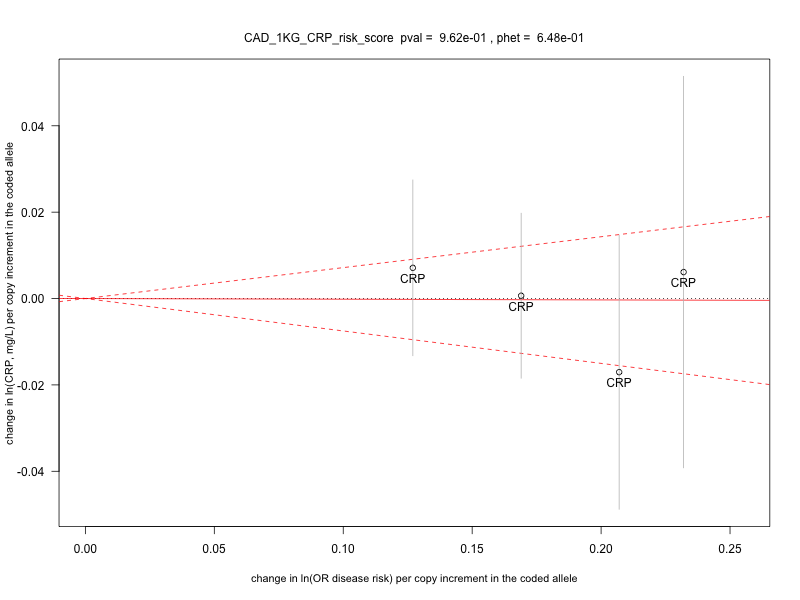

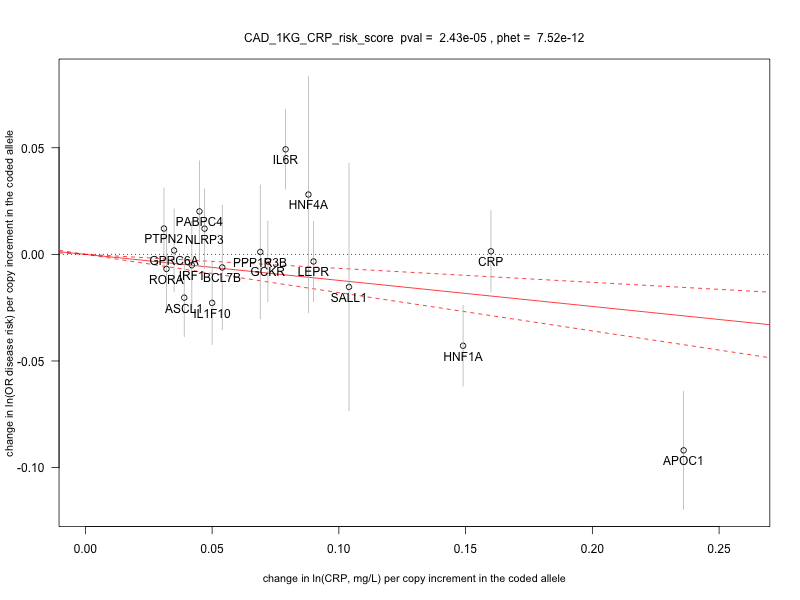


##
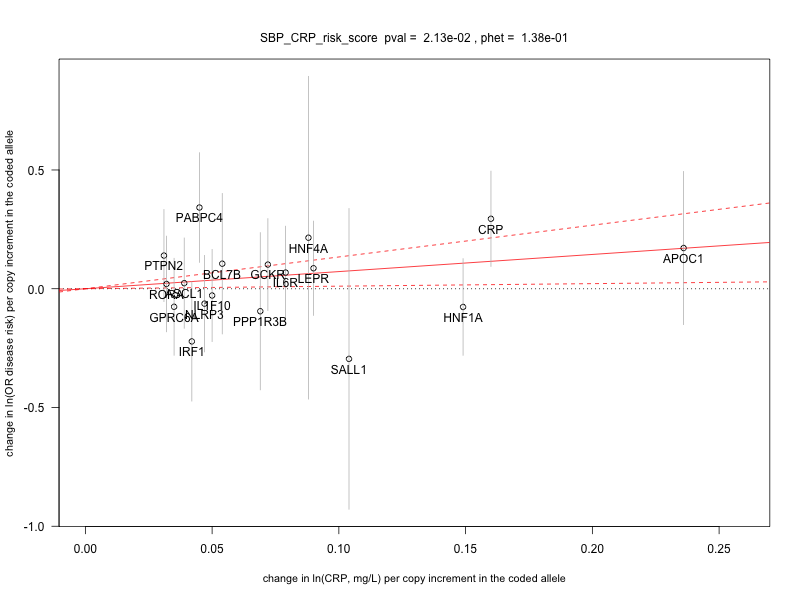

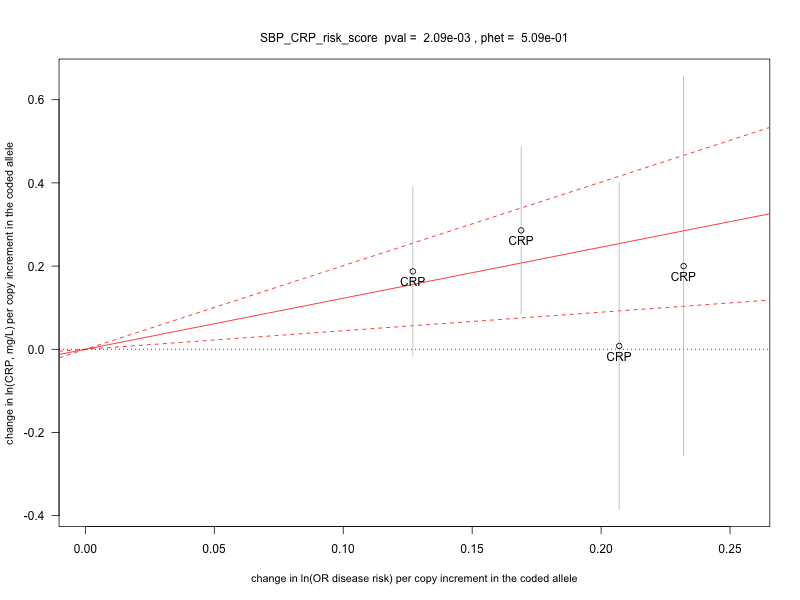
N. Systolic Blood Pressure

## O. Diastolic Blood Pressure


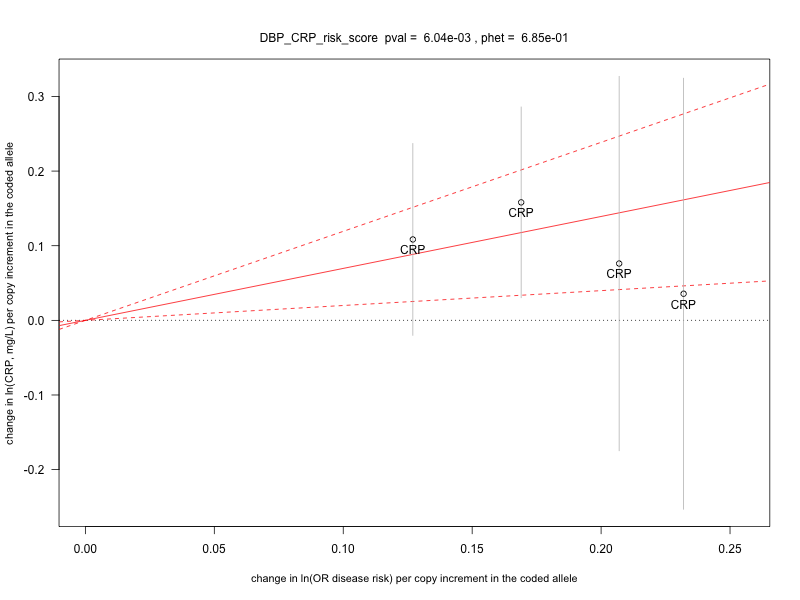

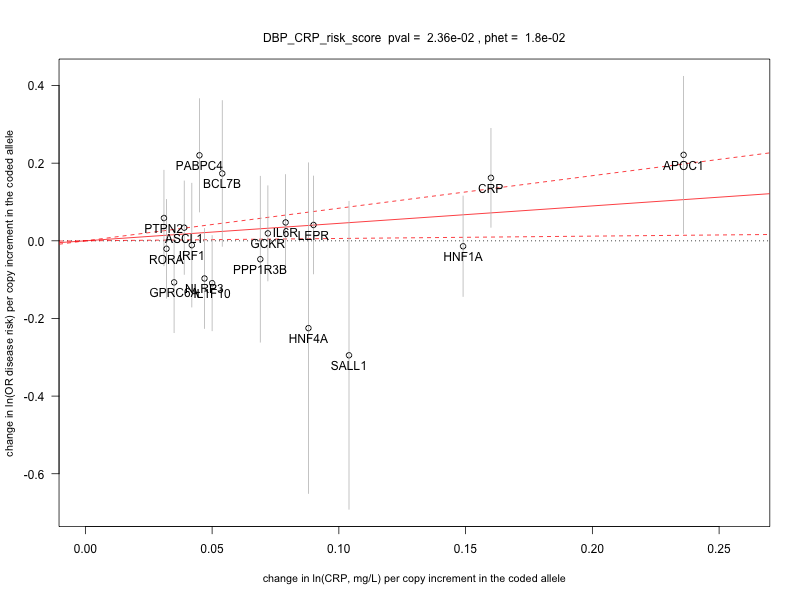


## P. Ischemic Stroke (all types)


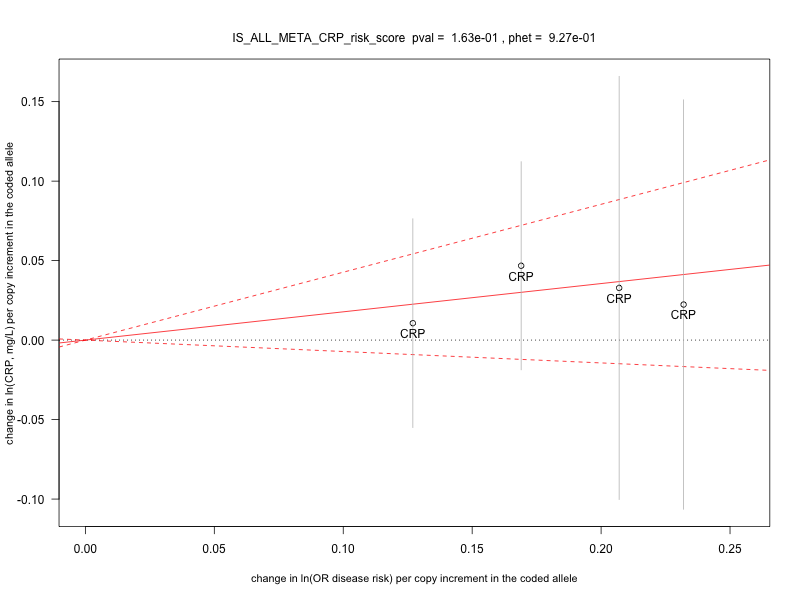

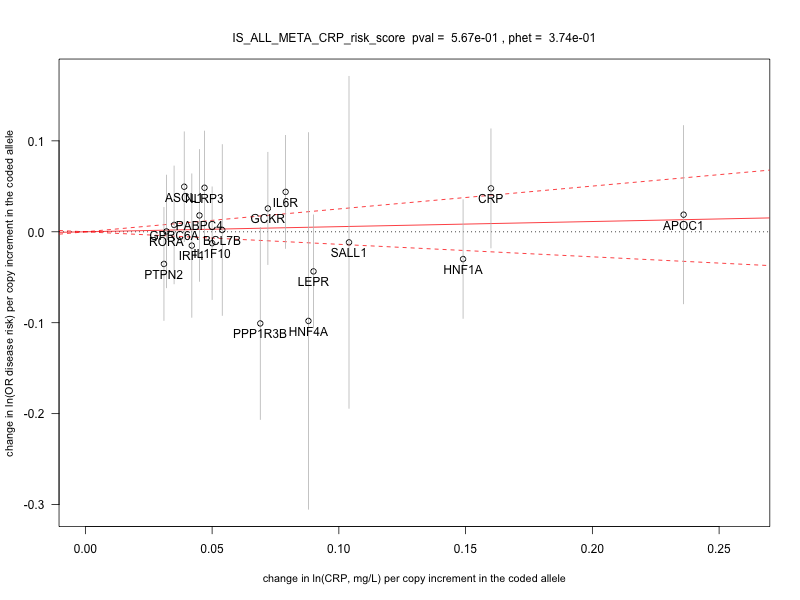


##
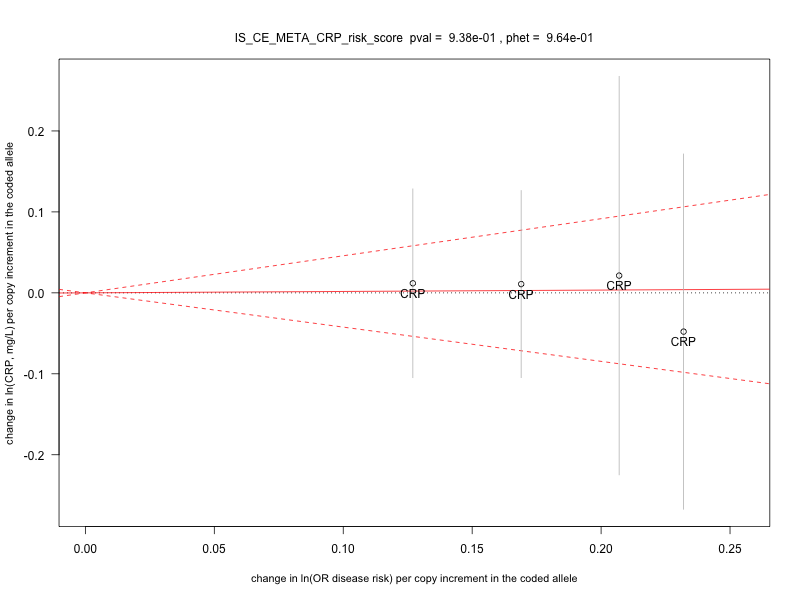

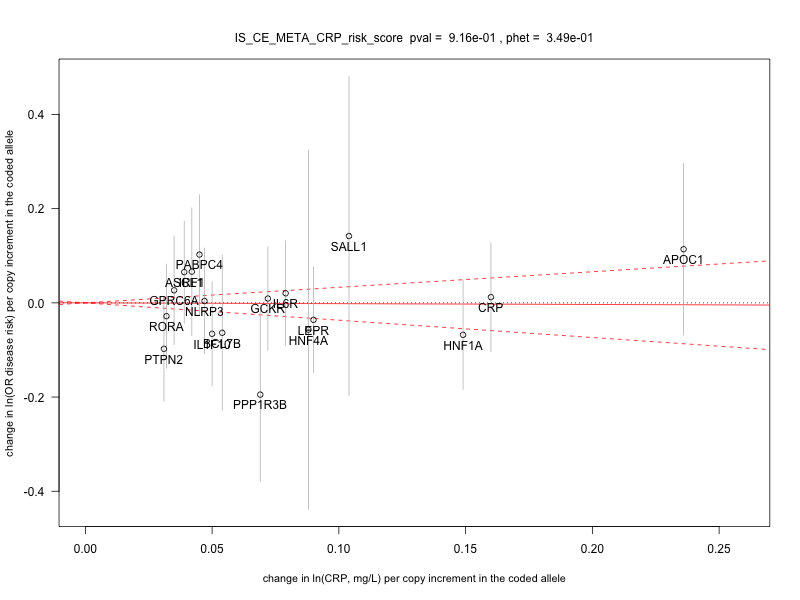
Q. Ischemic Stroke (Cardioembolic Stroke)

## R. Ischemic Stroke (Large Vessel Disease)


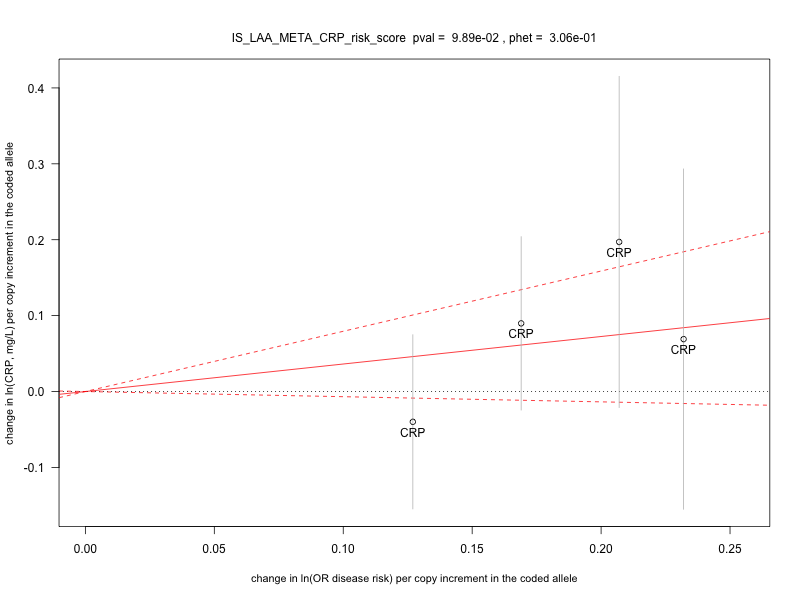

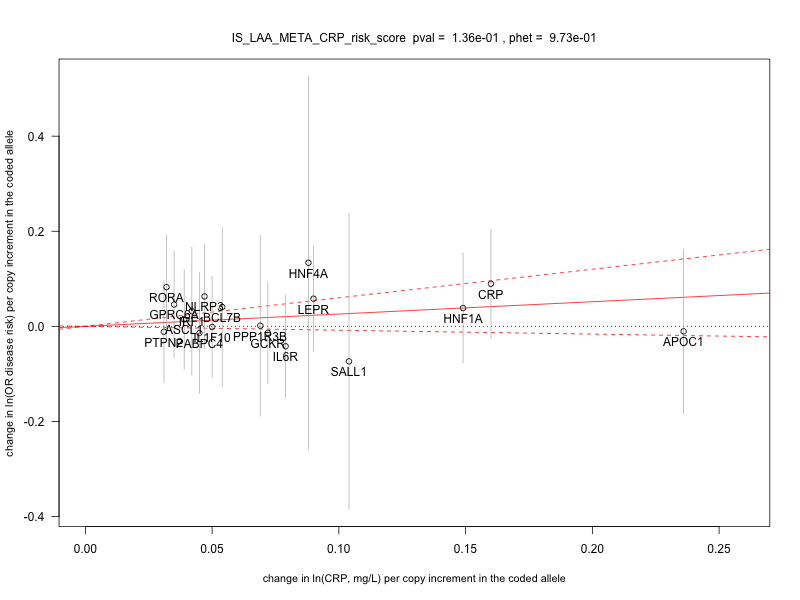


## S. Ischemic Stroke (Small Vessel Disease)


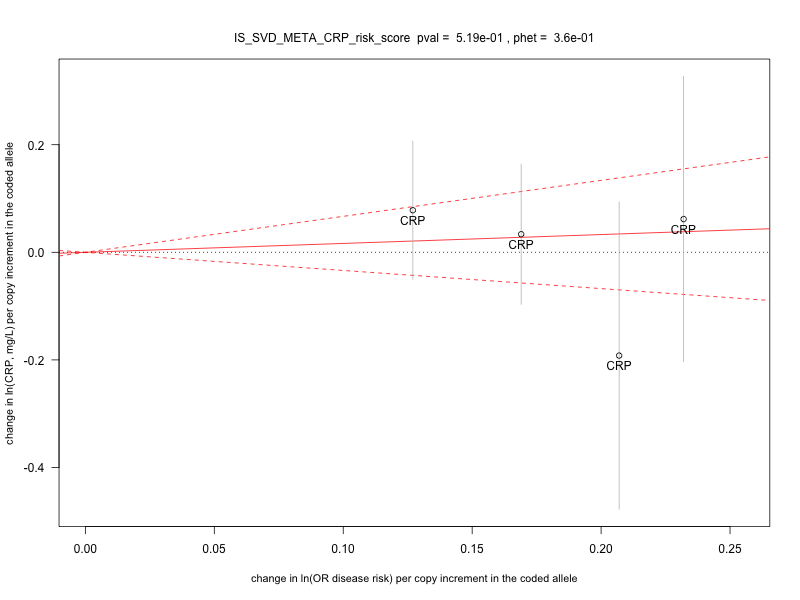

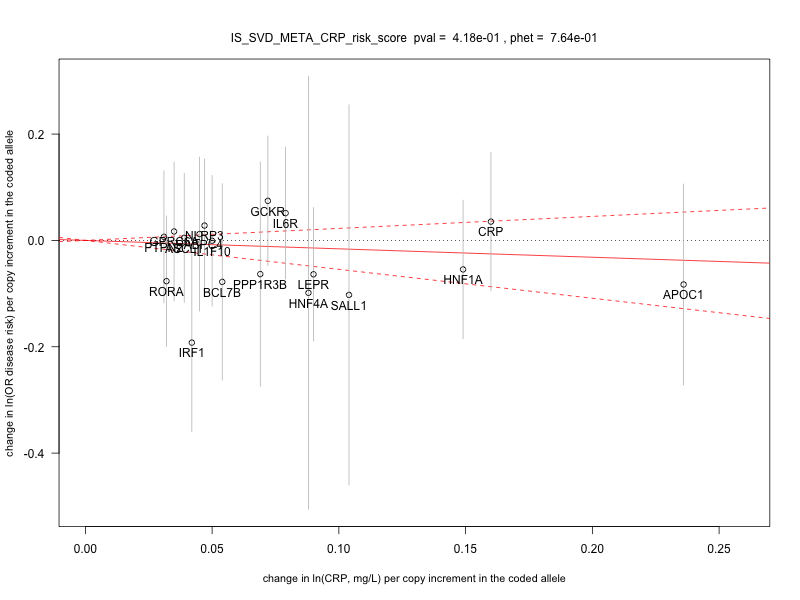


##
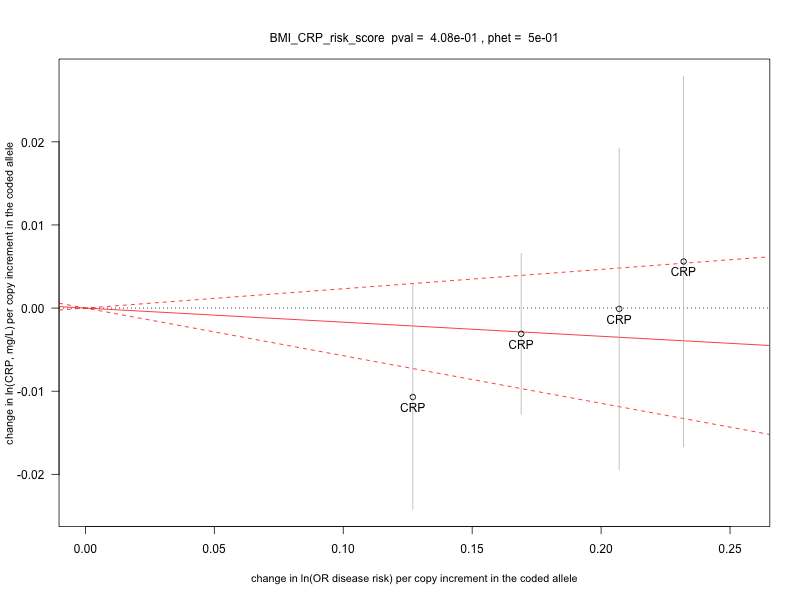

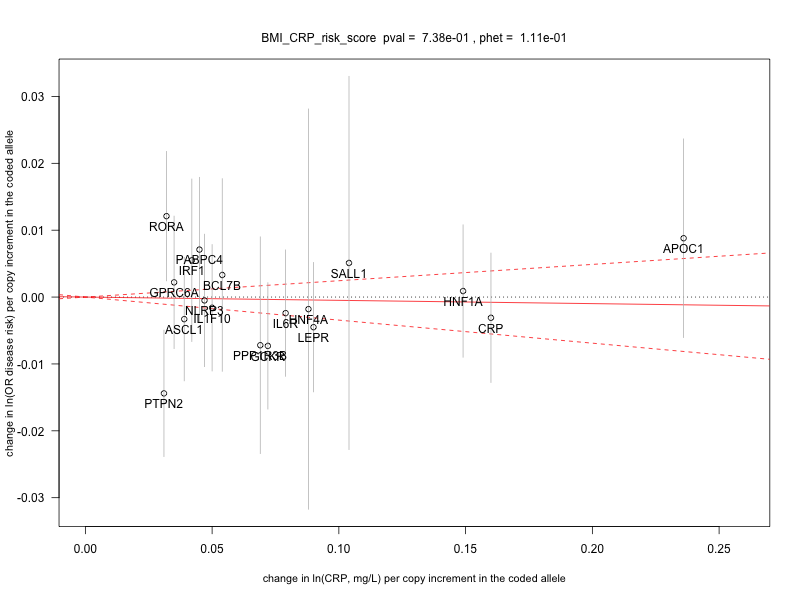
T. Body Mass Index

## U. Type II Diabetes

**
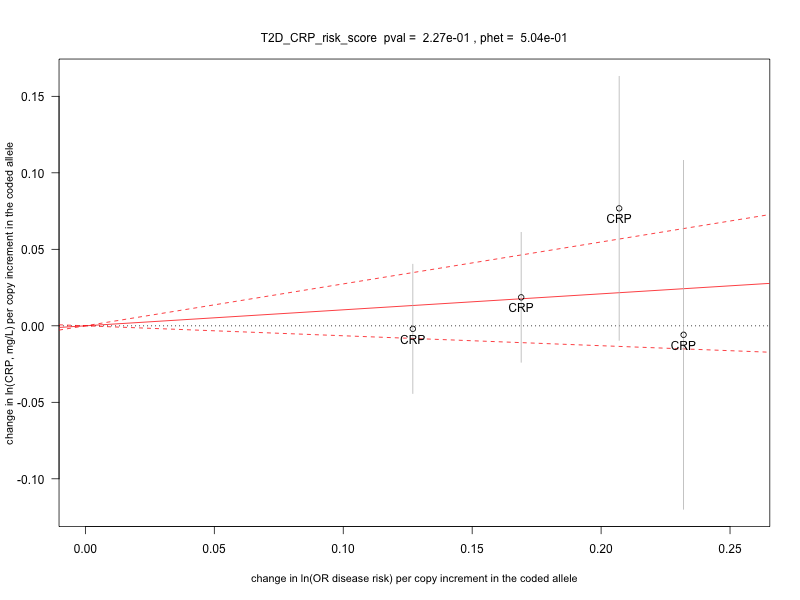
**
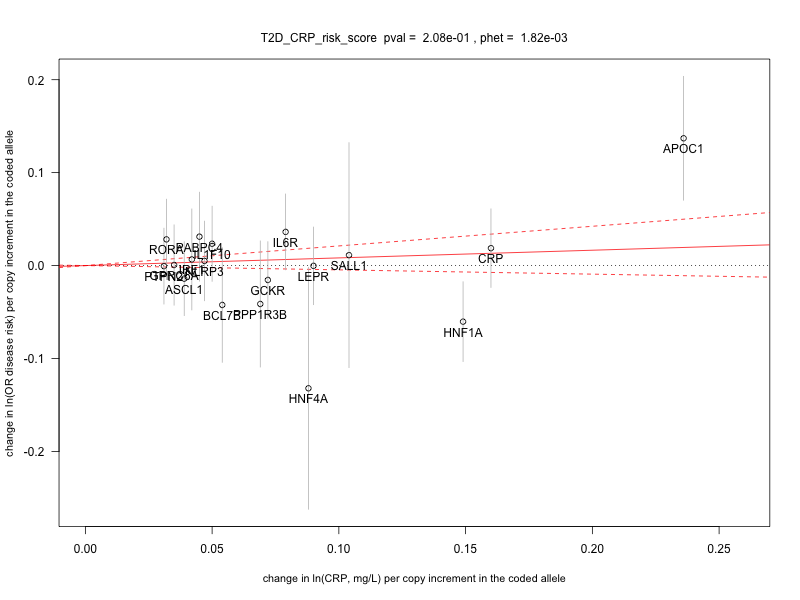


## V. Chronic Kidney Disease


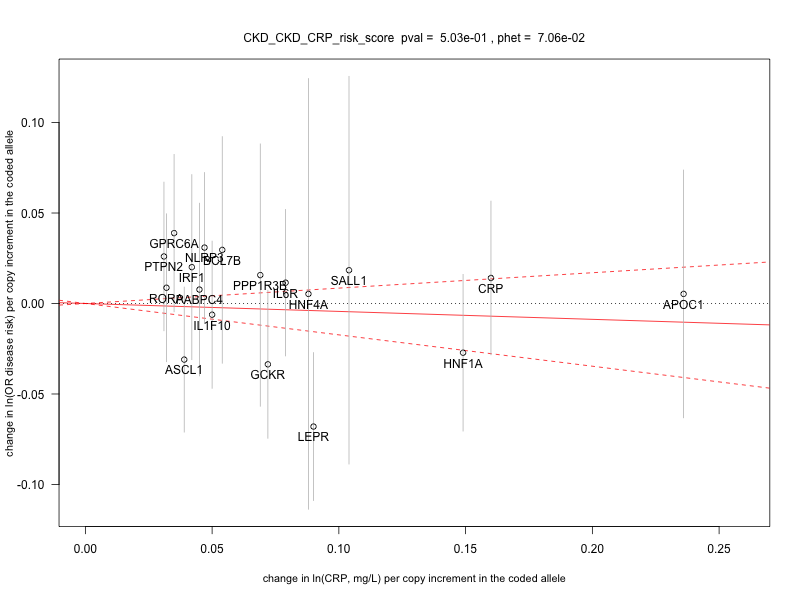

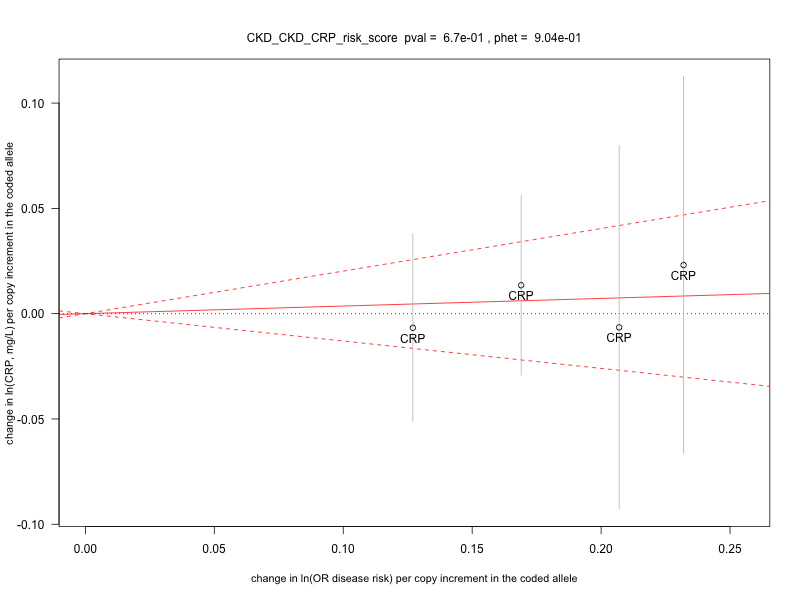


##
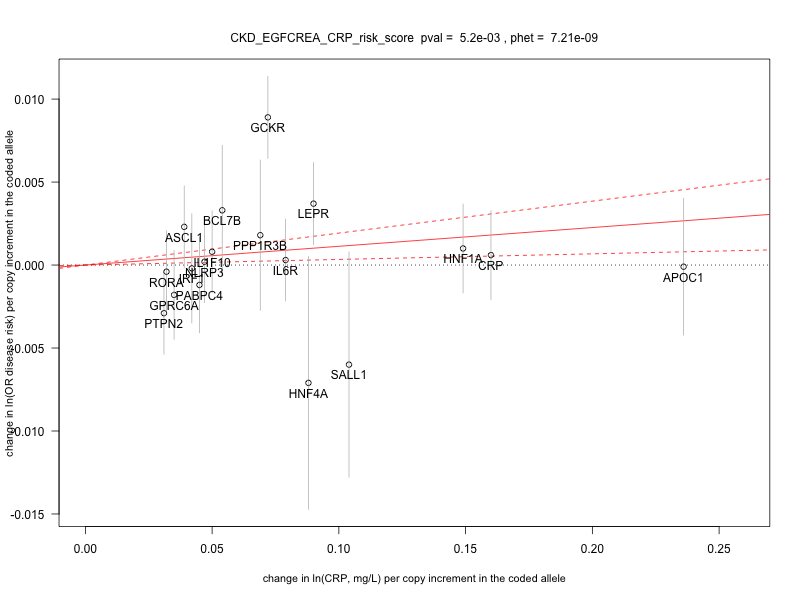

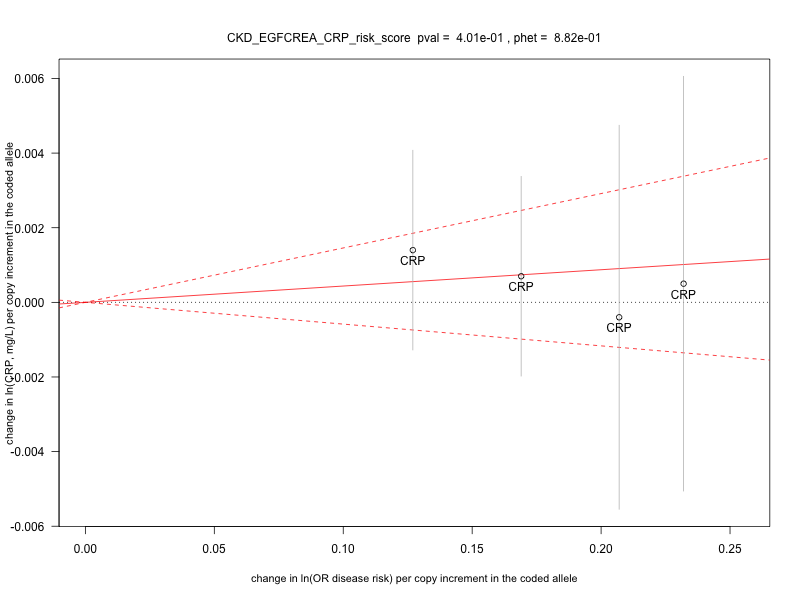
W. eGFR for Creatinine

## X. Serum Albumin Levels


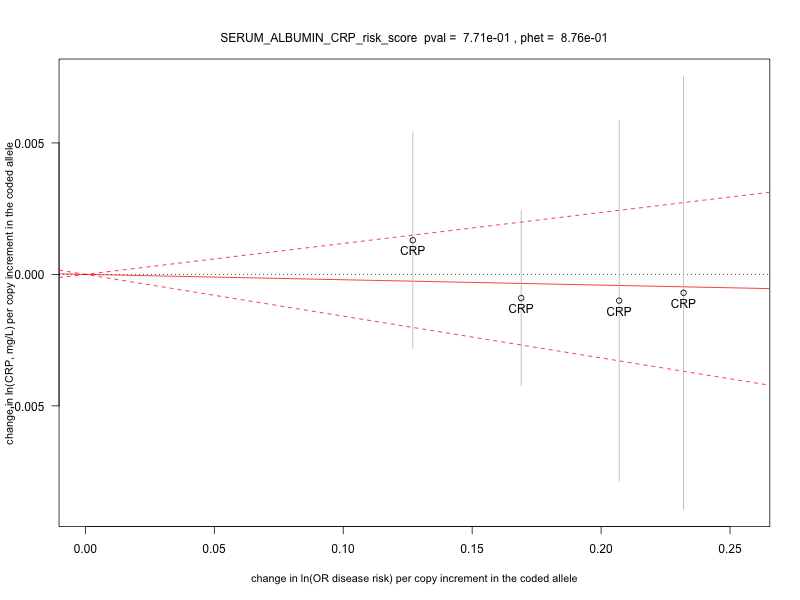

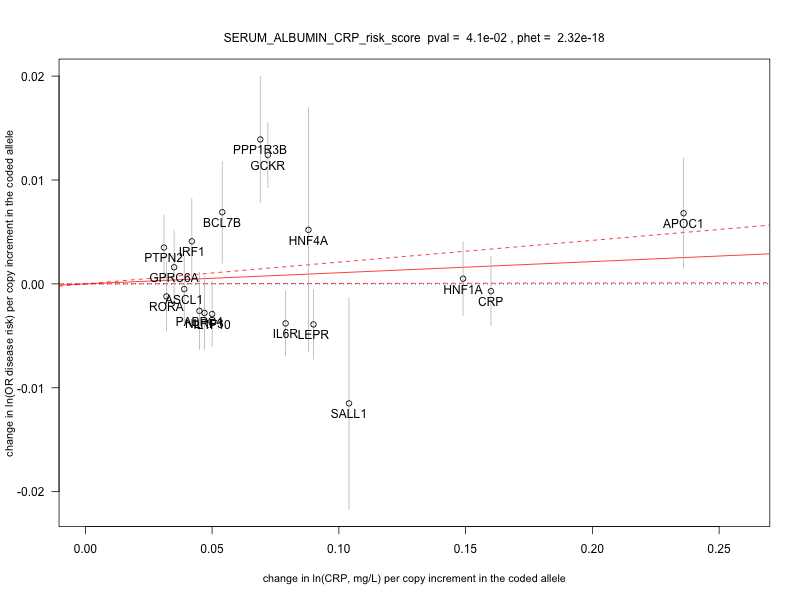


## Y. Serum Protein Levels


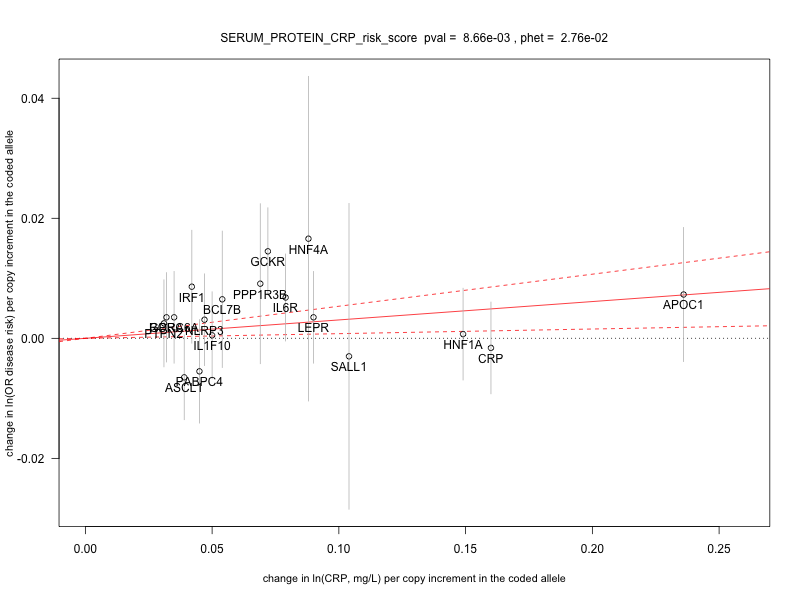

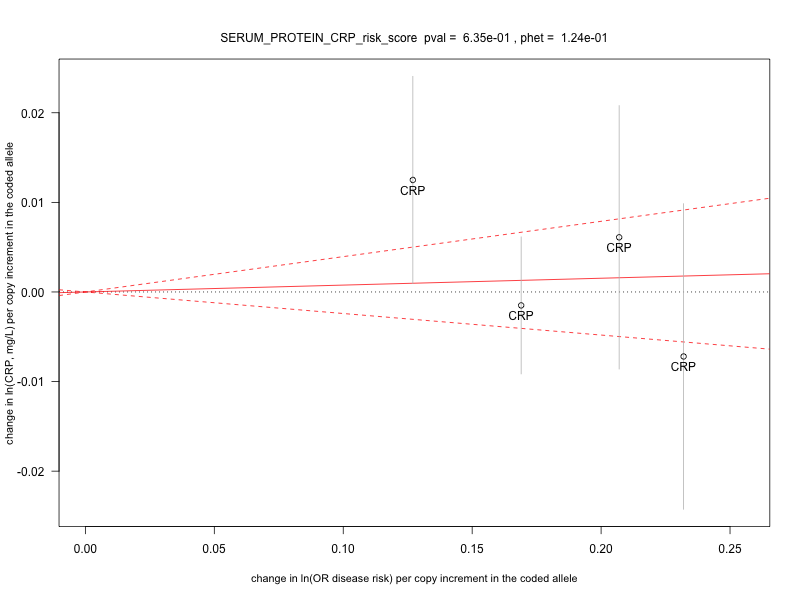


##
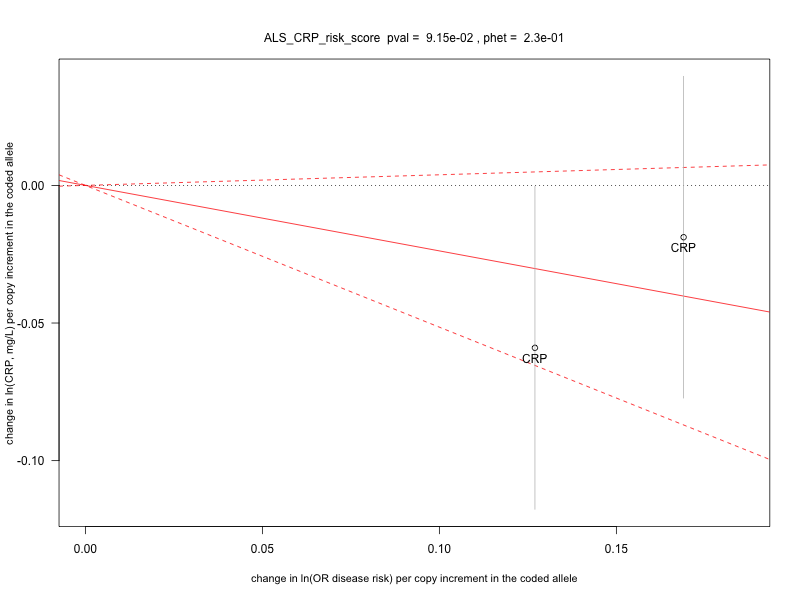

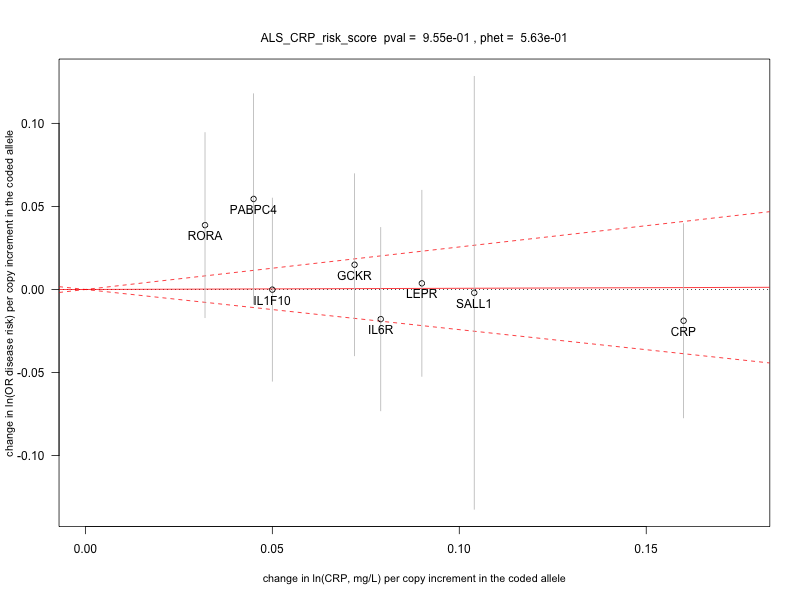
Z. Amyotrophic Lateral Sclerosis

##

## AA. Alzheimer's Disease


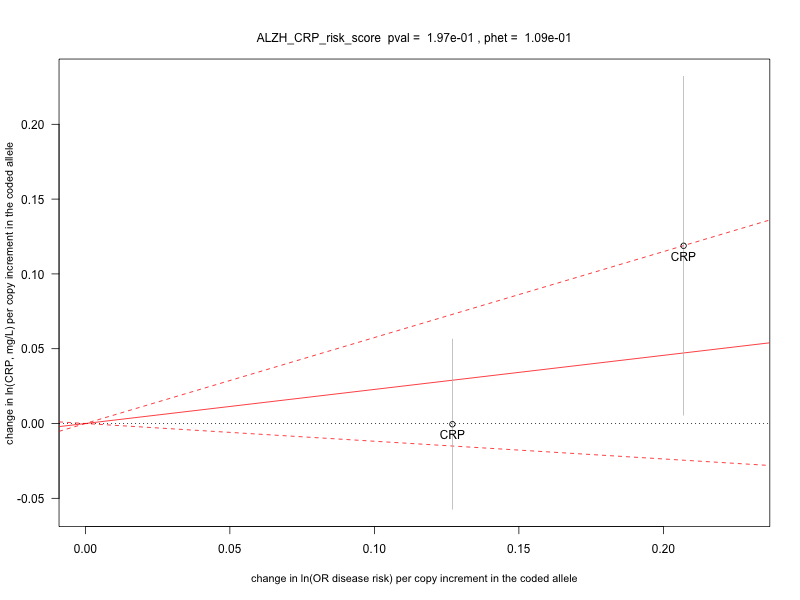

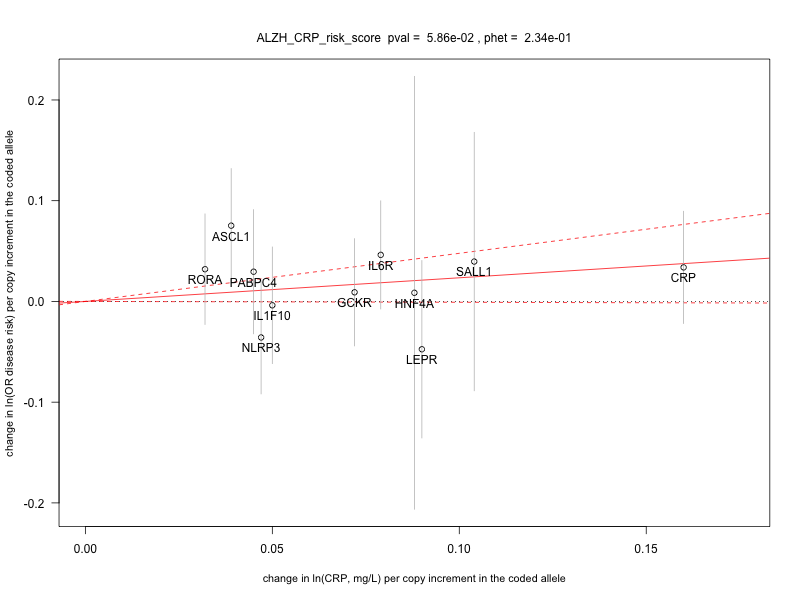


## AB. Parkinsons's Disease


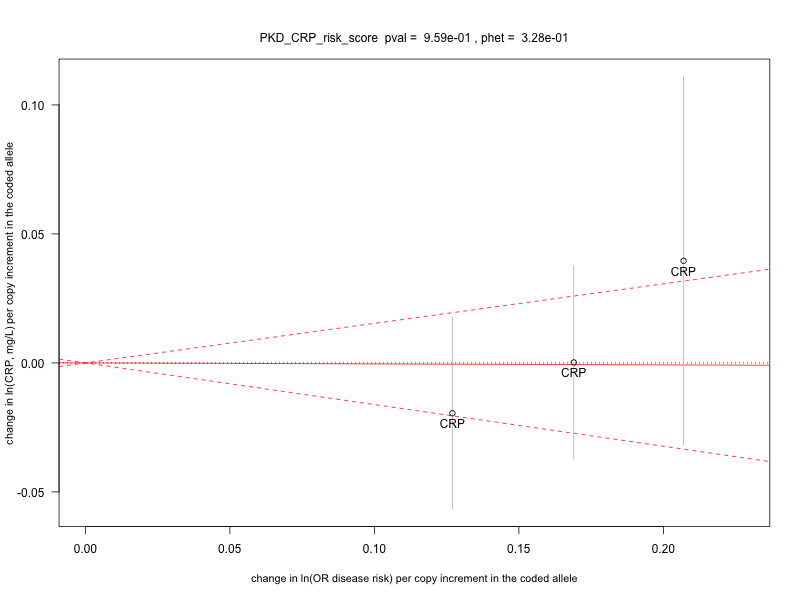

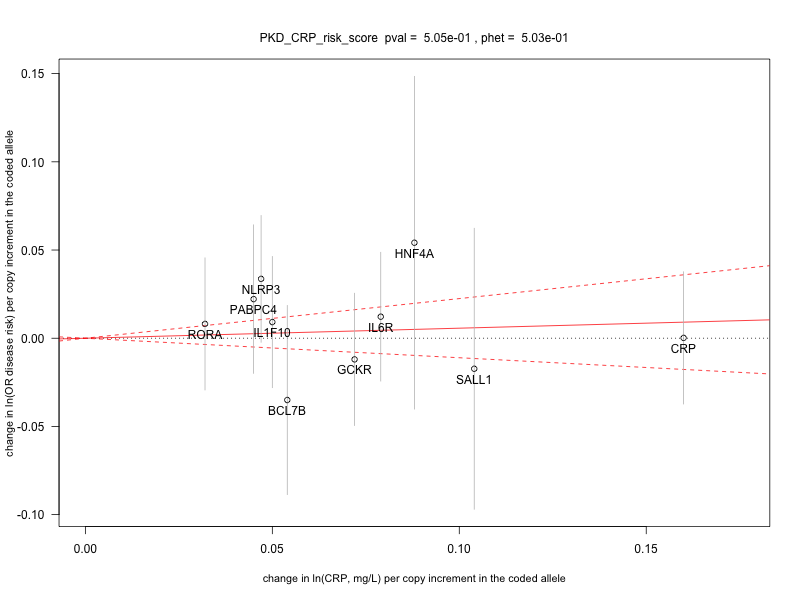


##
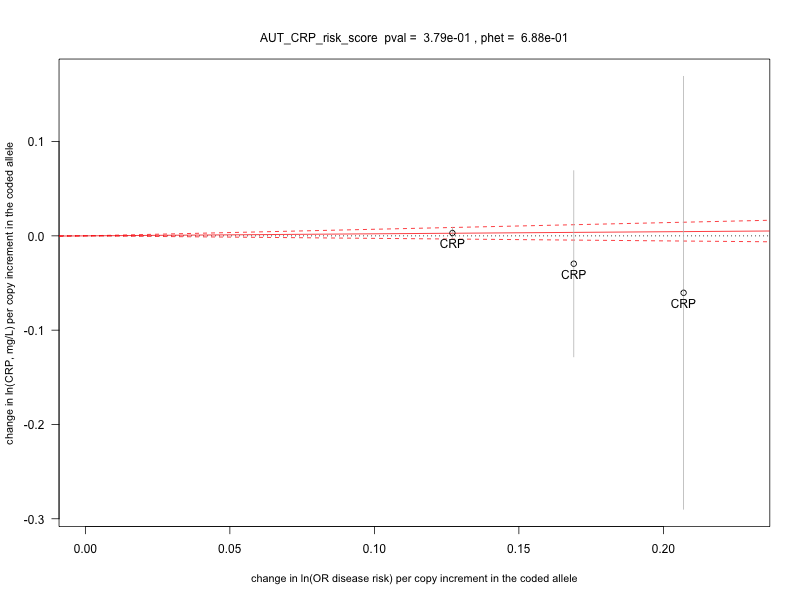

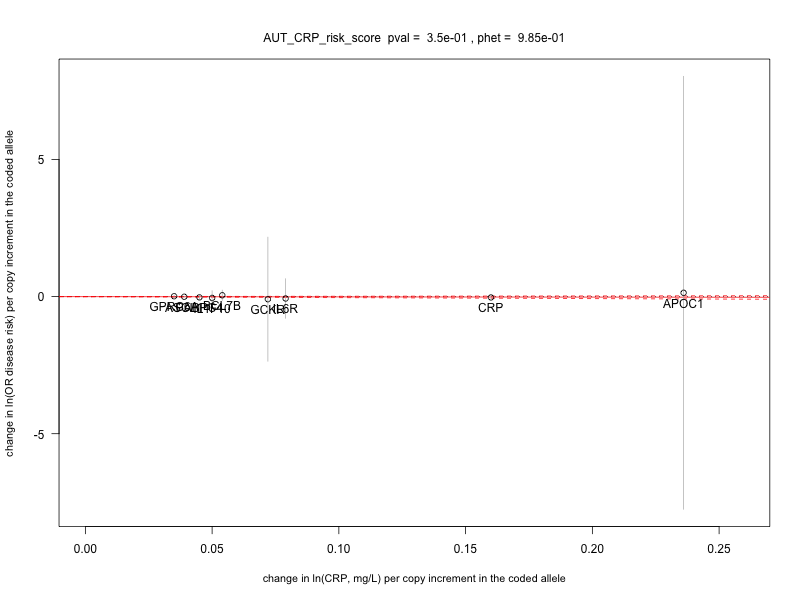
AC. Autism

##

## AD. Bipolar Disorder


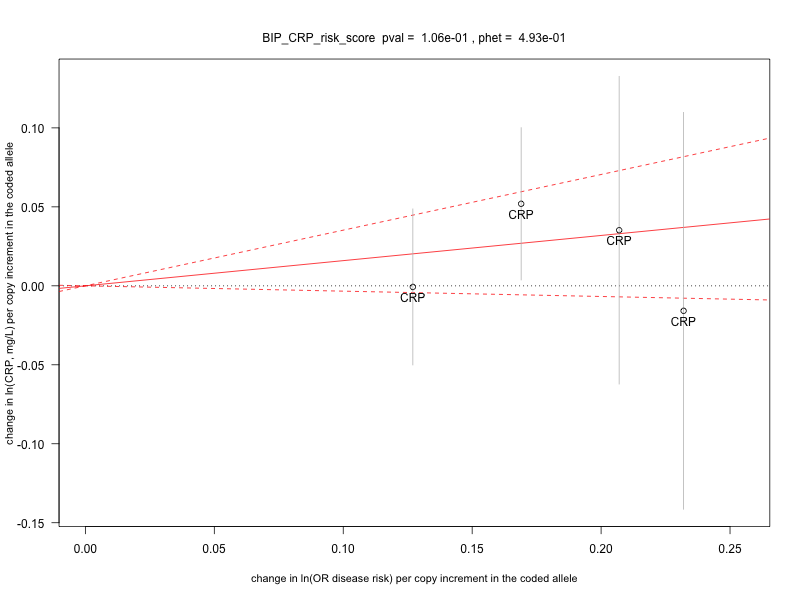

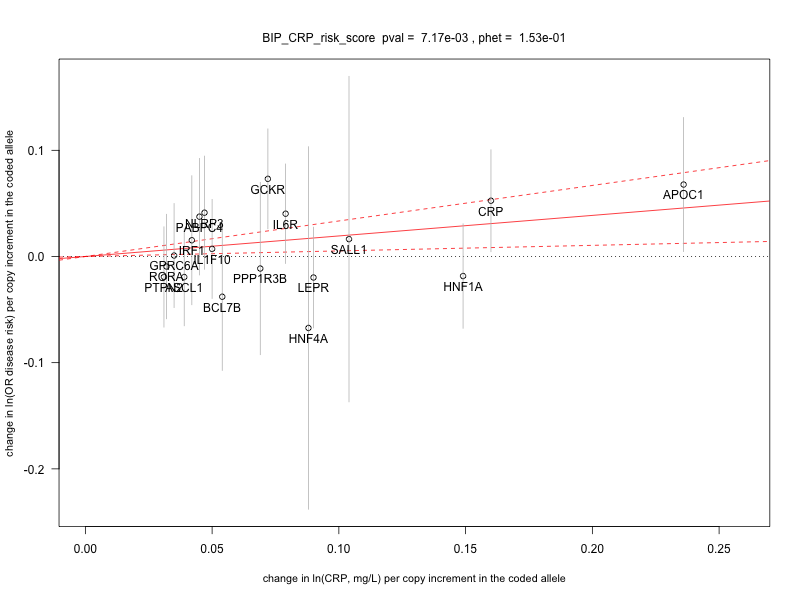


## AE. Major Depressive Disorder


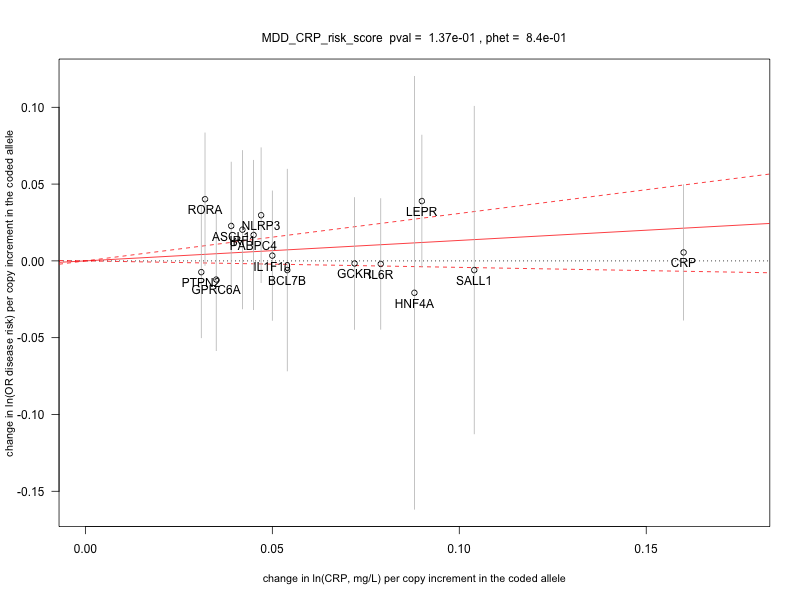

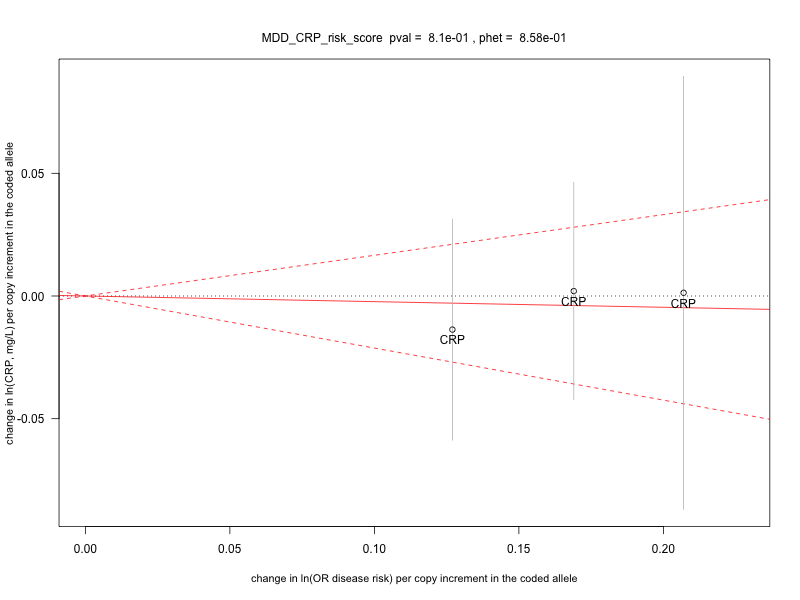


##
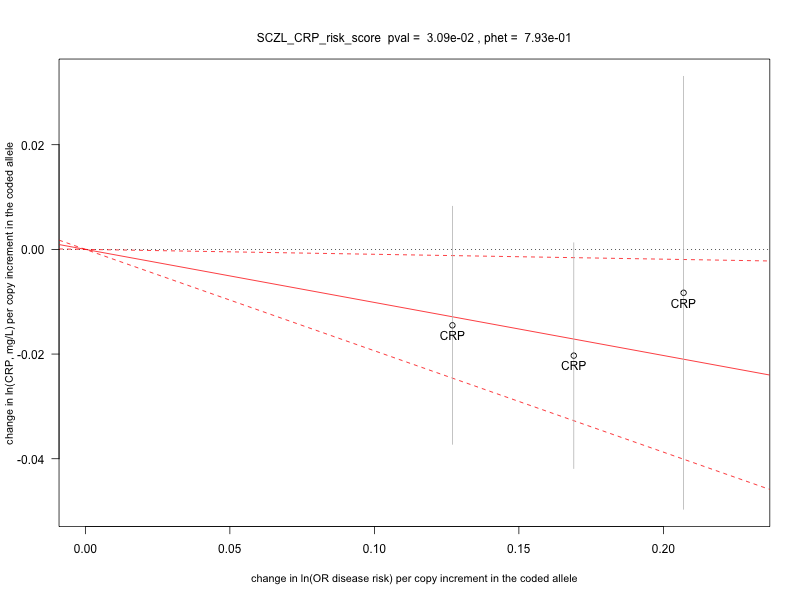

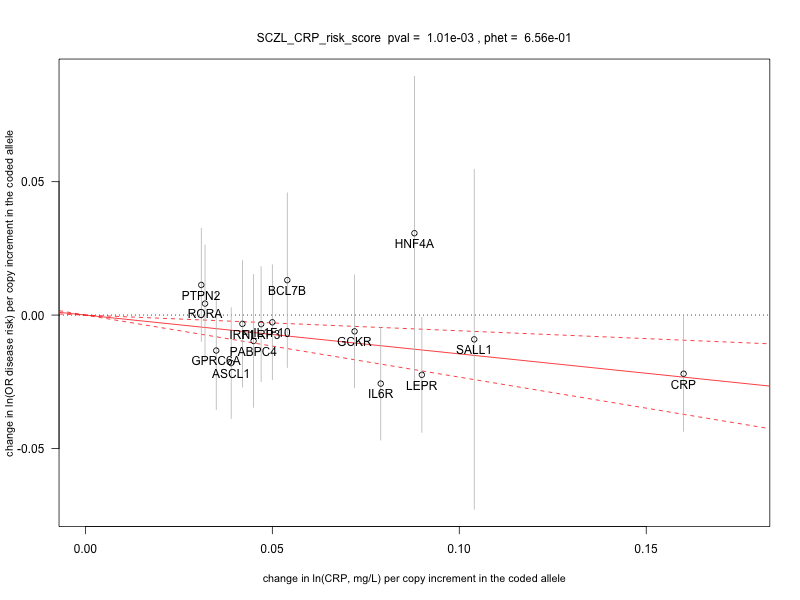
AF. Schizophrenia
