# Supplementary material for: Investigating the Causal Relationship of C-Reactive Protein with 32 Complex Somatic and Psychiatric Outcomes: A Large-Scale Cross-Consortium Mendelian Randomization Study
Source: PLoS Med. 2016 Jun 21;13(6):e1001976. doi: 10.1371/journal.pmed.1001976 (PMC4915710; doi:10.1371/journal.pmed.1001976)
Supplement: S1 Financial Disclosure — (PDF) [file pmed.1001976.s004.pdf]

None of the study sponsors had a role in the study design, data collection, analysis and interpretation, report writing, or the decision to submit the report for publication.

BPP was supported by a scholarship from Graduate School of Medical Sciences, UMCG, Groningen, The Netherlands, and by grants from the Economic & Social Research Council (ES/H029745/1) and the Wellcome Trust (WT098051), the United Kingdom. AV was supported by a scholarship from Isfahan University of Medical Sciences, Isfahan, Iran and a scholarship from Graduate School of Medical Sciences, UMCG. Groningen, The Netherlands. AA is supported by a Rubicon grant from the Netherlands Organization for Scientific Research (NWO project no. 825.13.004) and by Medical Research Council UK (grant reference no. MC-U106179471). H.M. is supported by an NIHR Senior Investigator award. His work is supported by a NIHR Comprehensive Biomedical Research Centre award to Cambridge University Hospitals. R.K.W. is supported by a VIDI grant (016.136.308) from the Netherlands Organization for Scientific Research (NWO). AM is a Wellcome Trust Senior Fellow in Basic Biomedical Science (grant number WT098017). PM is supported by the research program of the NIHR Barts Cardiovascular Biomedical Research Unit.

PAGE was supported by NIH Grants R01AR042742, R01AR054966, and R01AR050511. SSc consortia was supported by the grant SAF2012-34435 from the Spanish Ministry of Economy and Competitiveness and CTS-4977 from Junta de Andalucía, and is sponsored by the Orphan Disease Program grant from the European League Against Rheumatism (EULAR). The US analyses were supported by the NIH/NIAMS R01 AR055258, Two-Stage Genome Wide Association Study in Systemic Sclerosis.

T1DGC would like to state that all genotyping and other aspects of the study were funded by an Institutional Development Fund paid to the Center for Applied Genomics from the Children's Hospital of Philadelphia." are funded in part by the National Institute of Diabetes and Digestive and Kidney Diseases (NIDDK) award (DP3 DK085708) and SFAG and HH by a Development award from the Cotswold Foundation. T1D was also funded by the Children's Hospital of Philadelphia, Genome Canada through the Ontario Genomics Institute, and the Juvenile Diabetes Research Foundation. This research utilizes resources provided by the Type 1 Diabetes Genetics Consortium, a collaborative clinical study sponsored by the National Institute of Diabetes and Digestive and Kidney Diseases (NIDDK), National Institute of Allergy and Infectious Diseases (NIAID), National Human Genome Research Institute (NHGRI), National Institute of Child Health and Human Development (NICHD), and Juvenile Diabetes Research Foundation International (JDRF) and supported by U01 DK062418., and the Wellcome Trust under award 076113.

This manuscript was not prepared in collaboration with investigators of the T1DGC study, except for those listed as authors on the current manuscript, and does not necessarily reflect the opinions or views of the T1DGC study, the NIDDK Central Repositories, or the NIDDK. The Diabetes Control and Complications Trial (DCCT) and its follow-up the

Epidemiology of Diabetes Interventions and Complications (EDIC) study were conducted by the DCCT/EDIC Research Group and supported by National Institute of Health grants and contracts and by the General Clinical Research Center Program, NCRR. This manuscript was not prepared under the auspices of the DCCT/EDIC study and does not represent analyses or conclusions of the DCCT/EDIC study group. The Genetics of Kidneys in Diabetes (GoKinD) Study was conducted by the GoKinD Investigators and supported by the Juvenile Diabetes Research Foundation, the CDC, and the Special Statutory Funding Program for Type 1 Diabetes Research administered by the National Institute of Diabetes and Digestive and Kidney Diseases (NIDDK). This manuscript was not prepared in collaboration with investigators of the GoKinD study and does not necessarily reflect the opinions or views of the GoKinD study, the NIDDK Central Repositories, or the NIDDK.

GD: Cardiff University was supported by the Wellcome Trust, Medical Research Council (MRC), Alzheimer's Research UK (ARUK) and the Welsh Assembly Government. Cambridge University and Kings College London acknowledge support from the MRC. ARUK supported sample collections at the South West Dementia Bank and the Universities of Nottingham, Manchester and Belfast. The Belfast group acknowledges support from the Alzheimer's Society, Ulster Garden Villages, N. Ireland R&D Office and the Royal College of Physicians/Dunhill Medical Trust. The MRC and Mercer's Institute for Research on Ageing supported the Trinity College group. The South West Dementia Brain Bank acknowledges support from Bristol Research into Alzheimer's and Care of the Elderly. The Charles Wolfson Charitable Trust supported the OPTIMA group. Washington University was funded by NIH grants, Barnes Jewish Foundation and the Charles and Joanne Knight Alzheimer's Research Initiative. Patient recruitment for the MRC Prion Unit/UCL Department of Neurodegenerative Disease collection was supported by the UCLH/UCL Biomedical Centre and NIHR Queen Square Dementia Biomedical Research Unit. LASER-AD was funded by Lundbeck SA. The Bonn group was supported by the German Federal Ministry of Education and Research (BMBF), Competence Network Dementia and Competence Network Degenerative Dementia, and by the Alfried Krupp von Bohlen und Halbach-Stiftung. GD also used samples ascertained by the NIMH AD Genetics Initiative. The Framingham Heart Study (FHS) and inflammation biomarkers collection is supported by the National Institute of Health, USA (grants numbers: R01 HL076784; R01 AG028321; and RO1 HL64753, contract numbers HHSN268201500001I & N01-HC 25195).

The ALS consortium is an EU Joint Programme - Neurodegenerative Disease Research (JPND) project. The project is supported through the following funding organisations under the aegis of JPND - www.jpnd.eu (United Kingdom, Medical Research Council and Economic and Social Research Council). AAC receives salary support from the National Institute for Health Research (NIHR) Dementia Biomedical Research Unit at South London and Maudsley NHS Foundation Trust and King's College London. The views expressed are those of the authors and not necessarily those of the NHS, the NIHR or the Department of Health.

The work leading up to this publication was funded by the European Community's Health Seventh Framework Programme (FP7/2007–2013; grant agreement number 259867).
