# Supplementary material for: Investigating the Causal Relationship of C-Reactive Protein with 32 Complex Somatic and Psychiatric Outcomes: A Large-Scale Cross-Consortium Mendelian Randomization Study
Source: PLoS Med. 2016 Jun 21;13(6):e1001976. doi: 10.1371/journal.pmed.1001976 (PMC4915710; doi:10.1371/journal.pmed.1001976)

Investigating the causal relationship of C-reactive protein with 32 complex somatic and psychiatric outcomes: A large scale cross-consortia Mendelian randomization study.

Supplementary Methods 1:  Linkage disequilibrium of the four GRS*_CRP_* SNPs.

**Methods:** The figure was generated using Haploview. LD values (r^2^) were calculated using Hapmap Phase II+III (r28, CEU population) data. r^2^ values are shown between SNPs pairs on a scale between 0 (no LD) to 100 (full LD) in diamonds colored on a corresponding grayscale, where no LD is represented by white and full LD by black. This plot indicates low LD between each of the four SNPs spanning the CRP gene.

## S2 Fig: Linkage disequilibrium (LD) heatplot for 4 SNPs as used in the GRS_CRP_ IV.


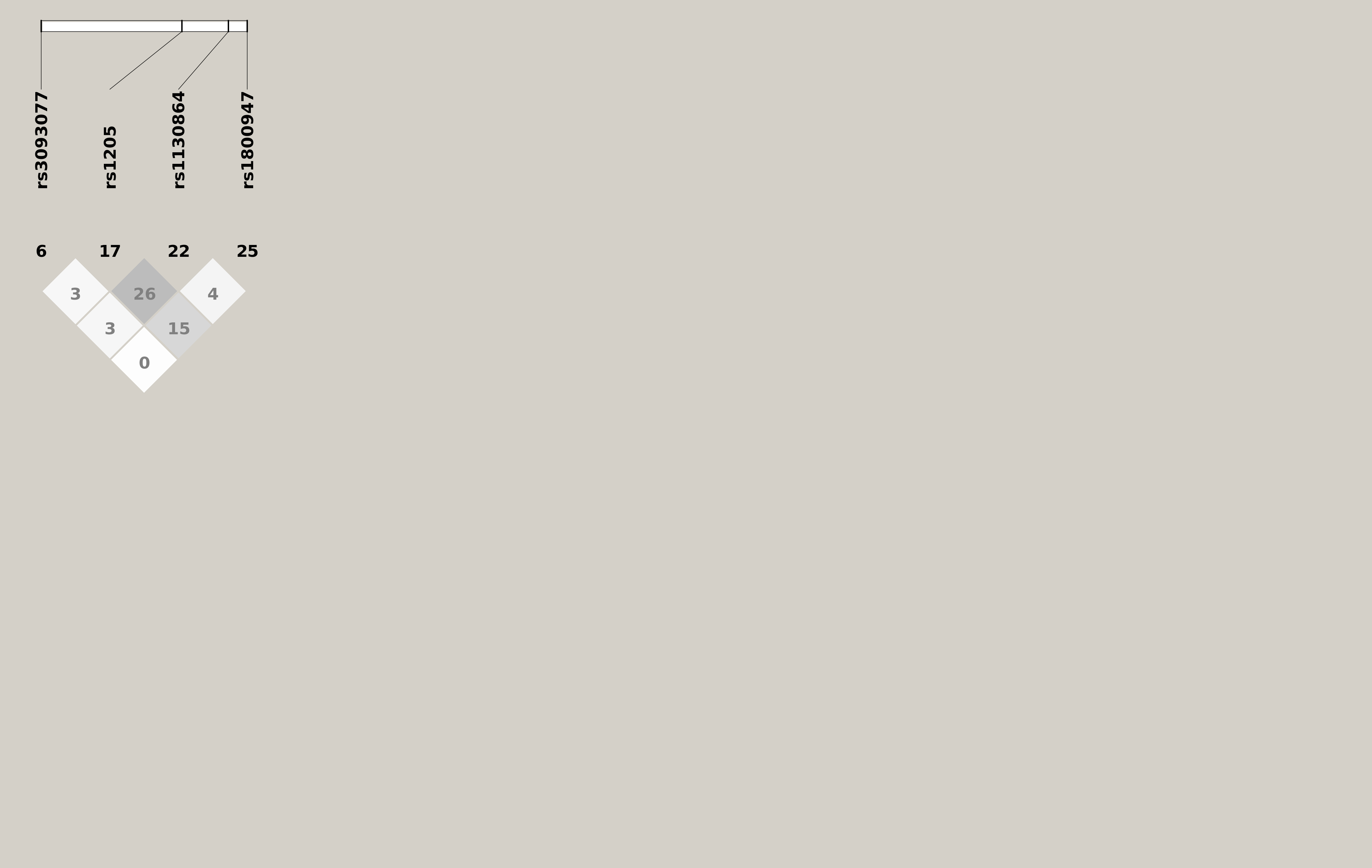

Supplement: S1 Methods — (DOCX) [file pmed.1001976.s005.docx]
