# Supplementary material for: Investigating the Causal Relationship of C-Reactive Protein with 32 Complex Somatic and Psychiatric Outcomes: A Large-Scale Cross-Consortium Mendelian Randomization Study
Source: PLoS Med. 2016 Jun 21;13(6):e1001976. doi: 10.1371/journal.pmed.1001976 (PMC4915710; doi:10.1371/journal.pmed.1001976)
Supplement: S2 Methods — (DOCX) [file pmed.1001976.s006.docx]

Investigating the causal relationship of C-reactive protein with 32 complex somatic and psychiatric outcomes: A large scale cross-consortia Mendelian randomization study.

Supplementary Methods - 2: CRP GRSG*_WAS_* in AD and BMI.

## A. Calculation of the Alzheimer’s Disease GWAS summary statistics.

We received summary statistics (SNP, OR, SE, P, Reference Allele, Other Allele, OR 95 L ,OR 95 U) from the Genetic and Environmental Risk in Alzheimer's Disease (GERAD) consortium for three separate Alzheimer’s datasets ; from the TGEN consortium, from the ADNI consortium, and from the GERAD consortium for up to 4,663 cases and 8,357 controls. We next performed an inverse variance weighted fixed effects analysis using GWAMA^1^ to calculate combined effect sizes and standard errors, which were subsequently used in our genetic risk scores.

## B. Calculation of the BMI GWAS summary statistics.

We downloaded sex-stratified summary statistics for BMI from Randall et.al ^2^. From <https://www.broadinstitute.org/collaboration/giant/index.php/GIANT_consortium_data_files>. We next performed an inverse variance weighted fixed effects analysis using GWAMA^1^ to calculate combined effect sizes and standard errors, which were subsequently used in our genetic risk scores.

^1^ Mägi R, Morris AP. GWAMA: software for genome-wide association meta-analysis. BMC Bioinformatics. 2010 May 28;11:288.

^2^ Randall JC. Sex-stratified genome-wide association studies including 270,000 individuals show sexual dimorphism in genetic loci for anthropometric traits. PLoS Genet. 2013 Jun;9(6):e1003500.
