# Supplementary material for: Investigating the Causal Relationship of C-Reactive Protein with 32 Complex Somatic and Psychiatric Outcomes: A Large-Scale Cross-Consortium Mendelian Randomization Study
Source: PLoS Med. 2016 Jun 21;13(6):e1001976. doi: 10.1371/journal.pmed.1001976 (PMC4915710; doi:10.1371/journal.pmed.1001976)
Supplement: S3 Methods — (DOCX) [file pmed.1001976.s007.docx]

**Investigating the causal relationship of C-reactive protein with 32 complex somatic and psychiatric outcomes: A large scale cross-consortia Mendelian randomization study.**

**Supplementary Methods - 3: WEBLINKS.**

*Software :*

Genetics ToolboX (version 0.0.8):

<http://cran.r-project.org/web/packages/gtx/index.html>

Functions for Medical Statistics Book with some Demographic Data

<https://cran.r-project.org/web/packages/fmsb/>

<http://www.genemania.org>

*Publicly downloaded GWAS summary statistics:*

*1. GIANT BMI summary statistics:*

[*www.broadinstitute.org/collaboration/giant/index.php/GIANT_consortium_data_files*](http://www.broadinstitute.org/collaboration/giant/index.php/GIANT_consortium_data_files)

*2. CARDIoGRAM CAD summary statistics:*

[*www.cardiogramplusc4d.org*](http://www.cardiogramplusc4d.org)

*3. Rheumatoid arthritis summary statistics:*

[*www.broadinstitute.org/ftp/pub/rheumatoid arthritis/Stahl_etal_2010NG/*](http://www.broadinstitute.org/ftp/pub/rheumatoid%20arthritis/Stahl_etal_2010NG/)

*4. PGC consortium (psychiatric) summary statistics:*

[*www.med.unc.edu/pgc/downloads*](http://www.med.unc.edu/pgc/downloads)

*5. Systemic Lupus Erythematosus*

*Data was downloaded through dbGaP :* [*http://www.ncbi.nlm.nih.gov/gap*](http://www.ncbi.nlm.nih.gov/gap)

*Study name : Whole Genome Association Study of Systemic Lupus Erythematosus*

*dbGaP Study Accession: phs000122.v1.p1*

*Analysis Name and Accession*

*Name: Whole Genome Association Study of Systemic Lupus Erythematosus*

*Accession: pha002848.1*
