# Supplementary material for: Investigating the Causal Relationship of C-Reactive Protein with 32 Complex Somatic and Psychiatric Outcomes: A Large-Scale Cross-Consortium Mendelian Randomization Study
Source: PLoS Med. 2016 Jun 21;13(6):e1001976. doi: 10.1371/journal.pmed.1001976 (PMC4915710; doi:10.1371/journal.pmed.1001976)
Supplement: S4 Methods — (DOCX) [file pmed.1001976.s008.docx]

Investigating the causal relationship of C-reactive protein with 32 complex somatic and psychiatric outcomes: A large scale cross-consortia Mendelian randomization study.

Supplementary Methods - 4: CRP Polygenic risk score (CRP*_PRS_*) in Schizophrenia.

**Background:** We observed a causally protective effect of CRP GWSAs against Schizophrenia. We aim to determine whether a) CRP-associated risk alleles are associated with schizophrenia by employing the use of polygenic risk scores, and b) to determine if the relationship between CRP and schizophrenia is due to genetic pleiotropy or clinical heterogeneity. Here below we explain study design and applied methods.

**Sample Description:** Individual-level dosage data was retrieved from the Psychiatric Genomics Consortium (PGC) Schizophrenia dataset, consisting of 36 independent cohorts with a combined 25629 cases and 30976 controls. 3 family-based samples of European ancestry (1,235 parent affected-offspring trios) were excluded from our analysis. For a more detailed treatment of the PGC schizophrenia dataset, refer to the methods section of the full paper (Ripke et al., 2014).

**Selection of CRP-associated SNPs :** Summary statistics (including SNP RSID, chromosome and position, coded allele, beta, standard error, and *p* value) were retrieved from a meta-analysis of 15 independent European population-based studies with 66,185 participants (Dehghan et al., 2011). After accounting for the replication sample, 18 SNPs reached genome-wide significance (*p* < 5 x 10^8^) and were thus included in the study. All non-genome-wide significant SNPs were clumped based on linkage disequilibrium estimates using plink v2 with the following flags: --clump-p1 1 –clump-p2 1 –clump-r2 0.1 –clump-kb 1000. Thus, all SNPs included in the analyses were LD-independent, as defined by SNPs with r^2^ < 0.1 (Purcell et al., 2007). We subsequently grouped the sub-threshold CRP-associated SNPs at the following *p*-value thresholds: 1x10^-4^, 0.001, 0.01, 0.05, and 0.1.

**Statistical Analysis:** Polygenic risk scores were calculated for each individual by summing the total effect of the SNP dosages by its effect size. This was performed for each independent schizophrenia cohort using plink v2 with the –score and –qscore flags (Purcell et al., 2007). A fixed effects, inverse variance weighted meta-analysis was performed across all 36 cohorts using a custom R script. Briefly, beta estimates (log odds ratios) was weighted by the estimated standard errors. 10 PCs were regressed out of each independent cohort to account for population stratification.

**Results**

**Examining the association between the 18 CRP-associated risk alleles in GWAS and schizophrenia**

13 out of the 18 SNPs associated with elevated CRP levels were protective against schizophrenia (Fig 1, and S3 Fig). Five out of the 13 SNPs reached a significance threshold of *p* < 0.05 (S3 Fig). A sign test was performed to determine if the risk alleles for CRP are shared with schizophrenia more than expected (P*<*0.096).

##

## S3 Fig: 18 risk alleles associated with elevated CRP levels that reached genome-wide significance, and their corresponding schizophrenia odds ratios.
