# Supplementary material for: Investigating the Causal Relationship of C-Reactive Protein with 32 Complex Somatic and Psychiatric Outcomes: A Large-Scale Cross-Consortium Mendelian Randomization Study
Source: PLoS Med. 2016 Jun 21;13(6):e1001976. doi: 10.1371/journal.pmed.1001976 (PMC4915710; doi:10.1371/journal.pmed.1001976)
Supplement: S5 Methods — (DOCX) [file pmed.1001976.s009.docx]

Investigating the causal relationship of C-reactive protein with 32 complex somatic and psychiatric outcomes: A large scale cross-consortia Mendelian randomization study.

Supplementary Methods - 5: In-silico (gene) pathway analyses highlight the role of IFN in the causal pathway between CRP and SCZ.

**Background:** We aimed to explore the possible underlying pathways that may underwrite the protective causal association between CRP and schizophrenia. We answered this question by performing two studies: In the first study we aimed to elucidate through which potential pathways CRP associated proteins (encoded by those genes which are tagged by CRP associated gSNPs and eSNPs potentially are linked to genes associated to SCZ, more specifically, those that are differentially expressed in schizophrenia cases versus controls

*Study I : Identification of common pathways between genes / their encoded proteins involved in determination on CRP levels and proteins differentially expressed in schizophrenia cases versus controls.*

The following 3 steps were taken:

1.1) We firstly retrieved a list of CRP-associated genes whom harboured by loci associated with CRP in the large GWAS meta-analysis for CRP to date [6] and genes for which their expression was associated with one or more of the above mentioned 18 loci as presented by Vaez et.al.[7]. These groups of genes and their selection processes have been discussed in greater detail in our previous publication by Vaez et.al.[7] and presented in Table 2 of the respective paper and for convenience listed again in our S4 Table (tabname “CRP genes”). We used the 40 CRP genes from Table 2 in Vaez et. al. [7] and performed enrichment analyses as explained in the same publication [7] using the well-established pathway and network analysis suite Genemania (S3 Methods Weblinks) [8], the result of which is presented in S Table 6 (tabname “S6. CRP enriched pathways”). As previously observed[7] the most enriched functions within this geneset are related to the type I interferon signaling pathway (FDR=6.08x10^-9^).

1.2) Secondly, we retrieved a list of 144 proteins, which are significantly differentially expressed in brain tissues of schizophrenia cases compared with matched controls, hence are expected to be involved in the mechanism of schizophrenia. These proteins are presented in the study by Hwang et.al. [9] in their respective S Table 4. These proteins are listed in our S6 Table (tabname “SCZ expr genes”). We performed functional enrichment analysis for this geneset in the same fashion as described above, the results of which are presented in S7 Table (tabname “SCZ expr enriched pathways”).

None of the differentially expressed proteins from the study of Hwang et.al overlap. with proteins encoded by genes identified by Vaez et.al. except the *HEYL*, which is a non-inflammatory gene. Surprisingly, we observed that the most significantly enriched function for the set of differentially expressed proteins is also the type I interferon signalling pathway (FDR=2.81x10^-12^) .

1.3) Lastly, to confirm the type I interferon signalling pathway may be the predominant pathway that is shared between these two sets of proteins, we performed functional prediction and pathway enrichment analyses by including all the 40 CRP associated and 144 SCZ associated genes as presented S8 Table (tabname “CRP & SCZ expr gene list”), the results of which are presented in S9 Table (tabname “CRP&SCZ enriched pathways”). We observed again that the type I interferon signalling pathway remained as the most significantly enriched pathway (FDR=8.60x10^-22^).

*Summary of study 1 : Identification of common pathways between genes / their encoded proteins involved in determination on CRP levels and proteins differentially expressed in schizophrenia cases versus controls.*

Our *in-silico* functional enrichment analysis from both CRP and SCZ associated genes showed the enrichment of pathways of “response to type I interferon”, “cellular response to type I interferon”, “type I interferon signaling pathway”.

We therefore speculate the protective causal effect of CRP might be explained by the fact that T-cell IFN cytokine release stimulates microglia to facilitate glutamate clearance in neuronal cells without evoking inflammatory mediators, and by contributing to restoration of normal homeostasis[1,2].

*Study 2 : Identification of pathways involved in schizophrenia based on loci identified in the largest Genome Wide Association Study for schizophrenia and their associated eQTLs in brain and blood, excluding any known CRP associated genes and associated eQTLs.*

Our second study intended to investigate through which pathways do **non-CRP** SCZ associated genes and their encoded proteins affect SCZ. We took a similar approach to our first study, where now instead we use the results from the largest meta-GWAS study for schizophrenia[10] as a basis as follows :

2.1) Firstly, we extracted the list of genes harboured by 108 SCZ associated loci at genome wide significance level from the largest meta-GWAs in SCZ published by the Schizophrenia Working Group of the Psychiatric Genomics Consortium, as listed in S Table 3 in the respective study[10] and in our S10 Table (tabname “SCZ 108 loci genes”).

2.2) Next we extracted all genes tagged by expression quantitative trait loci (eQTL) from a human brain cortex eQTL study and another eQTL study performed in peripheral blood cells as listed in S Table 4 in the same publication [10], and in our S11 Table (tabname “SCZ 108 loci eQTL”) and merged these with the list of genes harboured by 108 SCZ associated loci mentioned above. We removed duplicate genes and to focus on non-CRP associated pathways, we additionally removed genes overlapping with those from Vaez et. al., resulting in the removal of in total, C12orf42). The combined set of genes is presented in S12 Table (tabname “SCZ eQTL + 108 loci genes”).

2.3) We performed the same functional enrichment analyses as explained earlier on this combined set of 407 genes. The (non-)results are presented in S13 Table (tabname “eQTL&SCZ loci enr pathways”).

*Summary of Study 2 : Identification of pathways involved in schizophrenia based on loci identified in the largest Genome Wide Association Study for schizophrenia and their associated eQTLs in brain and blood, excluding any known CRP associated genes and associated eQTLs.*

Similar to the main manuscript, we failed to identify any significantly enriched pathways, in our case using Genemania, although nominally significantly enrichments were observed for several predefined candidate pathways such as calcium channels in the original publication. In our case we included also eQTL loci whereas the original study used the meta-analysis summary statistics. We did not identify any inflammatory related pathways these analyses. The report from the Schizophrenia Working Group of the Psychiatric Genomics Consortium does however provide some evidence for a role of the immune system in schizophrenia development when searching for the most relevant cell-types based on their genomic loci and cell and tissue type specific enhancers enrichment analyses ; two peaks can be observed in Fig 2 for B-lymphocyte lineages involved in acquired immunity (CD19 and CD20 cell lines), which remained significant even after excluding the extended MHC region and regions containing brain enhancers[10]. The main original study has not report any significantly enriched pathway, and neither did our analyses led to such a conclusion based on the presented data in [10] .

Taken together, our *in-silico* analyses point to a role for pathway associated to IFN response and metabolism that may possibly underlie protective effect of CRP in schizophrenia. Specifically, we speculate that CRP-IFN pathway perhaps contributes to neuroprotection by stimulating a phenotype in neuron supporting cells such as astrocytes or microglia that facilitates glutamate reduction as observed directly for IFN by Shaked et.al [2] and Garg et.al [1] leading to the protection of neurons against oxidative stress associated with an excess of glutamate.

**Associated files:**

Please find lists of trait-associated genes and results of pathway enrichment analyses in S4 Table to S13 Table. The first tab in this file, named as “Contents” contains a guide to the contents of this file.

**Web link:** <http://www.genemania.org>

**References:**

1. Garg SK, Banerjee R, Kipnis J. Neuroprotective immunity: T cell-derived glutamate endows astrocytes with a neuroprotective phenotype. J Immunol Baltim Md 1950. 2008;180: 3866–3873.
 2. Shaked I, Tchoresh D, Gersner R, Meiri G, Mordechai S, Xiao X, et al. Protective autoimmunity: interferon-gamma enables microglia to remove glutamate without evoking inflammatory mediators. J Neurochem. 2005;92: 997–1009. doi:10.1111/j.1471-4159.2004.02954.x
3. Javitt DC. Glutamatergic theories of schizophrenia. Isr J Psychiatry Relat Sci. 2010;47: 4–16.
 4. Marsman A, van den Heuvel MP, Klomp DWJ, Kahn RS, Luijten PR, Pol HEH. Glutamate in Schizophrenia: A Focused Review and Meta-Analysis of 1H-MRS Studies. Schizophr Bull. 2011; sbr069. doi:10.1093/schbul/sbr069

5. Schobel SA, Chaudhury NH, Khan UA, Paniagua B, Styner MA, Asllani I, et al. Imaging Patients with Psychosis and a Mouse Model Establishes a Spreading Pattern of Hippocampal Dysfunction and Implicates Glutamate as a Driver. Neuron. 2013;78: 81–93. doi:10.1016/j.neuron.2013.02.011
6. Dehghan A, Dupuis J, Barbalic M, Bis JC, Eiriksdottir G, Lu C, et al. Meta-analysis of genome-wide association studies in >80 000 subjects identifies multiple loci for C-reactive protein levels. Circulation. 2011;123: 731–738. doi:10.1161/CIRCULATIONAHA.110.948570
7. Vaez A, Jansen R, Prins BP, Hottenga J-J, de Geus EJC, Boomsma DI, et al. In Silico Post Genome-Wide Association Studies Analysis of C-Reactive Protein Loci Suggests an Important Role for Interferons. Circ Cardiovasc Genet. 2015;8: 487–497. doi:10.1161/CIRCGENETICS.114.000714
8. Warde-Farley D, Donaldson SL, Comes O, Zuberi K, Badrawi R, Chao P, et al. The GeneMANIA prediction server: biological network integration for gene prioritization and predicting gene function. Nucleic Acids Res. 2010;38: W214–W220. doi:10.1093/nar/gkq537
9. Hwang Y, Kim J, Shin JY, Kim JI, Seo JS, Webster MJ, et al. Gene expression profiling by mRNA sequencing reveals increased expression of immune/inflammation-related genes in the hippocampus of individuals with schizophrenia. Transl Psychiatry. 2013;3: e321. doi:10.1038/tp.2013.94
10. Schizophrenia Working Group of the Psychiatric Genomics Consortium. Biological insights from 108 schizophrenia-associated genetic loci. Nature. 2014;511: 421–427. doi:10.1038/nature13595
